# Phenylalanine Tolerance over Time in Phenylketonuria: A Systematic Review and Meta-Analysis

**DOI:** 10.3390/nu15163506

**Published:** 2023-08-08

**Authors:** Alex Pinto, Fatma Ilgaz, Sharon Evans, Esther van Dam, Júlio César Rocha, Erdem Karabulut, Mary Hickson, Anne Daly, Anita MacDonald

**Affiliations:** 1 Department of Dietetics, Birmingham Women’s and Children’s Hospital, Birmingham B4 6NH, UK; evanss21@me.com (S.E.); a.daly3@nhs.net (A.D.); anita.macdonald@nhs.net (A.M.); 2 School of Health Professions, Faculty of Health, University of Plymouth, Plymouth PL4 6AB, UK; mary.hickson@plymouth.ac.uk; 3 Department of Nutrition and Dietetics, Faculty of Health Sciences, Hacettepe University, 06100 Ankara, Turkey; fatma.celik@hacettepe.edu.tr; 4 Beatrix Children’s Hospital, University of Groningen, University Medical Center, 9700 RB Groningen, The Netherlands; e.van.dam@umcg.nl; 5 Nutrition and Metabolism, NOVA Medical School, Faculdade de Ciencias Medicas, Universidade Nova de Lisboa, 1169-056 Lisboa, Portugal; rochajc@nms.unl.pt; 6 CINTESIS@RISE, NOVA Medical School, Faculdade de Ciências Médicas, NMS, FCM, Universidade Nova de Lisboa, 1169-056 Lisboa, Portugal; 7 Reference Centre of Inherited Metabolic Diseases, Centro Hospitalar Universitario de Lisboa Central, 1169-045 Lisboa, Portugal; 8 Department of Biostatistics, Faculty of Medicine, Hacettepe University, 06100 Ankara, Turkey; ekarabul@hacettepe.edu.tr

**Keywords:** phenylketonuria, PKU, protein, protein tolerance, protein substitute, natural protein, phenylalanine, phenylalanine tolerance, medical formula, amino acids

## Abstract

In phenylketonuria (PKU), natural protein tolerance is defined as the maximum natural protein intake maintaining a blood phenylalanine (Phe) concentration within a target therapeutic range. Tolerance is affected by several factors, and it may differ throughout a person’s lifespan. Data on lifelong Phe/natural protein tolerance are limited and mostly reported in studies with low subject numbers. This systematic review aimed to investigate how Phe/natural protein tolerance changes from birth to adulthood in well-controlled patients with PKU on a Phe-restricted diet. Five electronic databases were searched for articles published until July 2020. From a total of 1334 results, 37 articles met the eligibility criteria (*n* = 2464 patients), and 18 were included in the meta-analysis. The mean Phe (mg/day) and natural protein (g/day) intake gradually increased from birth until 6 y (at the age of 6 months, the mean Phe intake was 267 mg/day, and natural protein intake was 5.4 g/day; at the age of 5 y, the mean Phe intake was 377 mg/day, and the natural protein intake was 8.9 g/day). However, an increase in Phe/natural protein tolerance was more apparent at the beginning of late childhood and was >1.5-fold that of the Phe tolerance in early childhood. During the pubertal growth spurt, the mean natural protein/Phe tolerance was approximately three times higher than in the first year of life, reaching a mean Phe intake of 709 mg/day and a mean natural protein intake of 18 g/day. Post adolescence, a pooled analysis could only be performed for natural protein intake. The mean natural protein tolerance reached its highest (32.4 g/day) point at the age of 17 y and remained consistent (31.6 g/day) in adulthood, but limited data were available. The results of the meta-analysis showed that Phe/natural protein tolerance (expressed as mg or g per day) increases with age, particularly at the beginning of puberty, and reaches its highest level at the end of adolescence. This needs to be interpreted with caution as limited data were available in adult patients. There was also a high degree of heterogeneity between studies due to differences in sample size, the severity of PKU, and target therapeutic levels for blood Phe control.

## 1. Introduction

Phenylketonuria (PKU) is an amino acid disorder caused by a deficiency in the enzyme phenylalanine (Phe) hydroxylase, which catalyses the conversion of Phe into tyrosine. It is a rare condition, with an estimated global prevalence of 1:23,930 live births (range: 1:4500 (Italy)–1:125,000 (Japan)) [1]. Infants are commonly identified via newborn screening in the first two weeks of life with blood Phe levels that are consistently >360 µmol/L [2]. If left untreated, the accumulation of Phe in the blood and brain results in global developmental delay, with severe irreversible intellectual disability accompanied by eczematous rash, autism, seizures, ataxia, and motor deficits [2,3].

Based on pre-treatment blood Phe levels, patients are classified into three different phenotypes that require treatment: mild PKU with pre-treatment Phe levels of 360–600 μmol/L, moderate PKU with pre-treatment Phe > 600–1200 μmol/L, and classical PKU with pre-treatment Phe > 1200 μmol/L [3]. The traditional treatment of PKU is a lifelong Phe-restricted diet as there is negligible or very minimal capacity to oxidise Phe [4]. The extent of restriction depends on several factors, including residual PAH activity, age, growth status, energy balance, protein substitute dose, adherence to protein substitute use, and target therapeutic blood Phe levels [5]. However, patients with classical PKU usually tolerate Phe less than 500 mg /day or natural protein ≤10 g /day, mostly derived from plant sources such as fruits and vegetables. A prescribed natural protein/Phe intake may be provided via a daily Phe allowance whereby patients calculate the Phe content of all foods eaten. Alternatively, it may be allocated as protein or a Phe exchange system (0.5 or 1 g protein exchanges or 15, 20, 25, or 50 mg Phe exchanges) [5]. The remaining protein requirements are usually supplied by a Phe-free/low-Phe protein substitute derived from L-amino acids or casein glycomacropeptide. It is initially provided in infancy as a Phe-free infant formula and later as a semi-solid, drink, or tablets. Special low-protein foods (e.g., bread and pasta) provide energy and aid dietary adherence.

In addition to the traditional Phe-restricted diet, two pharmacological treatments, sapropterin dihydrochloride (chaperone) and pegvaliase (enzyme substitution therapy), have been licenced for the treatment of PKU [6,7]. Sapropterin dihydrochloride is a synthetic form of tetrahydrobiopterin (BH4). It allows the liberalisation of dietary restrictions in responsive patients (e.g., by increasing Phe tolerance by 2.5-to 4-fold, with the cessation of protein substitute use in >50% of responsive patients) [8]. However, this therapy is only effective in individuals with residual enzyme activity, usually those with milder PKU, and most patients still require some dietary restrictions [9,10]. Pegvaliase, an injectable pegylated enzyme based on Phe ammonia lyase (pPAL), was approved by the FDA in 2018 for adult patients only [7]. This treatment option enables many patients to consume a normal protein intake while maintaining blood Phe levels in the target therapeutic range [11].

In clinical practice, individual Phe (or natural protein) tolerance is defined as the amount of Phe (or natural protein) that maintains blood Phe concentrations within a target therapeutic range [12]. Phe tolerance is pragmatically determined by titrating Phe/natural protein intake until target blood Phe concentrations are consistently achieved. Generally, if blood Phe levels are above or below target Phe levels, the Phe intake will be lowered or increased accordingly. However, Phe is an indispensable aromatic amino acid which is essential for the synthesis of tyrosine, catecholamines, and melanin and to maintain Phe homeostasis [13]. Thus, the amounts provided should not be over-restricted [14]; its deficiency causes anorexia, alopecia, and poor growth [15].

There are limited data on lifelong Phe/natural protein tolerance in PKU. This is associated with historical changes in treatment policy. In the 1960s and 1970s, dietary treatment was discontinued at 8 years of age, as some researchers considered that brain development was complete and no further neurological deterioration followed [16]. However, this practice led to IQ deterioration, so dietary treatment was then extended until late adolescence, and from the 1990s, it was recommended that treatment should continue for life [17]. Over time, the recommendations for target blood Phe levels have also lowered. This has intensified the stringency of dietary management required and consequently increased the challenges of lifelong dietary adherence, particularly from the start of adolescence. Overall, it is not clear if any variation in Phe tolerance is related to the changes in clinical recommendations and practices (e.g., lowered target blood Phe levels) or because of intrinsic tolerance. Little is also known about differences between the prescribed and actual Phe/natural protein intake consumed, although there is some evidence to suggest this may be wide [18].

When considering the Phe content of food, for most foods, the amount that provides 1 g of protein contains 5%, or 50 mg, of Phe. However, fruit and vegetables generally contain lower amounts of Phe per 1 g of protein, and there is a suggestion that these have a lesser effect on increasing blood Phe levels [18]. Studies have consistently demonstrated that the unmeasured use of some fruits and vegetables with a Phe content ≤ 75 mg/100 g does not adversely influence blood Phe levels despite an increase in dietary Phe intake [19,20,21]. Consequently, although some countries calculate Phe from all foods, others permit all fruits and vegetables that contain Phe ≤ 75 mg/100 g without consideration of their Phe contents. If this Phe intake is not calculated as part of dietary assessments, it will underestimate the total Phe consumed. In addition, Phe analysis is not available for all foods, so it is difficult to calculate exact Phe intake with accuracy.

Due to the rarity of PKU, research studies commonly recruit small numbers of subjects. A systematic review identifying studies examining protein/Phe tolerance would enable the combined results to be collated and provide a better understanding of Phe/natural protein tolerance. The primary objective of this systematic review and meta-analysis is to examine how dietary Phe prescription varies with age and secondly to compare prescribed protein/Phe intake with actual protein/Phe intake in PKU.

## 2. Materials and Methods

This study was conducted by using the Preferred Reporting Items for Systematic Reviews and Meta-Analyses (PRISMA) current guidelines [22]. The protocol was developed by the authors and is registered to PROSPERO under the record number CRD42019136416.

### 2.1. Literature Search

A systematic literature review was performed in five electronic databases (including Medline/PubMed, Web of Science, Embase, Scopus, and Central Cochrane Library) for any articles published in English, with no date restriction. The following keywords were used in the PubMed search query: (Phenylketonurias [Mesh] OR PKU[tiab] OR phenylketonuria*[tiab] OR “BH4 def*”[tiab] OR “phenylalanine def*”[tiab] OR “phenylalanine hydroxylase def*”[tiab] OR “PAH def*”[tiab]) AND (Phenylalanine[Mesh] OR Proteins[Mesh] OR phe[tiab] OR phenylalanine[tiab] OR protein*[tiab]) AND (“Drug Tolerance”[Mesh] OR toleran*[tiab] OR “intake food”[Mesh] OR intake*[tiab]). For the remaining four databases, these main terms were customised. The last search was completed on 7 July 2020.

Additional resources were identified by searching the proceedings of national and international scientific meetings for inborn metabolic diseases, including the Society for the Study of Inborn Errors of Metabolism (SSIEM), International Congress of Inborn Errors of Metabolism (ICIEM), and the British Inherited Metabolic Disease Group (BIMDG).

### 2.2. Study Selection

The PICO (population, intervention, comparison, and outcomes) method was applied to formulate the review question and to determine the eligibility criteria. All retrospective and prospective longitudinal studies, randomised controlled trials, observational studies (case–control, cohort, and cross-sectional), letters, systematic reviews, conference abstracts, and PhD reports conducted in patients with PKU who were aged < 50 years, identified via newborn screening and then diagnosed via a confirmatory blood Phe concentration > 360 µmol/L, and continuously treated with a Phe-restricted diet starting within the first month of life were included.

Preclinical studies (in vitro and in vivo studies conducted on cell cultures or animals), non-original research (such as expert opinions and narrative reviews), case studies, and studies without a clear definition of dietary treatment (i.e., low protein/low Phe diet with Phe-free/low Phe protein substitute) or lacking sufficient data on age and prescribed and/or actual protein intakes were excluded. Studies were also considered for exclusion if (1) the median or majority of the blood Phe levels were out of the target range, (2) the patients received a late diagnosis, (3) untreated patients with PKU whose dietary treatment was not started within the first month of age, (4) the patients were not receiving continuous dietary treatment, (5) the patients had maternal PKU, (6) the patients were treated with large neutral amino acids, sapropterin or pegvaliase, and (7) the sample size was lower than 8 patients.

Patients who did not use a protein substitute, followed a normal diet, or whose dietary treatment was interrupted and patients who were not adherent to their treatment were also removed from the analyses when known. For studies involving sapropterin treatment, only baseline data on dietary intakes before the initiation of sapropterin therapy were included in the analysis.

Two independent reviewers (A.P. and F.I.) screened titles and abstracts according to the eligibility criteria. The full texts of all potentially relevant articles were reviewed. Disagreements were resolved through discussions with other authors.

### 2.3. Outcome Measures

To investigate the changes in dietary protein/Phe intake over time, prescribed or self-reported (actual) intakes of Phe (mg/day or mg/kg/day), natural protein (g/day or g/kg/day), and total protein (natural protein plus protein equivalent from protein substitute, expressed as g/day or g/kg/day) by age were selected as primary outcomes. Secondary outcomes were energy intake, protein substitute intake (expressed as protein equivalent g/kg/day), and measures of metabolic control (e.g., blood Phe levels compared with target blood Phe), where available. Studies were included based on primary outcome measures.

### 2.4. Data Extraction

The data were collected by two independent authors (A.P. and F.I.) using a standardised data extraction form. The information extracted included: (1) study characteristics (authors, publication year, country/setting, and design of the study), (2) description of population (sample size, age, gender, phenotype, and ethnic origin), (3) data regarding dietary treatment (a brief description of dietary treatment, level of Phe-restriction, types of Phe-free/low-Phe protein substitutes, allowance of Phe from vegetables/fruits or free foods, the use of an exchange system, and method of dietary assessment), (4) outcome variables (prescribed, actual protein, Phe, protein equivalent intake from protein substitutes, energy intake, and weight), and (5) data about metabolic control (blood Phe levels, target therapeutic ranges, and the centre’s definition of metabolic control). When further information (e.g., missing data regarding dietary intake or age) or clarification were needed, only the authors of papers from 2015 and onwards were contacted.

### 2.5. Quality Appraisal and Risk of Bias Assessment

Two reviewers (A.P. and F.I.) independently assessed the quality of the evidence and the risk of bias of the included studies using the two quality assessment tools developed by the National Institutes of Health (NIH) for “observational cohort and cross-sectional studies”, and “controlled intervention studies” [23]. All the studies were assigned a “yes”, “no”, “cannot determine”, “not reported”, or “not applicable” for each of the 12–14 criteria outlined in the appraisal tools. Based on the ratings, an overall judgement was made regarding the quality of each study: (1) “good quality” if the study had a minimal risk of bias, (2) “fair quality” if the study was susceptible to some bias but was not deemed sufficient to invalidate its results, and (3) “poor quality” if the study raised substantial concerns. Differing ratings between reviewers were discussed until consensus was reached.

### 2.6. Data Analysis

“The Preferred Reporting Items for Systematic Reviews and Meta-Analyses (PRISMA)” guidelines were followed. All variables were summarised in descriptive tables using means ± SDs, medians, and range/IQRs as appropriate.

To answer the research question: “*does natural protein/Phe intake change over the life span?*”, a meta-analysis was performed synthesising the available longitudinal data on protein and Phe intakes, if a minimum of 2 studies were available. For effect size measure, we used the “mean change per year”. The heterogeneity between studies was estimated using the *I*^2^ statistic, with values of 25%, 50%, and 75% considered to indicate low, medium, and high levels of heterogeneity, respectively. A random-effects model was used to calculate pooled estimates using the R software “meta (version 6.5-0)” package (R foundation for statistical computer, Vienna, Austria) [24]. A *p* value less than 0.05 was considered statistically significant.

Studies that could not be included in the meta-analyses (i.e., due to missing data regarding self-reported intakes or sample size) were analysed qualitatively. Secondary outcomes were analysed qualitatively (no meta-analysis). A subgroup analysis of main outcomes was performed according to gender.

## 3. Results

### 3.1. Study Selection

**Figure 1:**
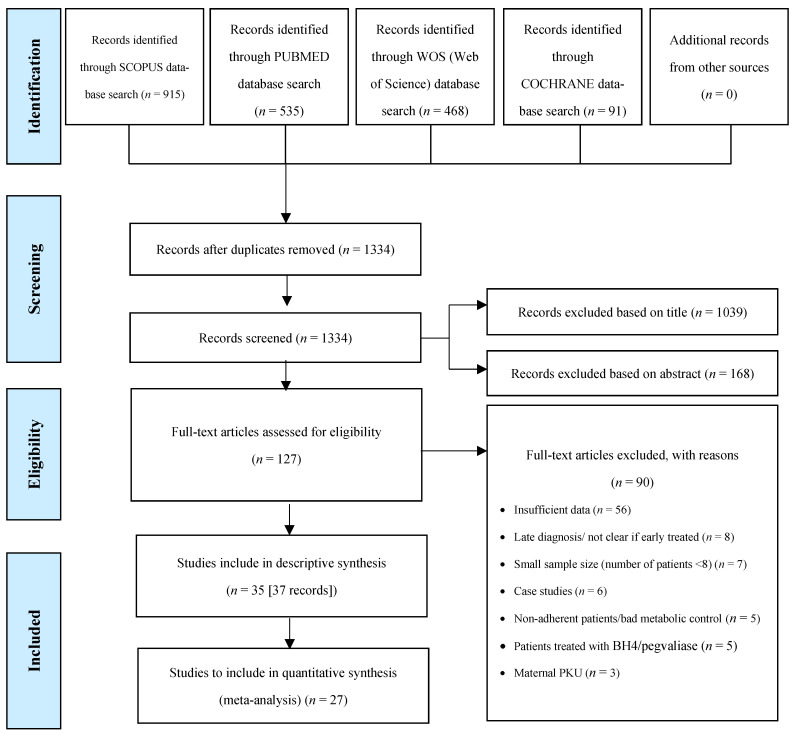
Study selection process according to the Preferred Reporting Items for Systematic Reviews and Meta-Analysis (PRISMA) flow chart.

We identified 1334 published articles, after duplicate papers were removed from the database searches. In total, 37 articles were eligible for inclusion in the systematic review, describing a total of 35 studies (Figure 1). Two articles from Rocha et al. [25,26] and two from Rohde et al. [19,20] reported data on the same participants; hence, patient data from each of the two authors were evaluated together. All studies were included in the descriptive synthesis. Twenty-seven of thirty-seven studies were included in the meta-analyses (*n* = 7, for gender analysis). The remaining 10 studies were excluded from the meta-analysis due to missing data regarding self-reported dietary intakes and/or a limited number (*n* = 1) of patients in each age group.

### 3.2. Study Characteristics

Table 1 presents the main characteristics of the 37 studies included in this review. In total, data were collected on 2464 patients with PKU describing their natural protein tolerance. The majority (*n* = 19) were longitudinal studies (retrospective or prospective). There were ten randomised controlled trials, six cross-sectional studies, and one case–control study. Most studies were conducted in Europe (Europe, *n* = 30; USA, *n* = 2; Australia, *n* = 2; Canada, *n* = 1).

The studies were mainly conducted in children with PKU. Only six studies reported data from adults (>18 years) [19,25,26,27,28,29,30]. Of 37 studies, 28 provided self-reported dietary intakes [19,20,25,26,27,29,31,32,33,34,35,36,37,38,39,40,41,42,43,44,45,46,47,48,49,50,51,52], 6 described dietary prescriptions [18,41,42,53,54,55,56], and 2 study reported both [57]. From the available data (*n* = 14 studies), the patients were mainly of Caucasian origin. In the 11 studies that provided data on phenotype, the majority of study samples consisted of patients diagnosed with classical PKU (cPKU) and mild hyperphenylalaninaemia (mHPA) (*n* = 480 cPKU, *n* = 364 mHPA, and *n* = 194 mild PKU (mPKU) and *n* = 226 moderate PKU (moPKU). Data on sex were available in 32 studies, showing that male and female participants were equally represented (*n* = 996 male vs. *n* = 996 female).

**Table 1:** Main characteristics of included studies.

Reference	Country, Setting	Study Design	PKU Phenotype	Sample Size	Age		Gender (M:F)	Ethnic Origin
					Mean (SD)	Median (Range)		
Acosta et al., 1998 [31]	USA, Multicentre	Prospective, longitudinal	cPKU	35	13.7 (1.9) days	N/A	20:15	33 Caucasian; 2 unknown
Aldámiz-Echevarría et al., 2014 [32]	Spain, 14 centres	Retrospective; longitudinal; multicenter	mildHPA mPKU moPKU cPKU All types	226 43 78 158 505 ^a^	N/A	(1–18 years)	236:269	Caucasian
Aldámiz-Echevarría et al., 2013 [33]	Spain, 13 centres	Retrospective; longitudinal	mPKU moPKU cPKU All types	24 99 15 138 ^b^	Pre-sapropterin Baseline: 2-y FU: 5.0 y (4.6) 5-y FU: 5.2 y (3.1) Diet Baseline: 2-y FU: 5.0 y 5-y FU: 5.0 y Diet Final: 2-y FU: 7.0 y 5-y FU: 10.2 (3.0) y	N/A	Pre-sapropterin Baseline: 2-y FU 18:18 5-y FU 6:4 Diet Group: 2-y FU 36:36 5-y FU 12:8	Caucasian
Aldámiz-Echevarría et al., 2015 [34]	Spain, 14 centres	Retrospective; longitudinal	mPKU moPKU cPKU All types	15 42 9 66 ^c^	Pre-sapropterin Group: 16.9 (10.4) y Diet Group: 16.9 (10.3) y	(0–4 y)	Pre-sapropterin Group: 12:10 Diet Group: 24:20	Caucasian
Alm et al., 1986 [27]	Sweden, single centre	Prospective; longitudinal	All types	23 ^d^	N/A	Final age: (8–19 y)	12:11	22 Caucasian; 1 Syrian
Daly et al., 2017 [35]	UK, single centre	Prospective; longitudinal	All types	22 ^e^	N/A	11 y (6–16 y)	13:9	19 white European; 3 Pakistani
Daly et al., 2019 [36]	UK, 2 centres	Randomised; controlled; crossover	All types	19 ^f^	N/A	10 y (6–16 y)	7:11	17 white European; 2 Pakistani
Dobbelaere et al., 2003 [37]	France, single centre	Cross-sectional; longitudinal	All types	20	4.5 (1.6) y	(8 mo–7 y)	9:11	N/A
Evans et al., 2017 [38]	Australia, single centre	Prospective; longitudinal	All types	32	9.2 (4.7) y	(0.8–18 y)	10:22	N/A
Evans et al., 2018 [53]	UK, single centre	Retrospective; longitudinal	All types	31	N/A	Baseline: 20 wk (13–37 wk) Total: (3–24 mo)	16:15	N/A
Evans et al., 2019 [39]	UK, 3 centres	Open label; longitudinal; prospective; case–control	All types	20	N/A	Baseline: 4.3 mo (2.9–6.6 mo) Total: (3–24 mo)	14:6	Caucasian
Evers et al., 2018 [28]	The Netherlands 2 centres	Retrospective; cohort	All types	39 ^g^	Pre-sapropterin Group: 13.1 (9.2) y Diet Group: 13.0 (9.2) y	Pre-sapropterin Group: (2.8–33.7 y) Diet Group: (1.4–33.4 y)	Pre-sapropterin Group: 5:13 Diet Group: 7:14	N/A
Ferguson et al., 1996 [54]	UK, Multicentre	Randomised controlled trial	All types	20 ^h^	N/A	(9–15 y)	13:7	N/A
Giovaninni et al., 2014 [40]	Italy, 1 centre	Randomised-controlled	mildHPA mPKU cPKU All types	60 40 15 115	mHPA: 9.3 (3.3) y cPKU + mPKU: 9.2 (3.4) y	N/A	50:65	N/A
Gökmen-Özel et al., 2011 [41]	UK, 2 centres	Randomised-controlled; crossover	All types	14	N/A	6.3 (3.0–9.7) y	12:2	13 Caucasian; 1 Asian
Green et al.; 2019 [42]	UK, Multicentre	Cross-sectional (pooled analysis of 2 multicenter intervention studies)	All types	16	29.5 (11.2) y	N/A	7:9	N/A
Hoeksma et al., 2005 [43]	The Netherlands, 8 centres	Retrospective; longitudinal	All types	174 ^i^	N/A	(0–3 y)	N/A	Caucasian
Huemer et al., 2007 [44]	Austria, single centre	Prospective; longitudinal	All types	34	Baseline: 8.7 (3.9) y	(2 mo–15 y)	22:12	N/A
Kindt et al., 1983 [45]	Norway n/a	Prospective; longitudinal	All types	16	N/A	End of the study: (2–6 y)	7:9	N/A
MacDonald et al., 2006 [46]	UK, single centre	Randomised; prospective; crossover	All types	25	6.0 (2.5) y	6 (2–10) y	11:14	N/A
MacDonald et al.; 2003 [18]	UK, single centre	Randomised; crossover	All types	16 ^j^	5.3 (3.1) y	4.5 (2–11) y	4:12	15 Caucasian; 1 mixed ethnicity
MacDonald et al., 1996 [57]	UK, single centre	Longitudinal observational study	All types	19	6.6 (4.9) y	4 (1–16) y	15:4	Caucasian
Pinto et al., 2019 [29]	Portugal, single centre	Retrospective; longitudinal	mildHPA mPKU cPKU All types	10 23 7 40	17 y	(12–29 y)	24:16	Caucasian
Ponzone et al., 2008 [47]	Italy, single centre	Retrospective; longitudinal	mildHPA mPKU moPKU cPKU All types	5 6 4 7 22	8.5 (4.3) y	8 (1.7–14.7) y	10:12	N/A
Rocha et al., 2012 & 2013 [25,26]	Portugal, single centre	Cross-sectional	mildHPA mPKU cPKU All types	18 42 29 89	14.4 (6.6) y	(3–30 y)	48:41	Caucasian
Rohde et al., 2012 & 2014a [19,20]	Germany, single centre	Randomised; cross-over	All types	19	4.7 (2.1) y	(2–10) y	N/A	N/A
Rohde et al., 2015 [48]	Germany 10 centres	Retrospective; cross-sectional	All types	149	7 (6.6) y	7 (1–15) y	77:72	N/A
Rohde et al., 2014b [49]	Germany, Multicentre	Cross-sectional	All types	67	N/A	(6–45) y	N/A	N/A
Schulpis et al., 2013 [50]	Greece, single centre	Prospective; cohort	All types	30	5.0 (3.2) y	N/A	15:15	N/A
Stockler-Ipsiroglu et al., 2015 [58]	Canada, single centre	Retrospective chart review	mildHPA mPKU moPKU cPKU All types	4 1 1 5 11	5.4 (4.8) y	4.5 y (1 mo–15.5 y)	7:4	N/A
Sweeney et al., 2012 [30]	Australia, single centre	Phase I: Randomised controlled Phase II: Prospective cohort	moPKU cPKU All types	2 17 19	10.5 (5.7) y	9.5 y (1.5–20.5) y	6:13	N/A
Thiele et al., 2017 [51]	Germany, 2 centres	Retrospective; longitudinal	mildHPA cPKU All types	41 183 224	N/A	(0–18 y)	119:105	Caucasian
Trefz et al., 2009 [52]	Germany, Poland, Spain, USA, 15 centres	International; double-blind randomised; placebo controlled	All types	45 ^k^	Pre-sapropterin Baseline: 7.7 (2.8) y Placebo Baseline: 7.1 (2.0) y Total: 7.4 y	(4–12 y)	Pre-sapropterin Group: 20:13 Placebo: 6:6	N/A
van Spronsen et al., 2009 [55]	The Netherlands, 8 centres	Retrospective; longitudinal	All types	213	N/A	(1 mo–10 y)	N/A	N/A
Wendel et al., 1990 [56]	Germany, single centre	Retrospective; longitudinal	All types	139	N/A	(1–6 y)	66:73	N/A

Abbreviations: mildHPA, mild hyperphenylalaninaemia; mPKU, mild phenylketonuria; moPKU, moderate phenylketonuria; cPKU, classical phenylketonuria; mo; months; y, years; FU, follow-up; sapropterin, tetrahydrobiopterin; M:F, male:female; N/A, not available. ^a^ From a total of 505 patients (236 M, 269 F), only 98 (53 M, 45 F) were included in the nutritional analysis. ^b^ There were two cohorts in this study: (1) sapropterin-treated group followed for 2 years and 5 years, and (2) a diet-only treated group followed for 2 years and 5 years. Only data at baseline (pre-sapropterin treatment) were included in the analysis in the sapropterin-treated group. Phenotype distribution of patients who were followed for 2 years and 5 years in the sapropterin group were as follows: 7 mPKU, 24 moPKU, 5 cPKU (N = 36); and 1 mPKU, and 9 moPKU (N = 10), respectively. Phenotype distribution of diet-only treated patients who were followed for 2 years and 5 years was as follows: 14 mPKU, 48 moPKU, and 10 cPKU (N = 72); 2 mPKU and 18 moPKU (N = 20), respectively. ^c^ There were two cohorts in this study: (1) sapropterin-treated group, and (2) diet-only-treated group. Only data at baseline (pre-sapropterin treatment) were included in the analysis in the sapropterin-treated group. Phenotype distribution of patients in the sapropterin- and diet-only-treated groups were as follows: 5 mPKU, 14 moPKU, and 3 cPKU (N = 22); and 10 mPKU, 28 moPKU, and 6 cPKU (N = 44), respectively. ^d^ From a total of 23 patients (12 M, 11 F), only 18 were included in the nutritional analysis. ^e^ There were two cohorts in this study: (1) patients (N = 13) using glycomacropeptide (CGMP) as a low-Phe protein substitute, and (2) patients (N = 9) using Phe-free amino acid mixtures (AA). After withdrawal of 1 patient from CGMP group, the study was completed with a total of 21 patients. ^f^ At baseline, 19 patients were enrolled. After the withdrawal of 1 patient (12 y old), the study was completed with a total of 18 patients. ^g^ There were two cohorts in this study: (1) a sapropterin-treated group (N = 21), and (2) diet-only-treated group (N = 19). Only data at baseline (pre-sapropterin treatment) were included in the analysis in the sapropterin-treated group. ^h^ At baseline, 20 patients were allocated, but 6/20 patients were excluded due to high blood Phe levels (serum Phe > 700 mmol/L), and 2/20 patients self-withdrew from study. The study was completed with 12 patients. ^i^ Due to insufficient dietary data, there were 41, 39, and 57 dropouts in the data on total protein, protein substitute, and natural protein intake, respectively, leaving 133, 135, and 117 subjects to be analysed. ^j^ After the withdrawal of a 2 y old girl, the study was completed with N = 15 patients. Prescribed dietary intake data at baseline was shown for N = 16. ^k^ Thirty-three patients received sapropterin treatment, and twelve patients received a placebo. Baseline dietary intake data was obtained for only 39 patients (N = 30 sapropterin-treated and N = 9 placebo).

### 3.3. Primary Outcomes: Changes in Phe, Natural Protein, and Total Protein Intakes throughout Lifetime

Intakes of Phe (mg/day or mg/kg/day; *n* = 21 studies), natural protein (g/day or g/kg/day; *n* = 28 studies), and total protein (g/day or g/kg/day; *n* = 29 studies) were measured in 29 studies. Only *n* = 14 studies for Phe, *n* = 19 studies for natural protein, and *n* = 18 studies for total protein intake were eligible for inclusion in the pooled analysis. The remaining studies were not included in the meta-analysis due to reporting prescribed intakes only or lacking information about sample size for each age group or the means, medians, or SDs of the intakes. However, data on these studies were included in the Supplementary Tables (Supplementary Tables S1–S4) to describe all available evidence on actual dietary intakes during the following four developmental periods: (1) infancy and young children (0–3 years), (2) pre-school and late childhood (4–10 years), (3) adolescence (11–18 years), and (4) adulthood. No datasets were excluded. Data on prescribed intakes from a total of nine studies [18,28,42,53,54,55,56,57] were also summarized in Supplementary Table S5.

**Figure 2:**
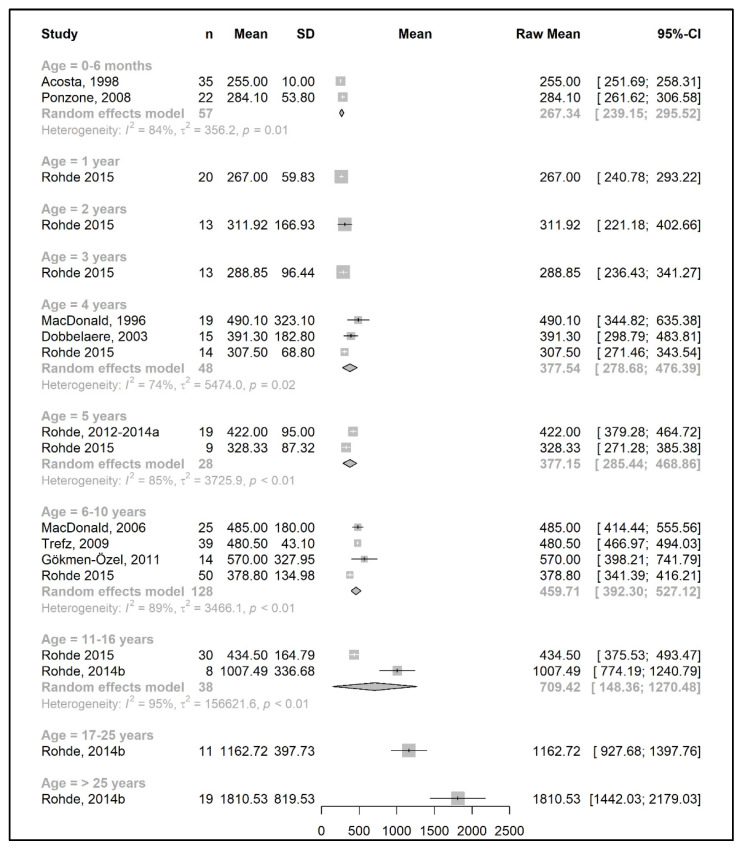
Phenylalanine intake (mg/day) of participants in the included studies [19,20,31,37,41,46,47,48,49,52,57].

**Figure 3:**
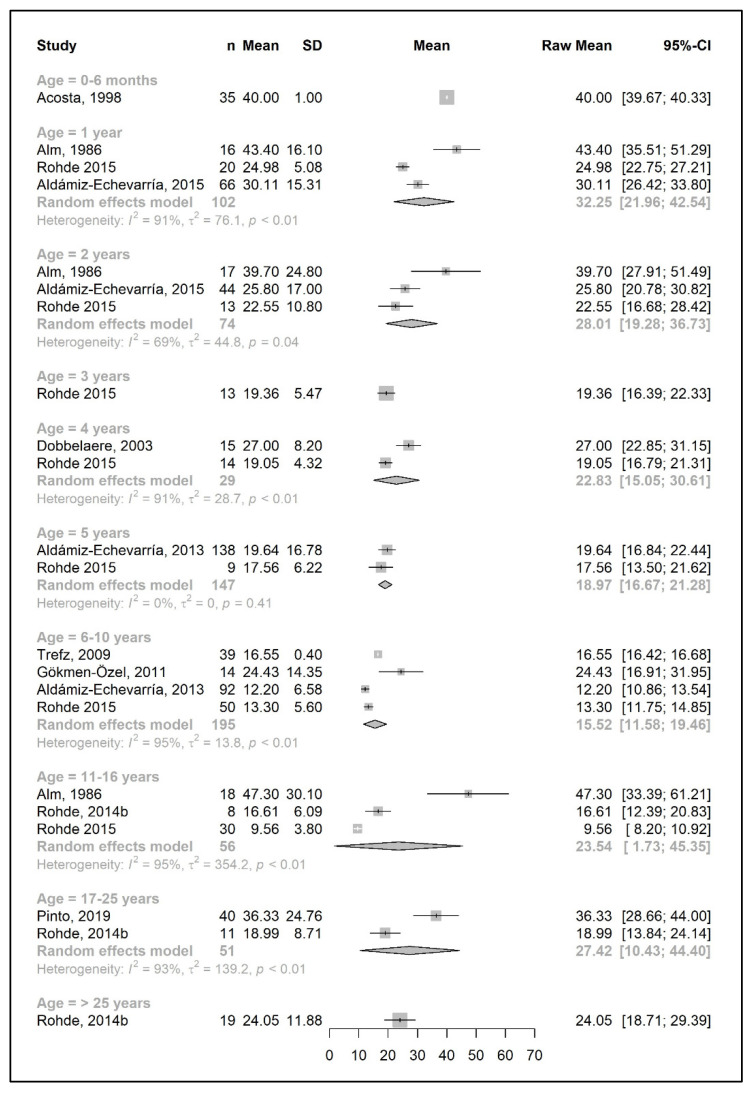
Phenylalanine intake per kg of body weight (mg/kg/day) of participants in the included studies [27,29,31,33,34,37,41,48,49,52].

**Figure 4:**
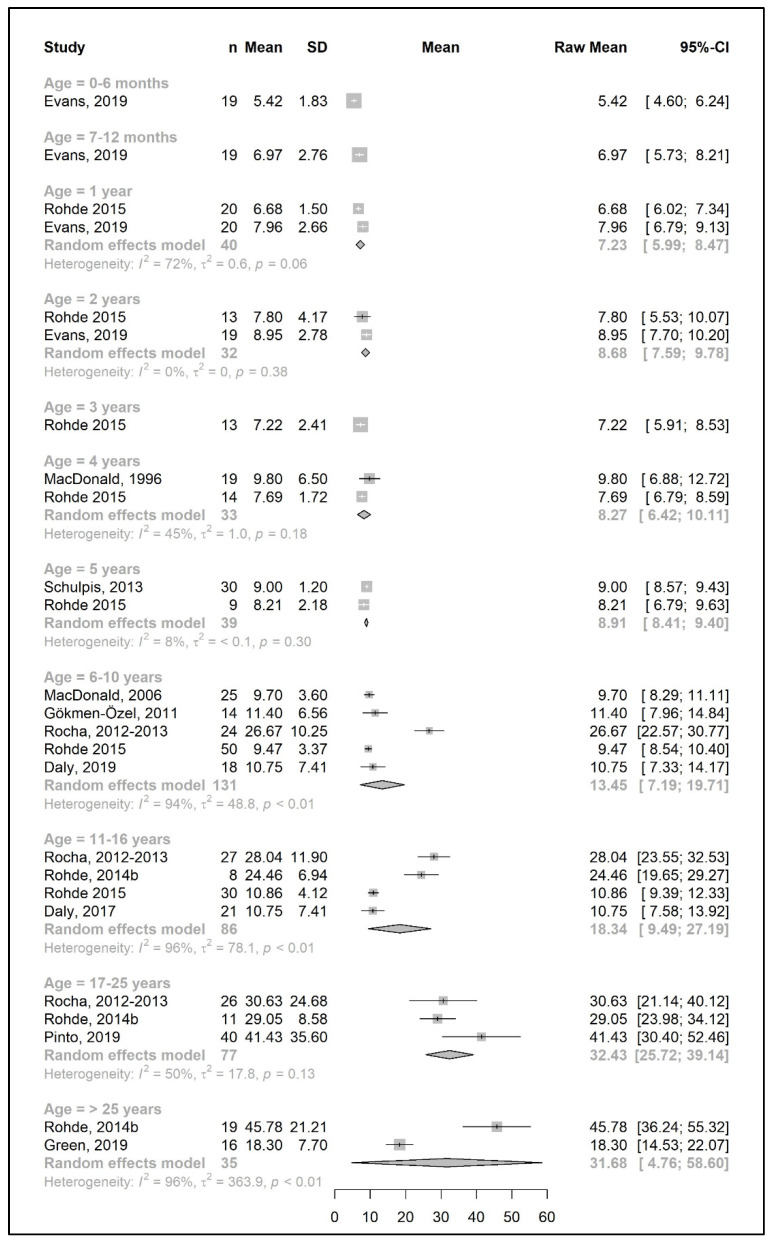
Natural protein intake (g/day) of participants in the included studies [25,26,29,35,36,39,41,42,46,48,49,50,57].

**Figure 5:**
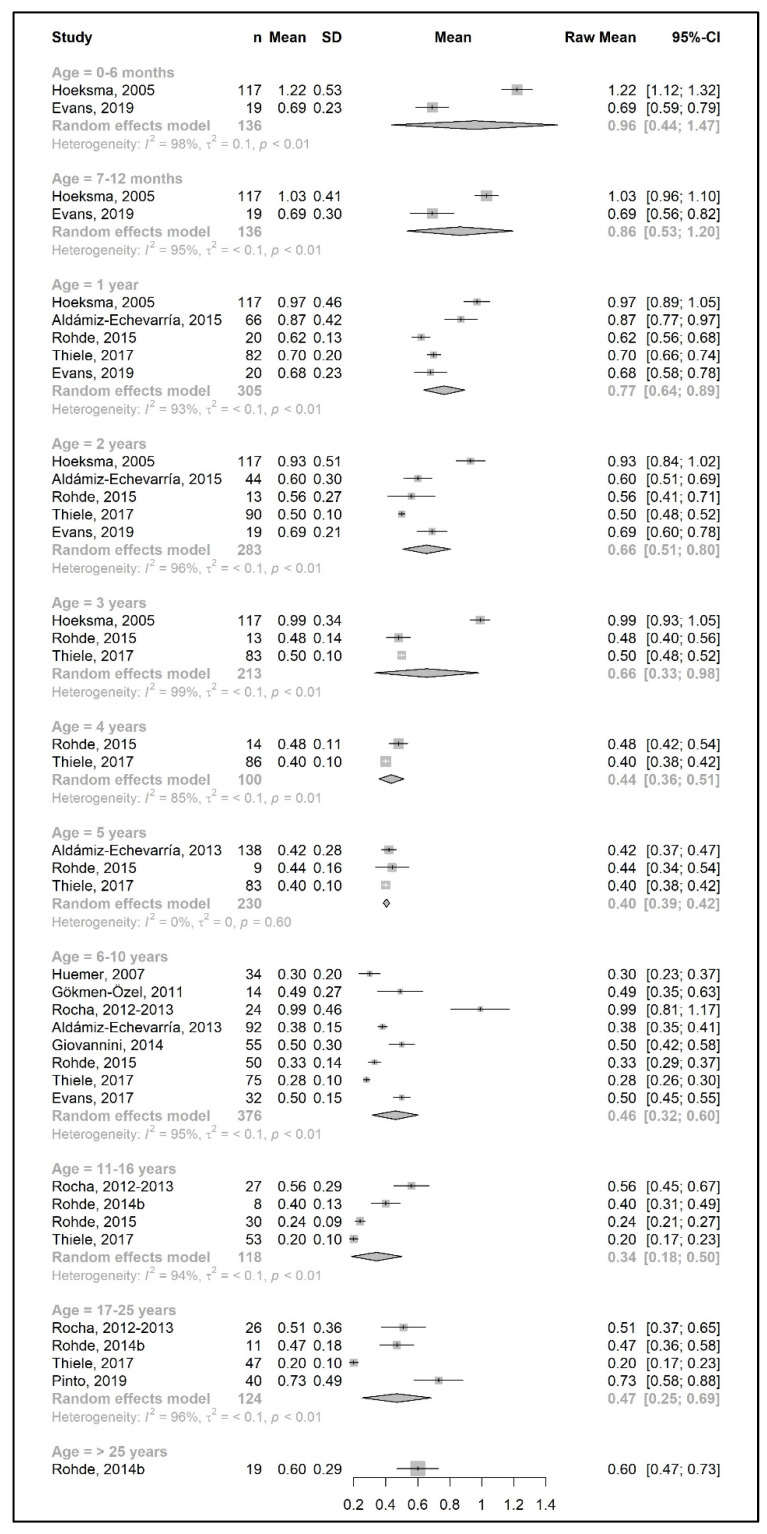
Natural protein intake per kg of body weight (g/kg/day) of participants in the included studies [25,26,29,33,34,38,39,40,41,43,44,48,49,51,52].

**Figure 6:**
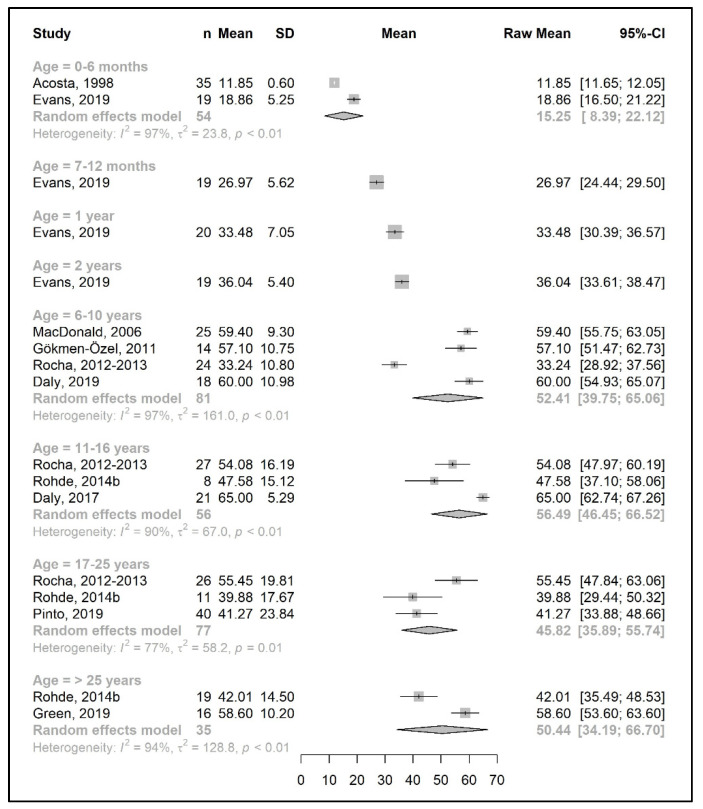
Protein equivalent intake from a protein substitute (g/day) of participants in the included studies [25,26,29,31,35,36,39,41,42,46,49].

**Figure 7:**
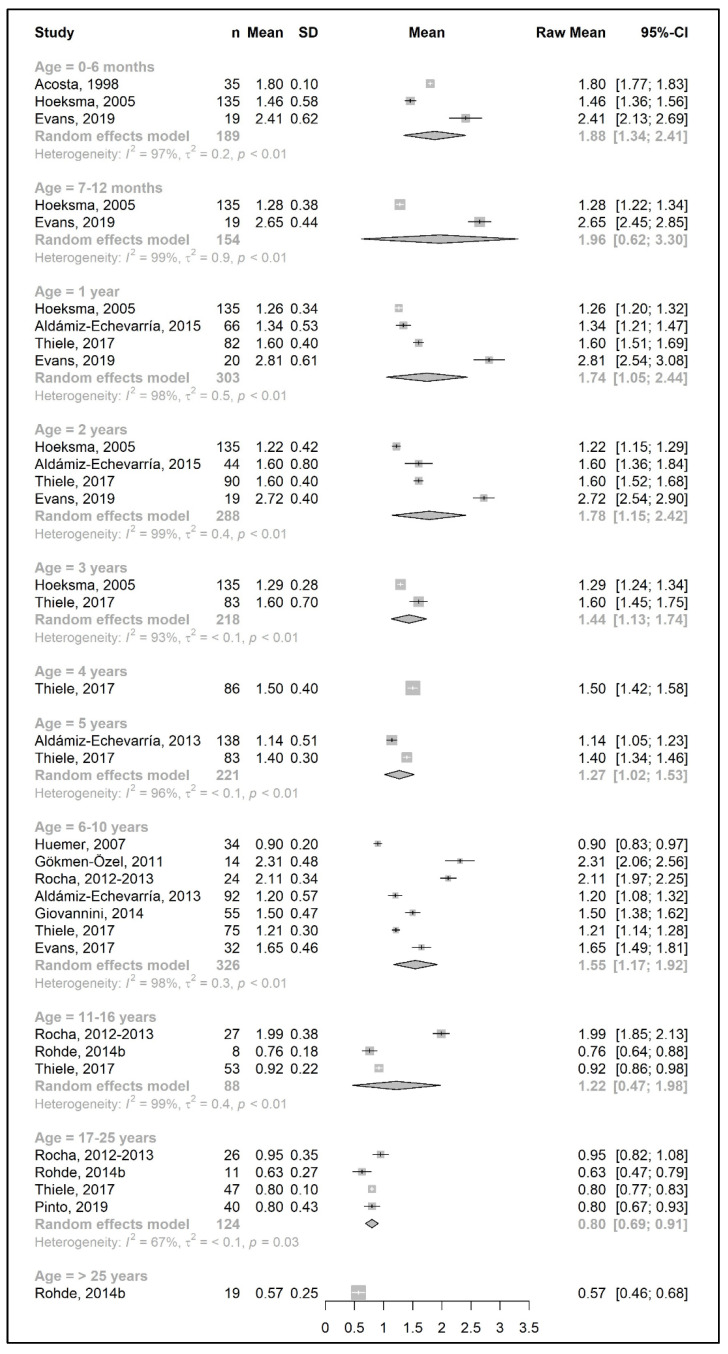
Protein equivalent intake from a protein substitute per kg of body weight (g/kg/day) of participants in the included studies [25,26,29,31,33,34,38,39,40,41,43,44,49,51].

**Figure 8:**
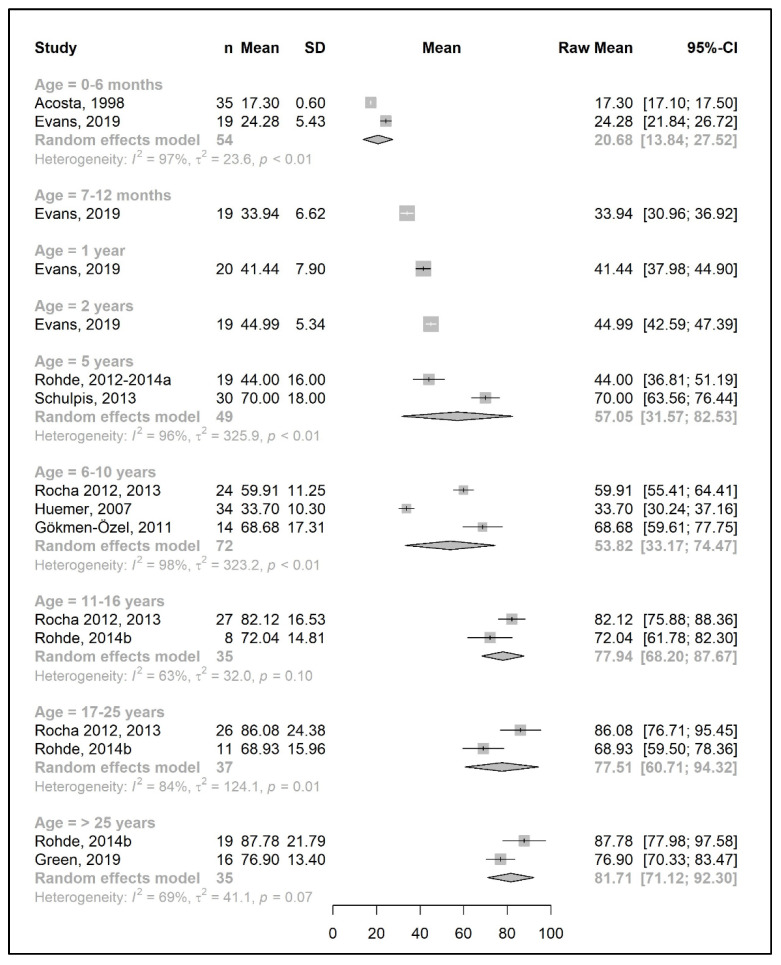
Total protein intake (g/day) of participants in the included studies [19,20,25,26,31,39,41,42,44,49,50].

**Figure 9:**
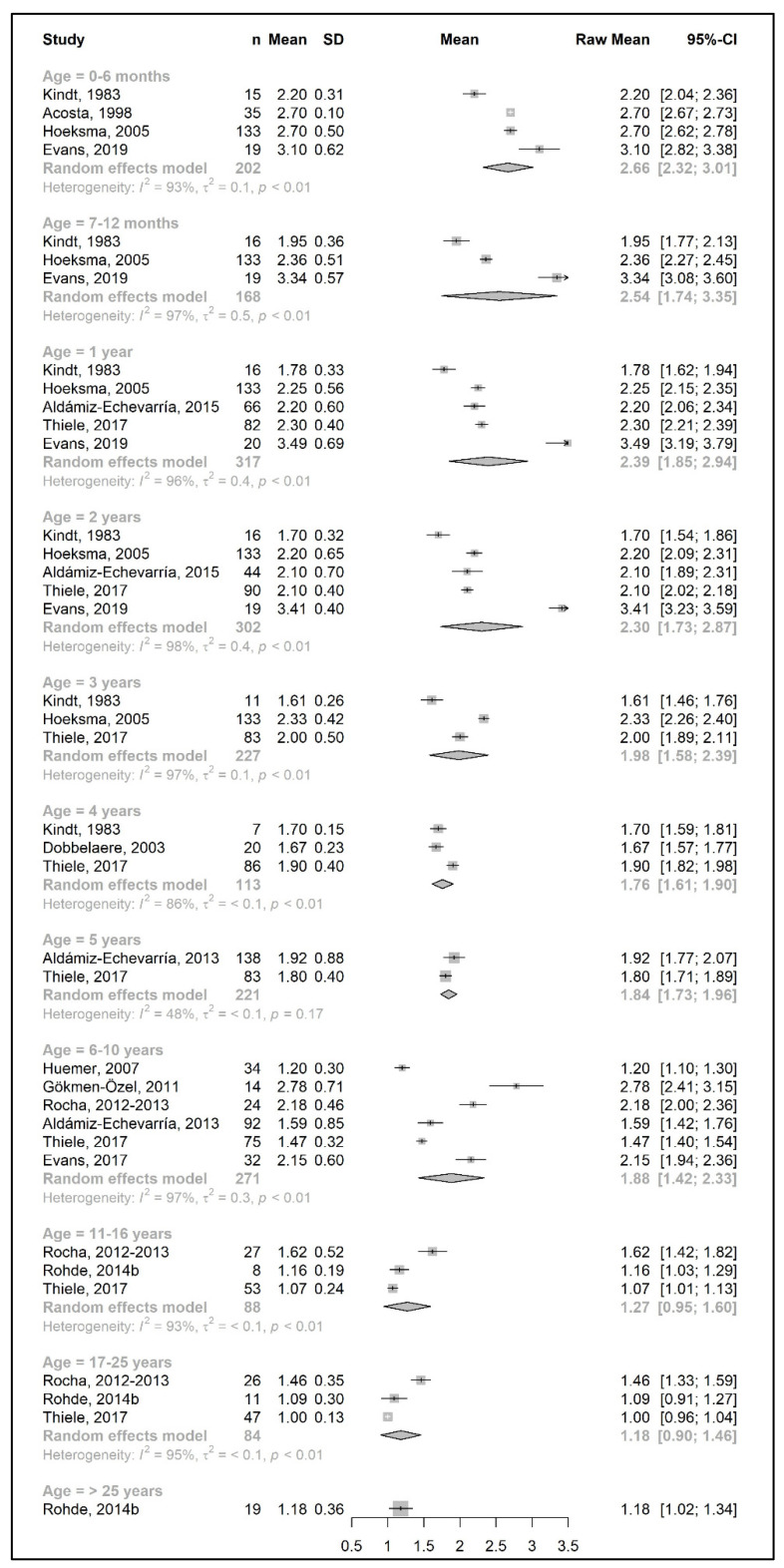
Total protein intake per kg of body weight (g/kg/day) of participants in the included studies [25,26,31,33,34,37,38,39,41,43,44,45,49,51].

The meta-analysis results are presented as raw means with confidence intervals (CIs) for each age group and are illustrated in the forest plots (Figure 2, Figure 3, Figure 4, Figure 5, Figure 6, Figure 7, Figure 8 and Figure 9). A meta-analysis could only be performed for age groups if there were at least two studies per age group. When there was only one study available, the mean actual intake from that study only is shown in the forest plot.

#### 3.3.1. Phenylalanine and Natural Protein Tolerance

Figure 2 and Figure 4 show results from the actual amounts of Phe and natural protein consumed each day. The mean intakes of Phe (mg/day) and natural protein (g/day) increased from birth until 6 years of age (267 mg Phe/day and 5.4 g natural protein/day at 6 months of age; 377 mg Phe/day and 8.9 g natural protein/day at 5 years of age) but decreased when expressed as g/kg/day, with 0.96 at 0–6 months of age and 0.40 at 5 y of age. However, the increase in natural protein/Phe tolerance per day (mg of Phe or g of protein) was more apparent at the beginning of late childhood (6–10 years), which was approximately 1.5-fold that of early childhood (2–5 years), with seven studies allowing for higher blood Phe target levels [27,42,44,45,48,51,54,57]. During the pubertal growth spurt, the mean Phe/natural protein tolerance was approximately three times higher than the Phe/natural protein tolerance in the first year of life, reaching a mean Phe intake of 709 mg/day and a natural protein intake of 18 g/day. Post adolescence, a pooled analysis could only be performed for natural protein intake. The mean natural protein tolerance reached its peak (32.4 g/day) at the age of 17 years and remained consistent (31.6 g/day) in adulthood, but limited data were available (*n* = 4 studies, 112 patients).

There were more studies that expressed data as the actual amount consumed per kg of body weight (Figure 3 and Figure 5). However, the heterogeneity between study results was higher (usually more than 90%) than the data expressed as actual daily intakes. The results showed that the mean Phe tolerance per kg of body weight decreased from infancy until the beginning of adolescence (from 40 mg/kg/day at 6 months, *n* = 1 study, to 15.5 mg/kg/day at 6–10 years, *n* = 4 studies), whilst the decrease in natural protein tolerance per kg of body weight continued until adulthood (from 0.96 g/kg/day at 6 months, *n* = 2 studies, to 0.34 g/kg/day at 11–16 years; *n* = 4 studies). However, the number of patients with data was lower during adolescence.

Both Phe and natural protein tolerance gradually increased and reached ranges of 24–27 mg Phe/kg/day (*n* = 2 studies) and 0.47–0.60 g natural protein/kg/day (*n* = 5 studies) after 17 years of age. Alm et al. [27] reported higher intakes than other studies. Adherence was overall good/fair, and this study may have included mild patients, as suggested by the authors [27].

We also aimed to investigate gender differences in Phe/natural protein tolerance. However, only a limited number of studies reported data for gender groups (*n* = 4 studies for Phe intake and *n* = 5 studies for natural protein intake) (Supplementary Figures S1a–d and S2a–d), and a pooled analysis was only available for some age groups with a high degree of heterogeneity. Although a similar trend was observed for the change in Phe/natural protein tolerance between female and male patients with PKU, a comparison was not possible from the current literature due to limited and inconsistent data.

#### 3.3.2. Protein Equivalent from Protein Substitute Intake

Data regarding protein equivalent intake from protein substitutes were collected from 18 studies. Four of these studies reported data on the actual intake per day (g/day) [35,36,42,46], seven studies reported daily intakes per kg of body weight (g/kg/day) [33,34,38,40,43,44,51], and seven studies reported both [25,26,29,31,39,41,49].

A meta-analysis on the daily amount of protein equivalent intake (g/day) from a protein substitute was limited to the first 6 months of life (15.3 g/day) and for patients 6 years and older (ranged from 45.8 to 56.5 g/day). However, when data were expressed as grams per kg of body weight of protein equivalent obtained from a protein substitute, a meta-analysis was conducted for all age groups except two (4 years and >25 years). The mean protein equivalent intake was ≈2.0 g/kg/day during the first year of life, which decreased to 1.3 g/kg/day by 5 years of age. Although the mean intake showed an increase at the beginning of late childhood (1.6 g/kg/day; 6–10 years), it then declined to a level of 1.2 g/kg/day during adolescence. A further decrease was observed during adulthood, when protein equivalent from protein substitute intake contributed 0.5–0.6 g/kg/day (Figure 6 and Figure 7).

Available data for sex comparisons were also limited for protein equivalent intakes from protein substitute (Supplementary Figure S7a–d). A meta-analysis was only performed for some age groups; hence, it is not possible to reach any conclusions.

#### 3.3.3. Total Protein Intake

The results of a meta-analysis for the total protein intake are provided in Figure 8 and Figure 9. Four studies provided data on the actual amount of intake per day (g/day) [20,42,49,50], seven studies provided data on the total protein intake per kg of body weight (g/kg/day) [33,34,37,38,43,45,51], and seven studies reported both [25,26,31,39,41,44,49]. Data on total protein intakes were limited to between 7 months to 5 years of age (no studies for 3 years and 4 years of age, and *n* = 1 at 7–12 months, 1 year, and 2 years).

During the developmental stage, the mean daily total protein intake (g/day) almost doubled from birth until the end of 1 year (20.7 g/day at 6 months and 41.4 g/day at 1 year; mean difference = 20.7 g/day); this value then increased until 5 years of age (mean difference = 12 g/day) and slightly decreased between 6 and 10 years. There was a marked increase in the total protein intake (mean difference = +24 g/day) by adolescence, when the intake reached 77.5 g/day, and it plateaued during adulthood (81.7 g/day at >25 years) (Figure 8).

Similar to the Phe/natural protein intakes, there were more studies that expressed data as intake per kg of body weight (Figure 9). A pooled analysis could not be performed for the total protein intake over 25 years of age, as only one study reported the total protein intake of patients in this age group, in which the mean total protein intake (1.2 g/kg/day) was equal to the intake at 17–25 years. The results of the meta-analysis indicated that total protein intake consistently decreased by approximately 0.1–0.2 g/kg each year from birth until the end of the toddler years, decreasing from a mean intake of 2.7 g/kg/day at 6 months to a mean daily intake of 1.98 g/kg at 3 years. During early and late childhood (4–10 years), the total protein intake was consistent (≈1.8–1.9 g/kg/day) until the beginning of adolescence, when it decreased to a mean intake of 1.3 g/kg/day, similar to adulthood.

Due to limited data, sex comparisons could not be performed with accuracy for total protein intakes. Three studies reported data according to sex, which only enabled a pooled analysis of results at the age of 17–25 years (total protein g/day, g/kg/day) for males and females and between 11 years and 25 years (total protein g/day, g/kg/day) for males only, with high heterogeneity between the two studies included in the analysis (Supplementary Figure S3a–d).

### 3.4. Secondary Outcomes: Energy and Metabolic Control in Patients with PKU

#### 3.4.1. Energy Intake

A total of 11 studies reported energy intakes, which were reported as kcal/day (*n* = 6 studies) [19,20,29,31,42,50], kcal/kg/day (*n* = 1 study) [37], or both (*n* = 4 studies) [39,40,41,49]. 

A meta-analysis was only performed with data from two studies for late childhood (5–10 years: 1700–1798 kcal/day; 70 kcal/kg/day), adulthood (2109 kcal/day), and the first 6 months of age (680 kcal/day). There was a high degree of heterogeneity between studies, such that the mean difference between the reported intakes from two different studies for the same age group was more than 600 kcal (e.g., 2020 kcal/day vs. 1384 kcal/day at 5 years of age; *I*^2^ = 98%, *p* < 0.01) [20,49,50]. For the remaining age groups, the data shown in the forest plots were obtained from the mean energy intakes of a single study. Overall, it was not possible to estimate the trend in the total lifetime energy intake for patients with PKU (Supplementary Figures S4 and S5).

A pooled analysis could not be performed for energy intake to compare sex as there was only one study for each age group (Supplementary Figure S6a–d).

#### 3.4.2. Metabolic Control

The studies followed variable target therapeutic ranges for blood Phe levels. Thirteen studies [25,26,28,29,30,31,35,36,40,41,52,53,56,58] used similar targets to the European PKU guidelines [12]. Another three studies aimed for blood Phe levels <360 µmol/L, (<6 y), <480 (6–10 y), and <600 (>10 y) [32,33,34].

The remaining studies (*n* = 13) [18,19,20,27,37,42,44,46,47,51,54,55,57] with available data aimed for levels above the recommendations of the European PKU guidelines [12]. All the available data are presented in Supplementary Table S7. All patients participating in the studies aimed to have acceptable metabolic control according to the recommended targets of each centre, as defined by the inclusion criteria of this systematic review.

### 3.5. Quality Appraisal and Risk of Bias Assessment

We used two different tools to assess the quality of the studies included in the analysis (Table 2 and Table 3). Most of the cohort and cross-sectional studies were rated as “fair” (14 of 25 studies; 56%), whilst the remaining studies were rated either as “poor” (*n* = 8 studies; 32%) or “good” (*n* = 3 studies; 12%). The main issues were the lack of (i) a clear definition of the study population (i.e., who to recruit, from where, and from what time period), (ii) sample size justification/power analysis, (iii) an examination of different levels of exposure related to the outcome(s), (iv) blindness of study assessors, and (v) statistical measurement or an adjustment of confounding factors.

The controlled intervention studies had good quality as half of the 10 controlled studies were rated as “good” (*n* = 5 studies). Three of the studies (30%) had a “fair” quality rating, and two studies (20%) were qualified as “poor”. The main issues relating to fair/poor quality were the lack of; (i) the blindness of participants and assessors to the treatment group assignments and (ii) sample size justification/power analysis.

## 4. Discussion

In PKU, this is the first time that the change in Phe/natural protein tolerance over time has been systematically studied in patients treated with a Phe-restricted diet alone. Although several studies have reported natural protein, protein equivalent from protein substitute, and total protein intakes as part of their secondary outcomes, most studies were cross-sectional, and any specific focus on natural protein tolerance was scarce. The results of this meta-analysis show that natural protein (g/day) or Phe tolerance (mg/day) increased with age, particularly from the beginning of puberty, and reached their highest peak at the end of adolescence. The intakes of natural protein, expressed as g/kg/day, and Phe, expressed as mg/kg/day, decreased with age, with more stability observed after the age of 5 years. Total protein intakes within this meta-analysis were well above the U.S. Recommended Dietary Allowance (RDA) [59] and the safe level of protein intake reported by the FAO/WHO/UNU [60] for a healthy population. The mean intake was 2 times higher than the RDA/safe level from birth until adolescence and 1.5 times higher during adolescence and adulthood, values which are above the guidelines for total protein intake for patients with PKU (≈120–140% of RDA or FAO/WHO/UNU safe levels) [12,61]. This is in line with the European PKU guidelines [12] that recommend that total protein intake should supply the age-related safe levels of protein intake (FAO/WHO/UNU), with an additional 40% from L-amino acid supplements to account for the inefficient absorption of synthetic amino acids and their impact on blood Phe control.

Theoretically, Phe/natural protein restrictions should be calculated according to patient weight, supporting our finding that Phe/natural protein intake (when expressed as mg/day or g/day, respectively) increased throughout childhood and adolescence. However, Phe/natural protein intake is also titrated with blood Phe control, and patients with classical PKU tolerate less than those with milder phenotypes. Actual Phe/natural protein tolerance may also be higher than the amounts prescribed by health professionals as their priority is to maintain blood Phe within a target therapeutic range. Accurate data on Phe/protein intake are commonly difficult to establish. The few studies that have explored Phe/natural protein intake in adulthood have shown that patients commonly tolerate more than the amounts prescribed during childhood years [62,63], although many adult patients describe their daily Phe/natural protein allowance as the same amount allocated during childhood. Pinto et al. [29] showed that patients with milder PKU tolerated more Phe/natural protein than expected prior to sapropterin responsive testing. Health professionals may not actively re-examine protein tolerance with increasing age. Furthermore, patients or caregivers may be less careful at measuring Phe/natural protein intake with time [64,65]. MacDonald et al. [64] showed that the weighing of Phe exchanges became less common with the increasing age of children, with parents only estimating portion sizes by “eye” from Phe-containing foods after the early childhood years. It is suggested that some children probably eat more protein than prescribed, even though they achieve acceptable blood Phe control [64]. Some teenagers who self-manage their dietary treatment may have little knowledge about the protein content of foods, and it has also been shown that both adults with PKU and caregivers have difficulties in interpreting food protein content from food labels and consequently only approximate the amounts of protein contained in food [66]. Some patients may also feel they cannot disclose their actual Phe/protein intake in case of disapproval from health professionals.

Some of the changes observed in Phe/protein tolerance may reflect different blood Phe therapeutic target ranges, which varied between countries, patient age, and clinical state (pre-conception). Theoretically, a higher upper blood Phe target range should lead to an increased Phe/protein tolerance. In our systematic review, we included several studies over a 40-year duration from 15 different countries. Over time, treatment guidelines for blood Phe targets have increased in stringency. There were also wide differences in protein tolerance between patients with classical and milder PKU, but many studies did not clearly identify patient phenotype, particularly the studies reported over 10 years ago. Generally, patients from Southern Europe [4] tend to have milder PKU, and interestingly, most of the adult studies included in this systematic review were from Portugal, Spain, and Italy. In contrast, many of the paediatric studies included were from the UK, Germany, the Netherlands, the USA, and Australia, and in most of these countries, classical PKU is common. Therefore, Phe tolerance may have been under or overestimated in children and adults with PKU, respectively.

In the first two years of life, our systematic review showed that the mean Phe intake was between 28 to 40 mg/kg/day, decreasing with age. At 0–6 months, it was 40 mg/kg/day, 32 mg/kg/day at 12 months, and 28 mg/kg/day at 2 years. Interestingly Holt and Snyderman [67], using growth and nitrogen balance in 27 infants with PKU, estimated the Phe requirement to be 55–90 mg/kg/day (mean 70 mg/kg/day) at 2 months of age and 25 to 80 mg/kg/day (mean 35 mg/kg/day) at 12 months of age. In young children with PKU, there is evidence of a positive correlation between natural protein intake and head circumference (but not height) within the first three years of life [43]. There is also a concern that young children with PKU may be at risk of Phe deficiency if they consistently refuse dietary Phe sources associated with food neophobia or have avoidant or restrictive food intake, which have been frequently described in the literature [68,69], in addition to the limitations of the therapeutic diet [14]. Phe intake may also be compromised during frequent childhood infections. Although it is not advocated that Phe intake is lowered with infections, appetite may be reduced, thereby limiting Phe intake. Furthermore, energy intake may be insufficient, possibly associated with restrictive eating behaviours. Additionally, nitrogen balance is sensitive to changes in energy intake over a range of protein intakes from low to high [60]. Increasing energy intake will enhance protein synthesis and reduce amino acid oxidation [70].

This systematic review indicated that from the age of 3 years, natural protein tolerance was consistently <0.5 g/kg/day protein, although in Australia, Evans et al. [38] showed in a small group of children with PKU that a natural protein intake >0.5 g/kg/day was associated with improved body composition. There was a strong correlation with a higher % of fat mass and a natural protein intake of <0.48 g/kg/day but no correlation between % fat mass and a natural protein intake of ≥0.5 g/kg/day [38]. Also, we observed in children aged 3 to 10 years that Phe intake was mainly below 20 mg/kg/day. This is similar to the results found by Courtney-Martin et al. [4], who estimated Phe requirements in five children with classical PKU (age 5–13 years) via indicator amino acid oxidation, using L-[1-13C] lysine as the indicator. They concluded that Phe requirements were 14 mg/kg/day, but the safe population intake was (accounting for an upper 95% confidence of the mean) 19.5 mg/kg/day. They suggested that the maximal Phe intake for children with PKU should be no higher than 20 mg/kg/day [4]. In our systematic review, one study from Sweden, conducted in the 1980s, indicated a higher Phe tolerance in children. The classification of PKU was not given, and the methods of documenting and analyzing Phe intake were not described. Nine of twenty-three children tolerated a normal diet while maintaining Phe levels between 250 and 720 µmol/L. Treatment was commenced when the Phe level was above 720 µmol/L during the neonatal period. Although their results demonstrate the importance of periodically challenging patients with extra dietary Phe, they are atypical of other studies [27].

It was also shown in 10 healthy male adults that the mean requirement for Phe, as determined via Phe flux and oxidation, is 10 mg/kg/day in the presence of excess tyrosine (40 mg/kg/day) and adequate total amino acids (1 g/kg/day) [71]. From our systematic review, the limited data available in adults with PKU suggests that the Phe intake exceeds the safe protein intakes of the FAO/WHO/UNU in healthy adults [60]. Overall, there is a lack of knowledge about exact Phe/protein tolerance in adult patients who aim to achieve the European or USA blood Phe treatment guidelines except during maternal PKU [12,61]. Phe/natural protein requirements particularly increase in the last trimester of pregnancy, but this is not a true reflection of tolerance throughout the adult lifespan. Unfortunately, in this systematic review, there were insufficient data to compare protein tolerance between men and women, and little is known about the impact of menstruation and menopause on Phe/protein tolerance. Although several metabolic alterations have been described, no specific change in protein requirements are recommended for the general population. Also, in the general population, it is unclear if protein requirements are higher in elderly. The utilization and digestibility of protein decreases with age [72], and sarcopenia is common with ageing [73], but there is no information about the impact of ageing on natural protein tolerance in PKU [74] or any data on natural protein intake.

In general, the total amount of protein equivalent (g/day) obtained from a protein substitute increased until the age of 6–10 years and then remained stable, although it slightly decreased at 17–25 years. Clinics in Northern Europe prescribe a higher protein equivalent intake from protein substitutes, whereas Central and Southern Europe prescribe lower amounts [75]. It is established that adherence with consuming protein substitutes declines in adolescence, and this may explain the slight decrease in protein equivalent intake [76]. It may also have a negative effect on the final height achieved [77]. It is well established that Phe-free amino acids from protein substitutes lower blood Phe levels by inhibiting protein catabolism and stimulating protein synthesis, and although the dose of protein substitute remains controversial in PKU, the protein substitute is an integral part of nutritional care [18,46,78,79]. It has been described that protein substitutes are not well utilized due to oxidation; therefore, the European PKU guidelines [12] suggests an additional 40% over the age-related safe levels of protein intake (FAO/WHO/UNU) [60]. The majority of patients were prescribed amino acid supplements, but it is not known how protein substitutes containing different sources of protein equivalent, such as casein glycomacropeptide and slow-release amino acids, may affect Phe tolerance.

Total protein intake (including protein equivalent from a protein substitute) may also influence natural protein tolerance. Kindt et al. [45] reported that 5- to 15-month-old children with PKU had higher Phe requirements when protein intake (including natural and protein equivalent from a protein substitute) was provided in accordance with the 1980 USA Recommended Dietary Allowance (RDA) for age than when provided in accordance with the lower 1985 FAO/WHO/UNU protein requirements [45]. In 25 infants with PKU, Acosta et al. [80] reported that a mean total protein intake 24% greater than the RDA was associated with better Phe tolerance (38% higher) than when the mean protein intake was only 9% greater than the RDA [80]. In this systematic review, total protein intakes were found to be higher than the recommendations provided in USA and European PKU guidelines [12,61], but similar to what was prescribed in Northern and Eastern Europe by Aguiar et al. [75].

In PKU, there are some important physiological aspects for which there is limited information about Phe/protein requirements, particularly the impact of high-intensity sports. This has the potential to both improve or even impair Phe tolerance. Sport performance that is optimally nutritionally managed may enhance tolerance associated with anabolism, but when nutrition is suboptimal, it may lead to catabolism and a lowering of Phe tolerance. Limited research has shown that blood Phe levels do not change with acute exercise [81,82], but patients had lower branch-chain amino acid concentrations when fasting when compared with controls. Moreover, patients with poor metabolic control (higher Phe levels) had lower percentages of prescribed VO_2_ (the maximum rate of oxygen (O_2_) that can be used during exercise) compared to controls and well-controlled patients [82]. There is a need to assess protein requirements and the effects of different types of exercise on Phe/natural protein tolerance in the future. It is also important to study how protein intake and metabolic control may help improve performance.

Adult PKU services are increasing exponentially in all countries, with patients transitioning from paediatric services. Our systematic review shows that there are inadequate data describing Phe tolerance throughout the life cycle, especially during different stages of adulthood. Several physiological conditions of adult life as well as environmental factors may impact Phe tolerance and the ability to achieve good metabolic control. It is crucial to design and perform future studies examining Phe tolerance with protein requirements through adulthood, considering patient phenotype, sex, treatment regimen, and nutritional status. This will help better identify patient needs to help recommend generalised dietary and individual practices in the management of adults with PKU.

It is important that Phe tolerance of all patients (on dietary and or drug treatment) is assessed annually. This should be conducted systematically by challenging the patient with extra dietary Phe. Phe deficiency should be avoided. We consider that in all future clinical PKU studies, it should be recommended that Phe tolerance (prescribed and actual intake) should be collected as part of core outcome measures, irrespective of the primary outcome being assessed. This should include patients on dietary treatment only and those using different pharmaceutical treatments. This was previously suggested by a Canadian working group [83] and will help to standardise the data that are collected in order to compare research findings.

### Limitations

This systematic review with a meta-analysis has several limitations. Some articles (*n* = 56) were excluded during the screening process due to insufficient information about dietary intake. The studies included had different research questions, and the information obtained regarding Phe/natural protein, protein substitutes, and total protein was a secondary outcome. Therefore, even though the quality of the intervention studies may have been good, the data collected regarding nutritional intake may not have been methodically collected or complete. Any self-reports of Phe intake may have under-reported intake if the food intake was not weighed or accurately measured. The number of studies included in the meta-analysis was suboptimal, with limited patient data for each of the age groups defined. There was no data for the ageing PKU population. We were unable to perform a meta-analysis according to PKU severity (i.e., comparison of Phe tolerance in mild vs. classical PKU) due to low patient numbers and incomplete patient phenotype data, and this may have influenced our results, particularly in adulthood. In some studies, patients may only have followed partial diet therapy; however, we excluded studies when there was evidence of poor patient adherence. Some countries had limited access to special low-protein foods, potentially limiting energy intake, which would have negatively impacted Phe tolerance in some reports. No information was reported on the quality of the natural protein consumed.

Dietary Phe intakes were reported differently according to each country’s system for allocating Phe. Some PKU centres recommended that patients calculate every milligram of Phe that was consumed, whereas others used a Phe exchange system [84] and permitted fruits and vegetables containing Phe ≤ 75 mg/100 g or foods containing protein ≤ 0.5 g without measurement. It is likely that this latter practice led to an underestimation of Phe/protein intake as it has been established that Phe/protein intake from unmeasured sources may increase intake by around 100% [57]. Some studies only included information about prescribed natural protein intake, and this was excluded in our meta-analysis. The quality of the interventional studies was usually “good”, but most studies were cohort and cross-sectional studies, and the quality was mainly fair or poor. This also contributed to the high level of heterogeneity (usually between 80 and 96%) of our results, and generally, studies only included small sample sizes (≤30 patients in 49% of studies), which may have had an important impact on the quality of the results. Most random effects models could not be used to perform male/female comparisons as well as for some age groups due to a lack of data. Heterogeneity was associated with differences in phenotypic distribution between countries and variation in treatment practices, with target blood Phe levels changing over time. It was common for data to be reported about Phe/protein tolerance in patients ≤16 years but after late adolescence, data on natural protein intake were scarce and had only been reported by a few authors [25,26,29,42,51]. This may be explained by the discontinuation of dietary treatment in the late adolescent years or the loss of follow-up of adult patients. In addition, patients who remained on dietary treatment after transitioning to adult services take more responsibility for their own treatment and may not routinely report their Phe/protein intake to health professionals.

## 5. Conclusions

This meta-analysis suggests that Phe/natural protein tolerance (expressed as g or mg per day) increases with age, particularly from the beginning of puberty, and reaches a peak at the end of adolescence. There also appeared to be an increase in natural protein intake when target blood Phe levels were relaxed in teenage and adult years. However, there is considerable uncertainty in these conclusions given the high risk of bias in many studies and the high heterogeneity between studies due to differences in sample size, the severity of PKU, and therapeutic target levels for blood Phe control. More data is needed to establish how natural protein tolerance changes throughout the lifespan and how it is affected by different life cycle stages in both males and females with PKU. Additional carefully conducted studies are necessary to examine Phe requirements in adulthood, ageing, and physical activity in order to deliver appropriate individualised patient care.

## Figures and Tables

**Table 2:** Quality appraisal and risk of bias of observational cohort and cross-sectional studies.

NIH Quality Assessment Tool for Observational Cohort and Cross-Sectional Studies	Research Question	Study Population	Participant Rate at Least 50% from Eligible	Participants from Same/Similar Population	Sample Size Justification	Exposure(s) Measured Prior to the Outcome(s)	Sufficient Time Frame	Examination of Different Levels of Exposure as Related to the Outcome	Definition and Validation of the Exposure Measures	Exposure(s) Assessed More than Once	Definition of Outcome Measures	Blinded Assessors	Loss to Follow-Up after Baseline 20% or Less	Statistical Measure and Adjustment of Key Confounding Variables	Overall Rating
Acosta et al., 1998 [31]	Y	N	?	Y	N	Y	Y	Y	?	Y	Y	N	Y	Y	**Good**
Aldámiz-Echevarría et al., 2013 [33]	Y	N	?	Y	N	Y	Y	Y	Y	Y	Y	N	?	N	**Fair**
Aldámiz-Echevarría et al., 2014 [32]	Y	Y	?	Y	N	Y	Y	N	Y	Y	Y	N	?	N	**Fair**
Aldámiz-Echevarría et al., 2015 [34]	Y	N	?	Y	N	Y	Y	N	Y	Y	Y	N	Y	N	**Fair**
Alm et al., 1986 [27]	Y	Y	?	N	N	Y	Y	NA	?	Y	Y	N	Y	N	**Poor**
Dobbelaere et al., 2003 [37]	Y	N	?	Y	N	N	N	N	Y	N	Y	N	NA	N	**Poor**
Evans et al., 2017 [38]	Y	Y	?	Y	N	Y	Y	N	Y	Y	Y	N	NA	N	**Fair**
Evans et al., 2018 [53]	Y	Y	?	Y	N	Y	Y	Y	Y	Y	Y	N	Y	N	**Good**
Evans et al., 2019 [39]	Y	N	?	Y	Y	Y	Y	Y	Y	Y	Y	N	Y	Y	**Fair**
Evers et al., 2018 [28]	Y	Y	?	Y	N	Y	Y	N	Y	Y	Y	N	Y	N	**Fair**
Green et al., 2019 [42]	Y	N	?	Y	N	N	N	Y	Y	N	Y	N	NA	N	**Poor**
Hoeksma et al., 2005 [43]	Y	Y	?	Y	N	Y	Y	N	Y	Y	Y	N	Y	Y	**Good**
Huemer et al., 2007 [44]	Y	N	?	Y	N	Y	Y	N	Y	Y	Y	N	Y	N	**Fair**
Kindt et al., 1983 [45]	Y	Y	Y	Y	N	Y	Y	Y	N	Y	N	N	Y	N	**Poor**
MacDonald et al., 1996 [57]	Y	N	?	?	N	Y	Y	Y	N	Y	Y	N	Y	N	**Fair**
Pinto et al., 2019 [29]	Y	N	?	Y	N	Y	Y	N	Y	Y	Y	N	Y	N	**Fair**
Ponzone et al., 2008 [47]	Y	N	?	Y	N	Y	Y	Y	Y	Y	Y	N	Y	Y	**Fair**
Rocha et al., 2012 & 2013 [25,26]	Y	Y	?	Y	N	N	N	N	Y	N	Y	N	NA	N	**Poor**
Rohde et al., 2014b [49]	Y	N	?	N	N	Y	NA	Y	N	N	Y	N	NA	N	**Poor**
Rohde et al., 2015 [48]	Y	N	?	Y	N	N	N	Y	Y	N	Y	N	?	N	**Poor**
Schulpis et al., 2013 [50]	Y	N	?	Y	N	Y	N	Y	Y	Y	Y	N	NA	Y	**Fair**
Stockler-Ipsiroglu [58]	Y	N	?	Y	N	Y	Y	Y	Y	Y	Y	N	Y	N	**Fair**
Thiele et al., 2017 [51]	Y	Y	Y	Y	N	Y	Y	N	N	Y	Y	N	N	Y	**Fair**
van Spronsen et al., 2009 [55]	Y	Y	Y	Y	N	Y	Y	N	Y	Y	Y	N	Y	N	**Fair**
Wendel et al., 1990 [56]	Y	Y	Y	Y	N	Y	Y	N	Y	Y	N	N	N	N	**Poor**

Each item was rated as low-risk (“yes” = Y, green), unclear (“cannot determine/not reported” = ?, yellow), or high-risk (“no” = N, red) for the type of bias. NA, not applicable.

**Table 3:** Quality appraisal and risk of bias of controlled intervention studies.

NIH Quality Assessment of Controlled Intervention Studies	Study Described as a RCT	Adequate Randomization Methods	Concealed Treatment Allocation	Blinded Participants	Blinded Assessors	No Group Differences at Baseline	Low Attrition/Loss of Participants	No Group Differences in Attrition	High Adherence Rates	Avoidance of Interventions Other than the Intended Treatment	Clear, Valid and Reliable Outcome Measures	Sample Size Justification	Prespecified Outcomes and Subgroup Analysis	Use of an Intention-to-Treat Analysis	Overall Ranking
Daly et al., 2019 [36]	Y	Y	Y	N	N	Y	Y	Y	Y	Y	Y	Y	Y	Y	**Good**
Giovaninni et al., 2014 [40]	Y	Y	Y	N	N	Y	Y	Y	Y	Y	Y	Y	Y	Y	**Good**
Daly et al., 2017 [35]	N	NA	N	N	N	Y	Y	Y	?	Y	Y	N	Y	Y	**Poor**
Gökmen-Özel et al., 2011 [41]	Y	Y	Y	N	N	Y	?	?	?	Y	Y	Y	Y	Y	**Fair**
MacDonald et al., 2006 [46]	Y	Y	Y	N	N	Y	Y	Y	?	Y	Y	Y	Y	Y	**Good**
MacDonald et al., 2003 [18]	Y	Y	Y	N	N	Y	Y	Y	?	Y	N	N	Y	Y	**Fair**
Rohde et al., 2012 & 2014a [19,20]	Y	?	?	N	N	Y	Y	Y	?	Y	Y	N	Y	Y	**Fair**
Trefz et al., 2009 [52]	Y	Y	Y	Y	Y	Y	Y	Y	?	Y	Y	Y	Y	Y	**Good**
Sweeney et al., 2012 [30]	Y	Y	Y	N	N	Y	Y	Y	?	Y	Y	?	Y	Y	**Good**
Ferguson et al., 1996 [54]	N	NA	N	N	N	N	N	N	Y	Y	Y	?	Y	Y	**Poor**

Each item was rated as low-risk (“yes” = Y, green), unclear (“cannot determine/not reported” = ?, yellow), or high-risk (“no” = N, red) for the type of bias. NA, not applicable.

## Data Availability

Data are contained within the article or Supplementary Materials.

## Supplementary Materials

The following supporting information can be downloaded at: https://www.mdpi.com/article/10.3390/nu15163506/s1, The following are available online at: Table S1. Actual dietary intakes of PKU patients in early childhood (0–3 years of age); Table S2. Actual dietary intakes of PKU patients in late childhood (4–10 years of age); Table S3. Actual dietary intakes of PKU patients during adolescence (11–18 years of age); Table S4. Actual dietary intakes of adult PKU patients (>18 years of age); Table S5. Dietary prescriptions of the patients with PKU in the included studies; Table S6. Description of dietary treatment; Table S7. Data on the metabolic control of patients with PKU in the included studies; Figure S1. Phenylalanine intakes (mg/day) of female (a) and male (b) participants in the included studies; Figure S2. Natural protein intakes (g/day) of female (a) and male (b) participants in the included studies. Natural protein intakes per kg body weight (g/kg/day) of female (c) and male (d) participants in the included studies; Figure S3. Total protein intakes (g/day) of female (a) and male (b) participants in the included studies; Total protein intakes per kg body weight (g/kg/day) of female (c) and male (d) participants in the included studies; Figures S4 and S5. Energy intakes per day (kcal/day; left) and per kg of body weight (kcal/kg/day; right) of participants in the included studies; Figure S6. Energy intakes per day (kcal/day; (a) female, (b) male) and per kg of body weight (kcal/kg/day; (c) female, (d) male) of participants in the included studies; Figure S7. Protein equivalent from protein substitute intakes per day (g/day) of female (a) and male (b) participants in the included studies. Protein equivalent from protein substitute intakes per day (g/kg/day) of female (c) and male (d) participants in the included studies.
