# Supplementary material for: Phenylalanine Tolerance over Time in Phenylketonuria: A Systematic Review and Meta-Analysis
Source: Nutrients. 2023 Aug 8;15(16):3506. doi: 10.3390/nu15163506 (PMC10458574; doi:10.3390/nu15163506)
Supplement: Supplementary file 1 [file nutrients-15-03506-s001.zip › Supplementary Figures S1-S7_06072023.pdf]

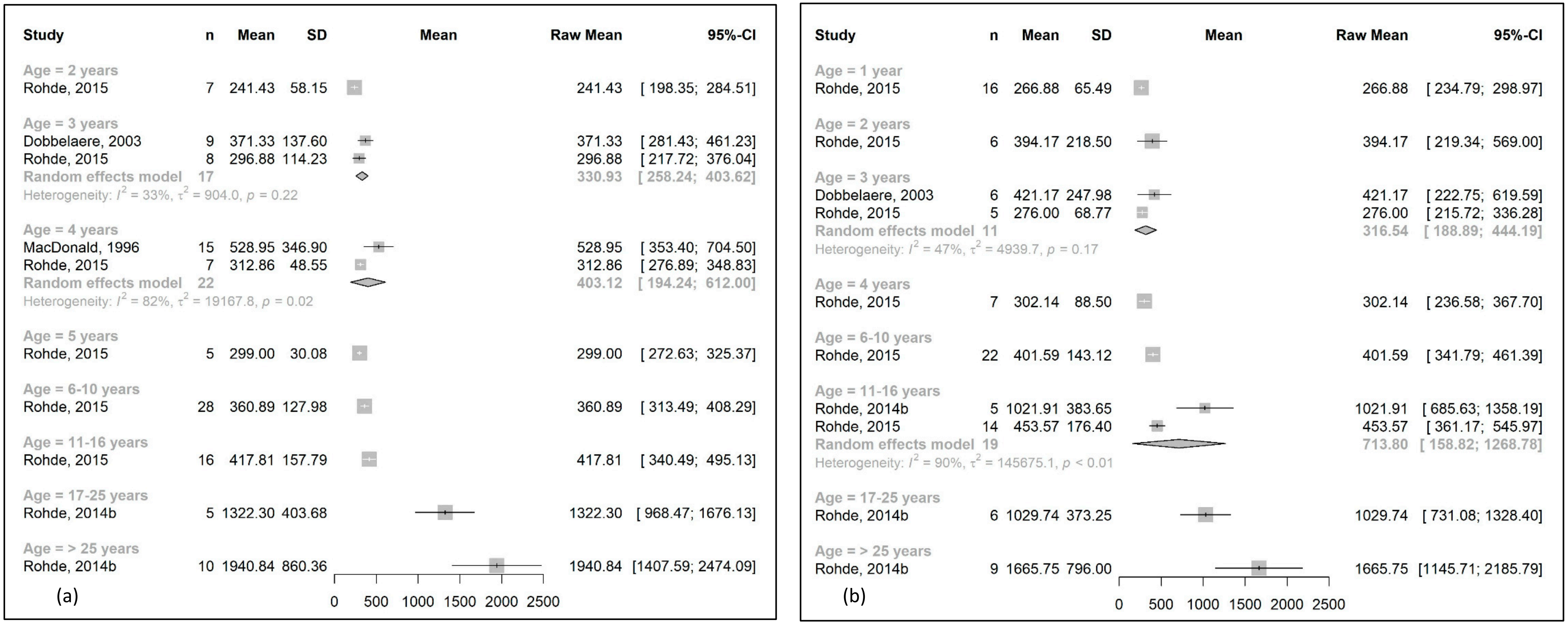


**Supplementary Figure S1.** Phenylalanine intakes (mg/day) of female (**a**) and male (**b**) participants in the included studies.

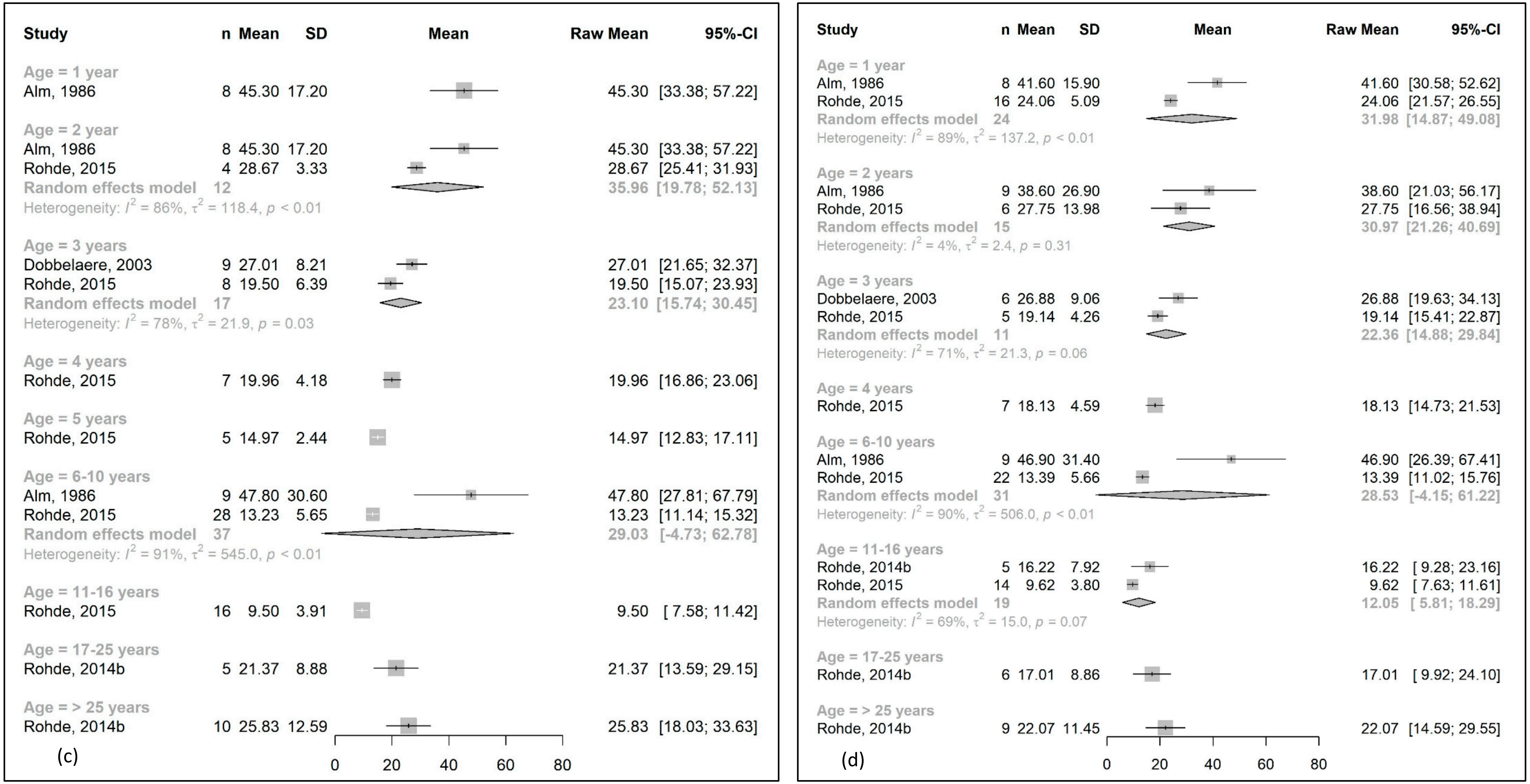


**Supplementary Figure S1.** Phenylalanine intakes per kg body weight (mg/kg/day) of female (**c**) and male (**d**) participants in the included studies.

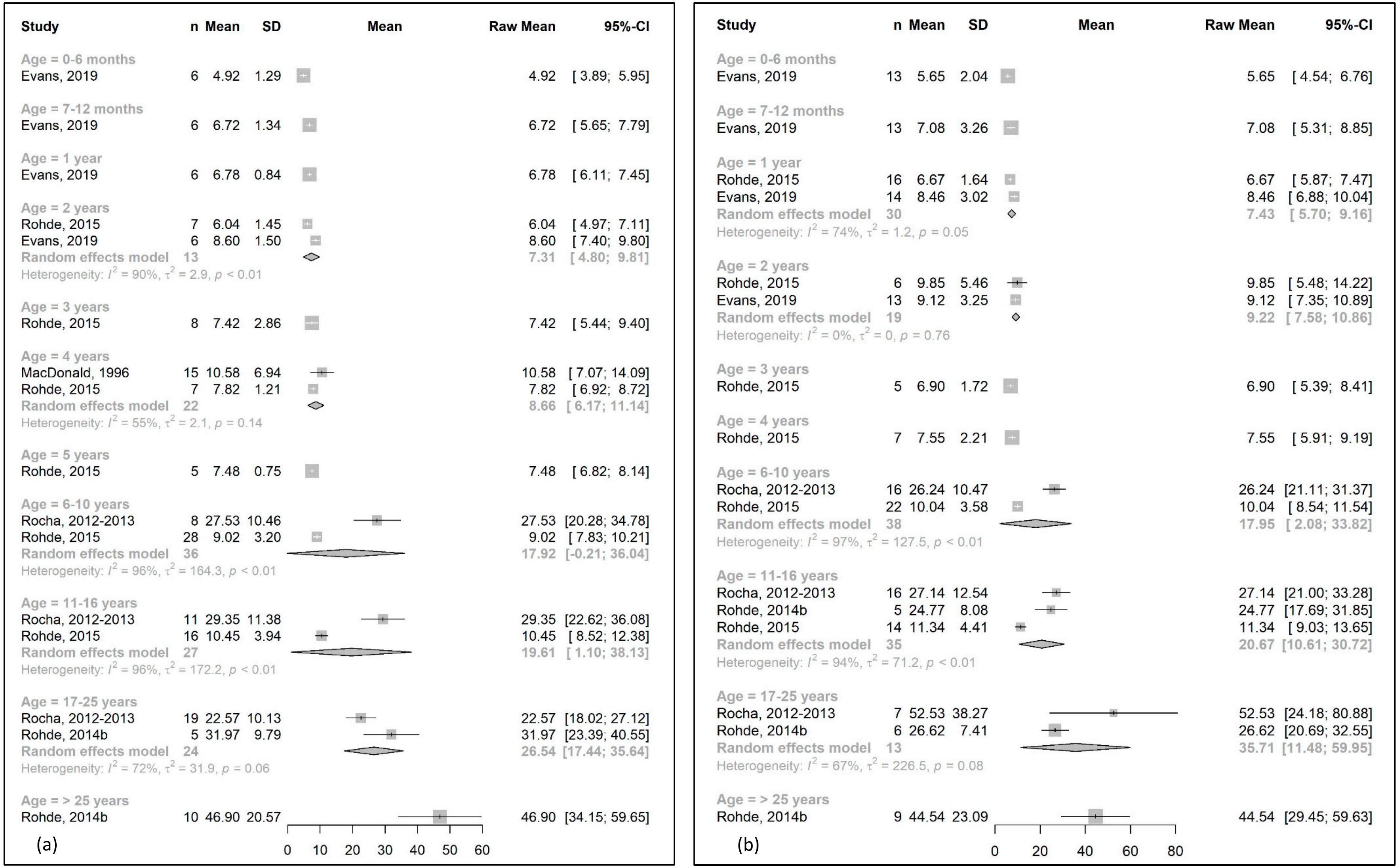


**Supplementary Figure S2.** Natural protein intakes (g/day) of female (**a**) and male (**b**) participants in the included studies.

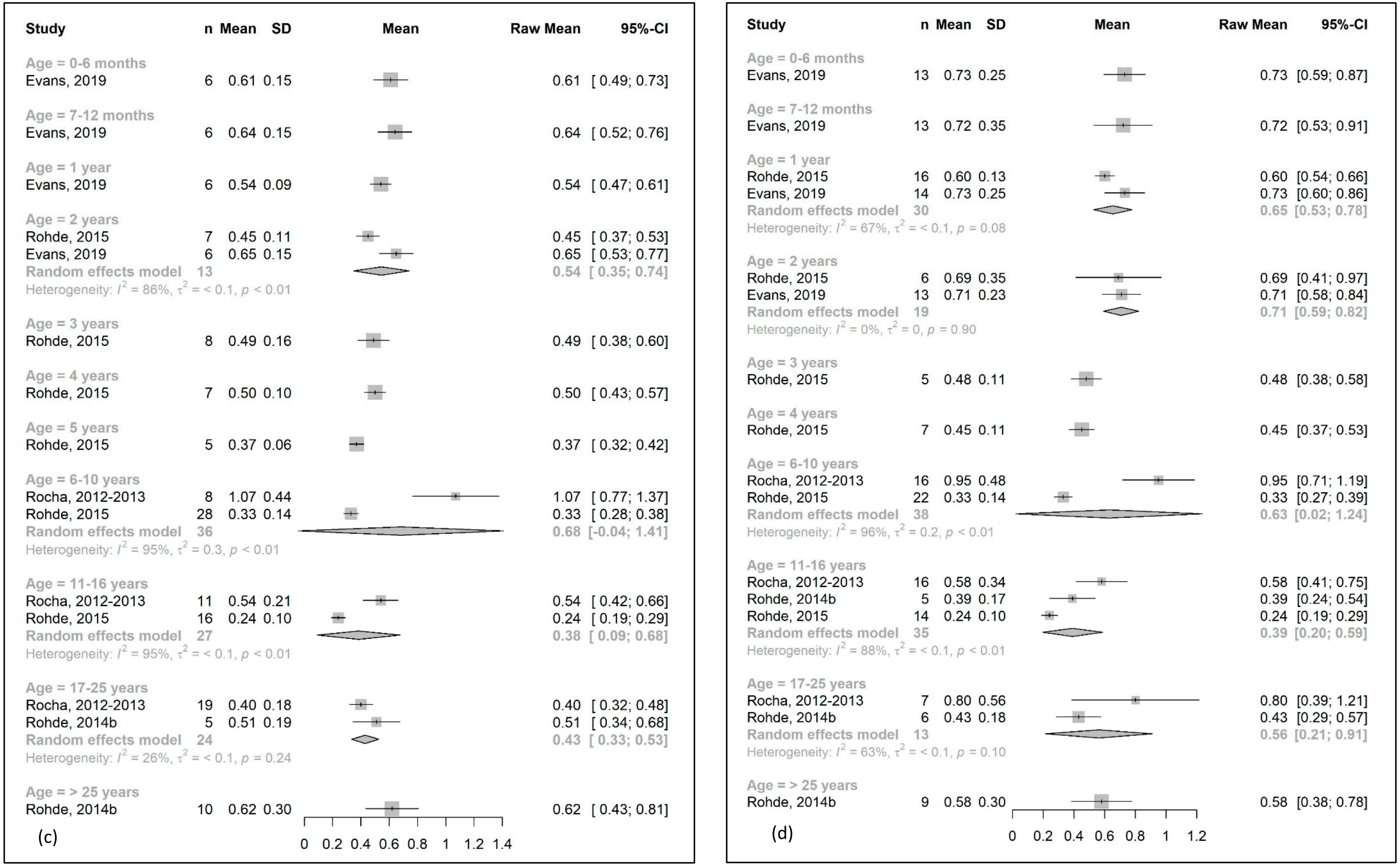


**Supplementary Figure S2.** Natural protein intakes per kg body weight (g/kg/day) of female (**c**) and male (**d**) participants in the included studies.

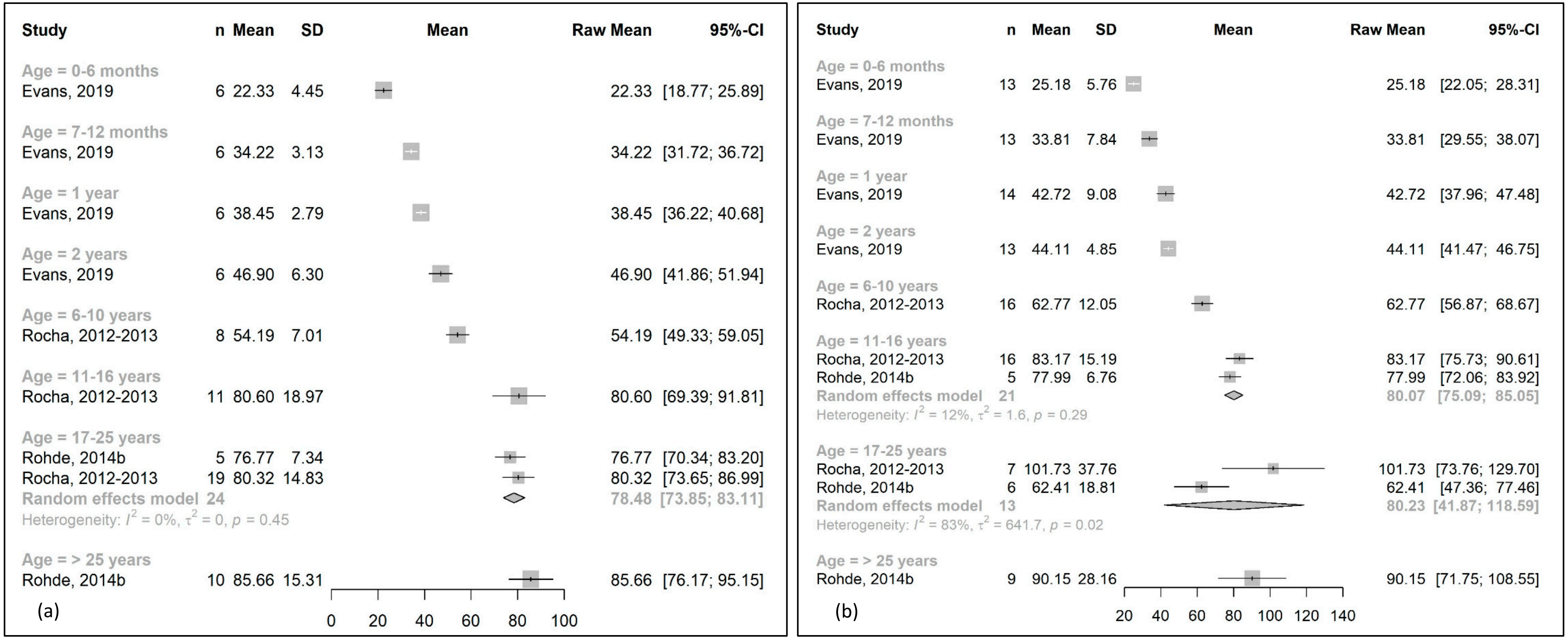


**Supplementary Figure S3.** Total protein intakes (g/day) of female (**a**) and male (**b**) participants in the included studies.

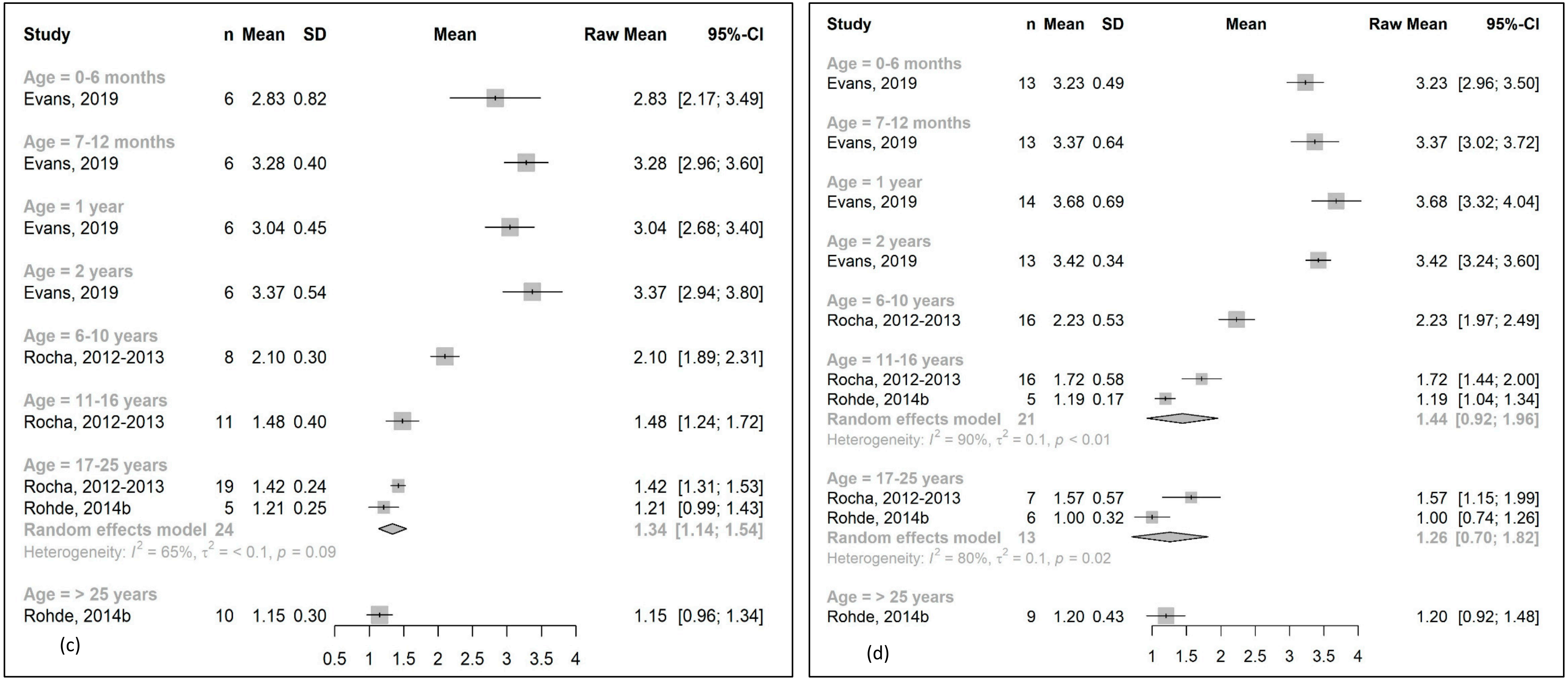


**Supplementary Figure S3.** Total protein intakes per kg body weight (g/kg/day) of female (**c**) and male (**d**) participants in the included studies.

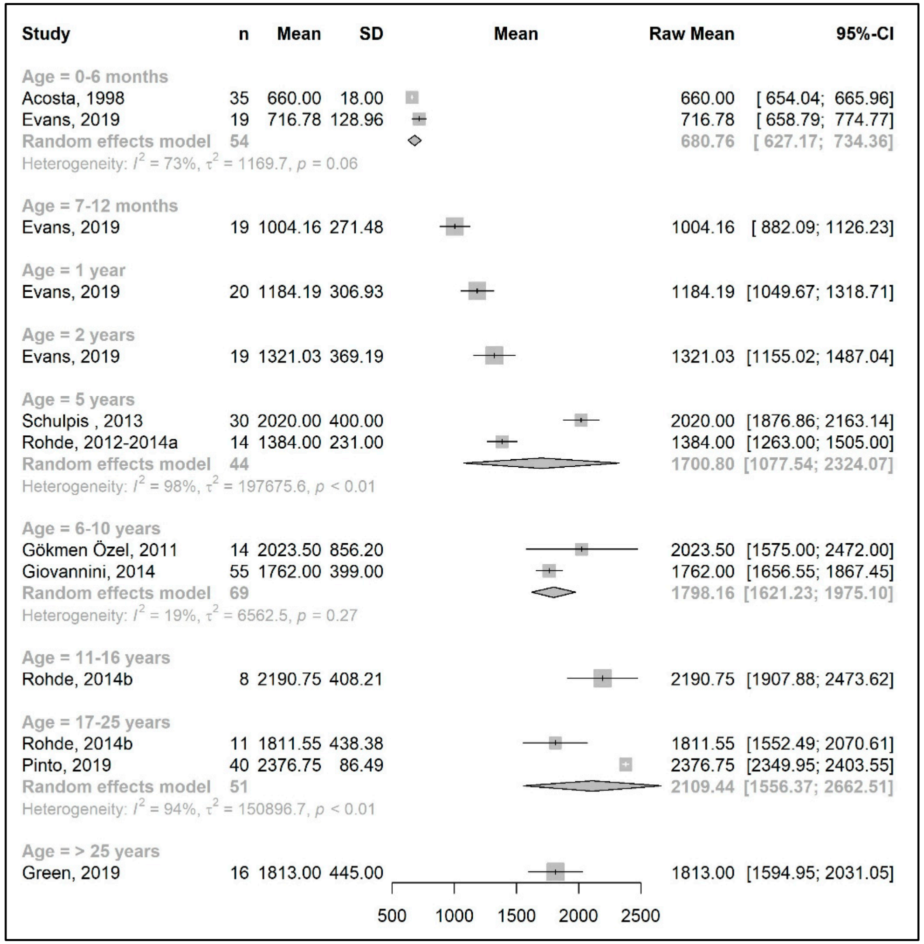


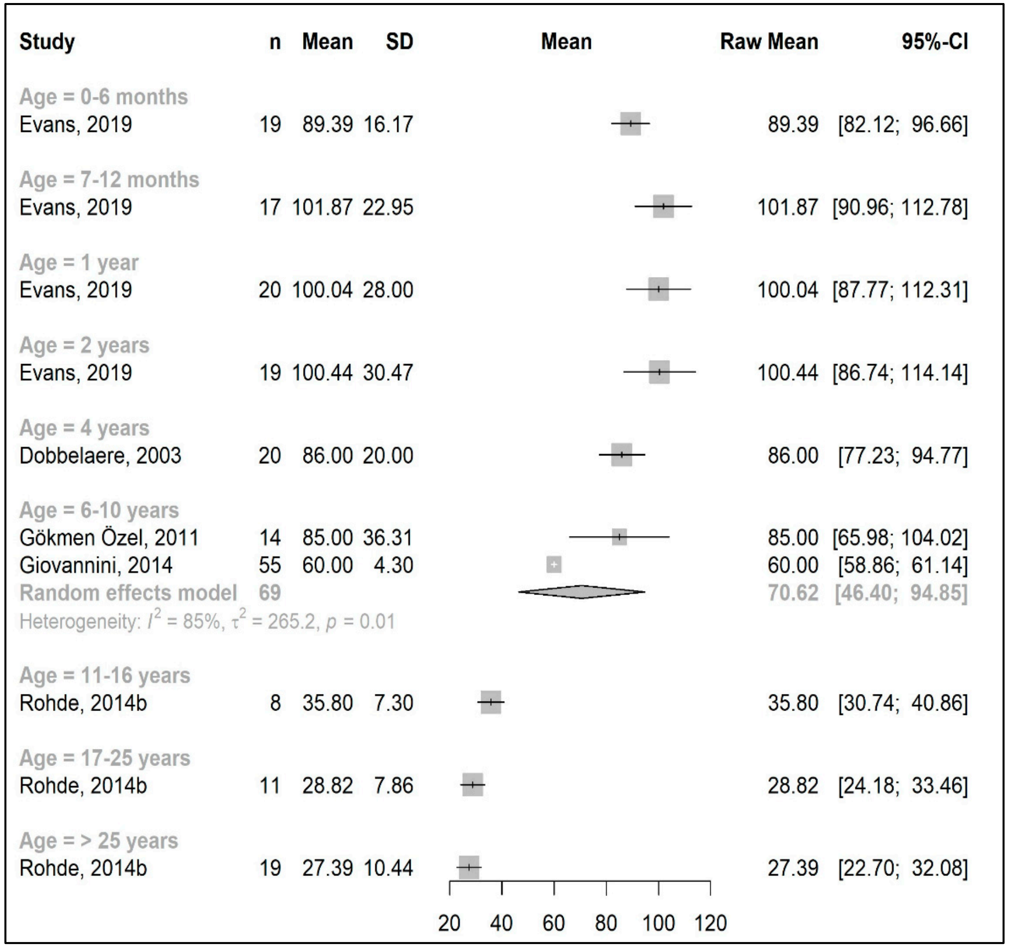


**Supplementary Figures S4 and S5.** Energy intakes per day (kcal/day; **left**) and per kg body weight (kcal/kg/day; **right**) of participants in the included studies.

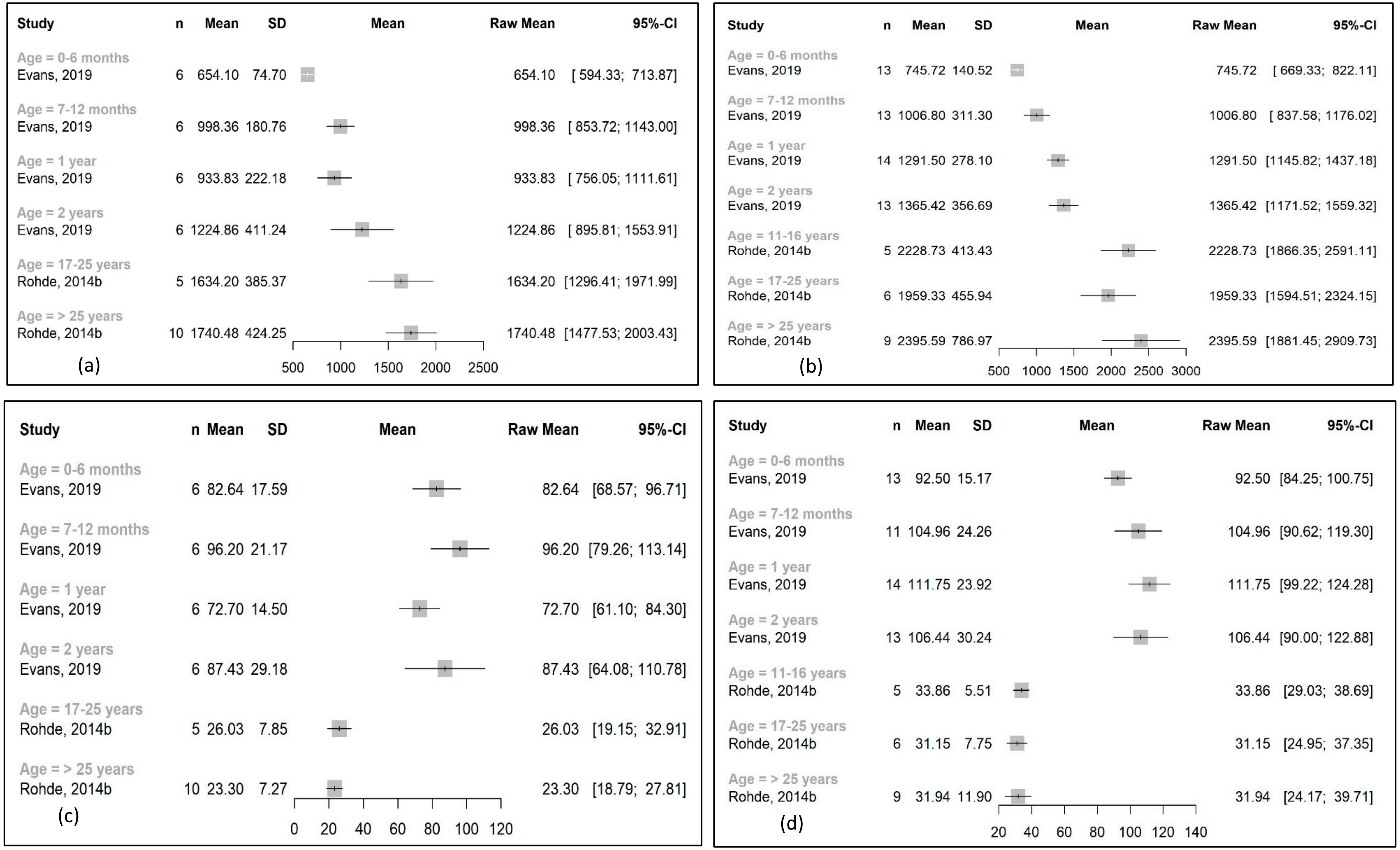


**Supplementary Figure S6.** Energy intakes per day (kcal/day; (**a**) female, (**b**) male) and per kg of body weight (kcal/kg/day; (**c**) female, (**d**) male) of participants in the included studies..

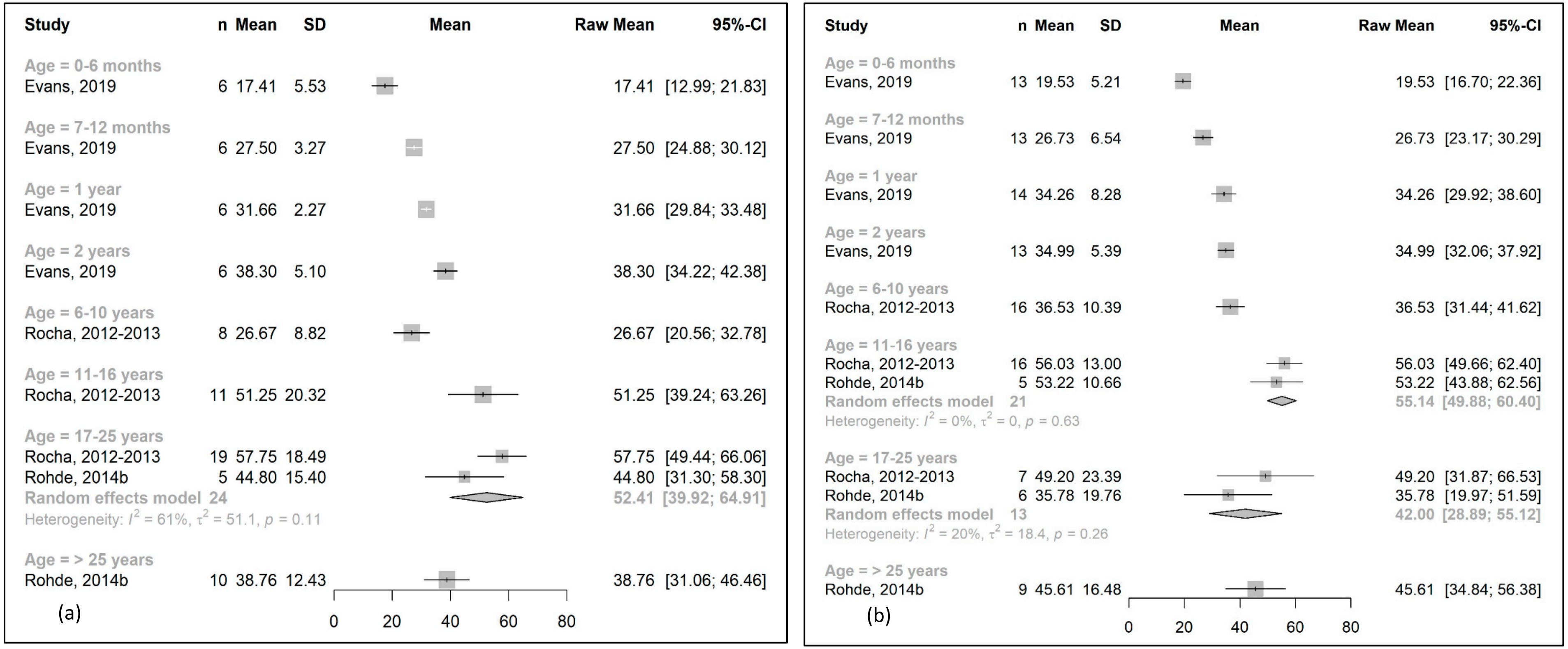


**Supplementary Figures 7a-7b.** Protein equivalent from protein substitute intakes per day (g/day) of female (**a**) and male (**b**) participants in the included studies.

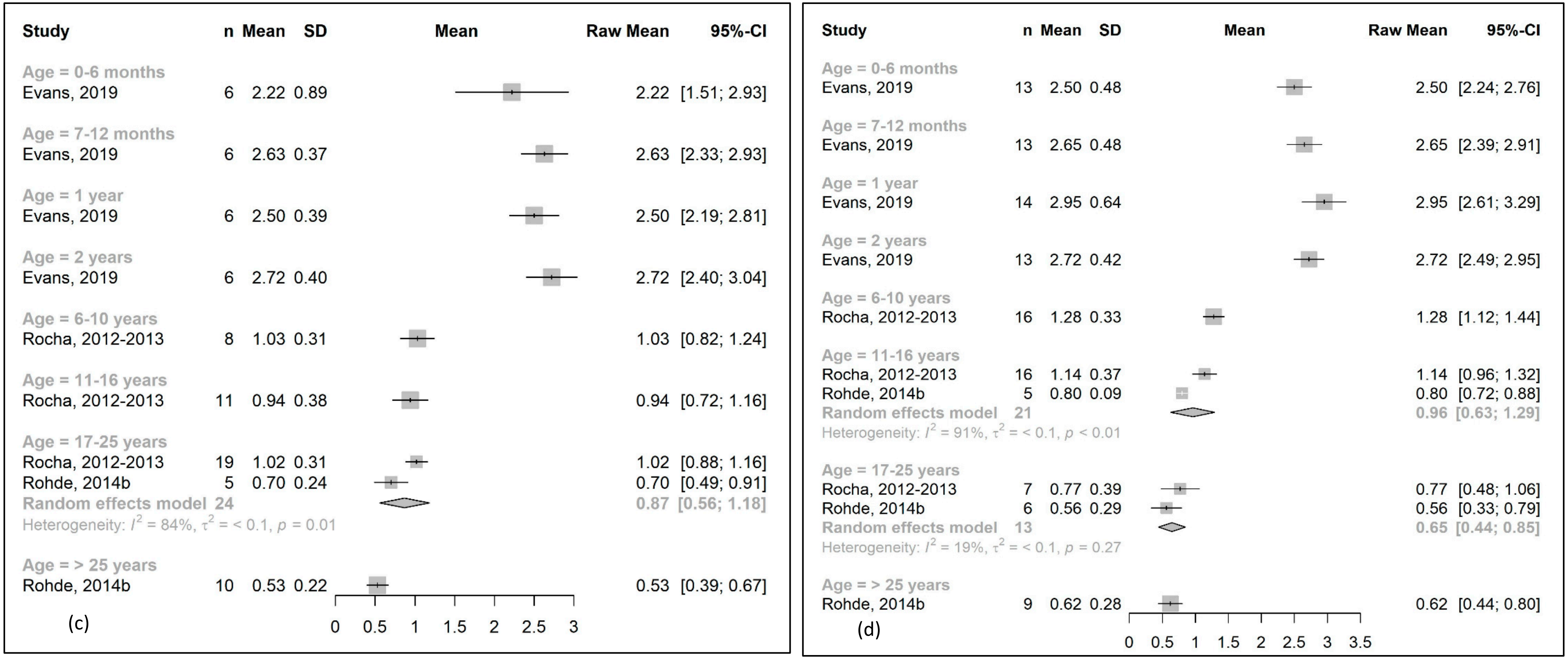


**Supplementary Figures 7c-7d.** Protein equivalent from protein substitute intakes per day (g/kg/day) of female (**c**) and male (**d**) participants in the included studies.
