# Supplementary material for: Phenylalanine Tolerance over Time in Phenylketonuria: A Systematic Review and Meta-Analysis
Source: Nutrients. 2023 Aug 8;15(16):3506. doi: 10.3390/nu15163506 (PMC10458574; doi:10.3390/nu15163506)
Supplement: Supplementary file 1 [file nutrients-15-03506-s001.zip › Supplementary Table S6_Description of dietary treatment 60723.pdf]

Supplementary Table S6. Description of dietary treatment.

Reference	Brief description of dietary treatment	Type of:			Free foods allowed	Method of dietary assessment	Source of Phe
		Exchange system	*Protein intake data*	*Protein substitute*			
Acosta et al., 1998	During the first 6 months of life, natural protein/Phe was supplied from measured amounts of infant formula (*n*=26) or human milk (*n*=9);<br><br>Phenex-1 (synthetic L-amino acid mixture) was used as the protein substitute.	No	Self-reported	Phe-free AAM	n/r	3-day food diaries	Animal protein (either breastfeeding or infant formula);<br>Beikost was introduced slowly to displace infant formula or human milk.
Aldámiz-Echevarría et al., 2013;<br><br>Aldámiz-Echevarría et al., 2014 ;<br><br>Aldámiz-Echevarría et al., 2015	*Diet only treated group:* All patients followed a Phe-restricted diet, supplemented with Phe-free AAM and low-protein foods;<br><br>The recommended daily intake of protein substitutes was determined by age and, in accordance with the Spanish protocol;<br><br>Patients were advised to divide this into at least four daily doses.	n/r	Self-reported	Phe-free AAM	After 1996:<br>Fruits and vegetables with low Phe/protein content	3-day food diaries	Intake of protein-rich foods (meat, fish, eggs, milk and dairy products, and legumes) and foods with a moderate protein content (such as cereals) was restricted.

Supplementary Table 6. Description of dietary treatment.

Reference	Brief description of dietary treatment	Type of:			Free foods allowed	Method of dietary assessment	Source of Phe
		Exchange system	*Protein intake data*	*Protein substitute*			
Alm et al., 1986	If blood Phe was >0.72 mmol/L on a normal diet (usually breast milk), Phe intake was reduced to <40 mg/kg/day;<br><br>When blood Phe<0.72 mmol/L, Phe intake was adjusted to about 60 mg/kg/day;<br><br>During the first year of life, diet was based on a combination of protein substitute and cow's milk formula/human breast milk;<br><br>Phe intake was increased by 10-20% for shorter periods of time if all blood Phe values were within therapeutic level;<br><br>Protein substitutes were given to all children with a Phe tolerance of <70 mg/kg/day;<br><br>Of 23 children, 14 continued their strict Phe-restricted diet during the study period (at 8-18y); 9/23 of children diet was normalized between 0.5-10 years of age, while maintaining the blood Phe levels between 0.25-0.72 mmoI/L.	No	Self-reported	<u>0-1 y:</u><br>Protein hydrolysate<br><br><u>After 1y:</u><br>*1965-1971:*<br>Low Phe protein source<br><br>*<u>After 1972</u>:*<br>Phe-free AAM	n/r	3-day food diaries	n/r
Daly et al., 2017	All patients followed a Phe-restricted diet, supplemented with Phe-free AAM.	Yes<br><br>(50 mg Phe)	Self-reported	<u>Study group:</u><br>GMP<br><u>Control group:</u><br>Phe-free AAM	Fruits and vegetables with a Phe content of <75 mg/100 g (except potatoes)	3-day food diaries	All foods with a Phe content above 75 mg/100 g used exchange system

Supplementary Table 6. Description of dietary treatment.

Reference	Brief description of dietary treatment	Type of: *Exchange system*	*Protein intake data*	*Protein substitute*	Free foods allowed	Method of dietary assessment	Source of Phe
Daly et al., 2019	**Three different regimens for 14 days:**<br>**R1 -** CGMP-AA + usual Phe intake;<br>**R2 -** CGM-AA + adjusted Phe intake to account for Phe from CGMP-AA (calories replaced with SLPFs or glucose polymer);<br>**R3 -** Usual AAM and usual Phe intake.	Yes<br><br>(50 mg Phe)	Self-reported	Study period:<br>GMP (20 g PE)<br><br>At baseline:<br>Phe-free AAM (20 g PE)	Fruits and vegetables with a Phe content of <75 mg/100 g (except potatoes)	3-day food diaries	All foods with a Phe content above 75 mg/100 g used exchange system
Dobbelaere et al., 2003	The dietary regimen was based on synthetic or semisynthetic alimentary products with no or low Phe content to cover protein requirements, and on certain natural foods with a lower Phe content (especially fruit and vegetables) to take care of the remaining needs.	No	Self-reported	Phe-free AAM	n/r	4-day food diaries	n/r
Evans et al., 2017	All patients followed a Phe-restricted diet, supplemented with Phe-free AAM;<br><br>Some fruits and vegetables that contain small amount of Phe were allowed freely;<br><br>Patients were advised to consume 3–4 AAMs/day.	Yes<br><br>(50 mg Phe)	Self-reported	Phe-free AAM	Some fruits and vegetables with low protein content ('uncounted foods')	4-day food diaries	All Phe containing foods and 'uncounted' foods with small amounts of Phe were included
Evans et al., 2018	Phe-free infant AAM in combination with breast (n=6) or infant formula milk (n=25) to provide a source of Phe;<br><br>When weaning, a Phe-free AAM weaning protein substitute was commenced, breast feeds or standard infant formula were gradually replaced by weaning foods containing an equivalent Phe content.	Yes<br><br>(50 mg)	Prescribed	Phe-free AAM	Fruits and vegetables with a Phe content of <75 mg/100 g and low protein special foods	24h recall	Primary Phe source:<br>Breast milk, infant formula;<br><br>Other:<br>Low protein fruits and vegetables

Supplementary Table 6. Description of dietary treatment.

Reference	Brief description of dietary treatment	Type of: *Exchange system*	*Protein intake data*	*Protein substitute*	Free foods allowed	Method of dietary assessment	Source of Phe
Evans et al., 2019	The weaning protein substitute was gradually introduced at 4-6 months of life, after low protein solids were established.<br><br>Subjects continued to drink Phe-free infant formula as their main source of Phe-free L-amino acids during the early stages of weaning;<br><br>The clinical dietitian determined the dose and increments of the weaning protein substitute.	Yes<br><br>(50 mg Phe)	Self-reported & Prescribed	Phe-free AAM	n/r	24h dietary record	n/r
Evers et al., 2018	Dietary assessments and recommendations for protein and AAM intake were done by different dieticians using the same national dietary guidelines.<br><br>Natural protein recommendation according to national guidelines:<br>1-14 y: ≥ 0.90 g/kg/day;<br>14y and older: ≥0.80 g/kg/day<br><br>Mineral and vitamin supplements were prescribed as required.	n/r	Prescribed	Phe-free AAM	n/r	n/r	n/r

Supplementary Table 6. Description of dietary treatment.

Reference	Brief description of dietary treatment	Type of:			Free foods allowed	Method of dietary assessment	Source of Phe
		Exchange system	*Protein intake data*	*Protein substitute*			
Ferguson et al., 1996	**Three types of study regimen for 2 days:**<br>**Group A -** Nationally recommended Phe-restricted diet, distributing their protein substitute and Phe exchanges evenly over the day - 4 x 25% of daily Phe exchanges and PS;<br><br>**Group B -** Daily Phe exchange distribution = 12.5% at breakfast and lunch + 50% at dinner + 25% at supper snack; daily PS distribution = 50% at breakfast and dinner + No PS at lunch and supper;<br><br>**Group C -** Daily Phe exchange distribution = 37.5% at breakfast + 12.5% at lunch + 50% at dinner + no exchange at supper; daily PS distribution = 33.3% at breakfast, lunch and dinner + No PS at supper.	Yes<br><br>(50 mg Phe)	Self-reported & Prescribed	Phe-free AAM	'Free foods' that are allowed without restriction in the NSPKU dietary information pack (1994–95)	24h dietary record	n/r
Giovaninni et al., 2014	All patients followed a Phe-restricted diet, supplemented with Phe-free AAM started as soon after birth as possible and continued throughout adulthood to maintain target plasma Phe levels.	n/r	Self-reported	<u>Study group:</u> Prolonged-release Phe-free AAM<br><br><u>Control group:</u> Conventional protein substitute. Phe-free AAM	n/r	3-day food diaries	n/r

Supplementary Table 6. Description of dietary treatment.

Reference	Brief description of dietary treatment	Type of: *Exchange system*	*Protein intake data*	*Protein substitute*	Free foods allowed	Method of dietary assessment	Source of Phe
Gökmen-Özel et al., 2011	All patients followed a Phe-restricted diet, supplemented with Phe-free AAM (control vs. trial protein substitute for 14 days).	Yes<br><br>(50 mg Phe)	Self-reported	Control PS:<br>Phe-free AAM: Median CHO/prot. equivalent ratio of 1:1 [0.7:1-1.6:1]<br><br>Trial PS:<br>Phe-free AAM: Lower CHO liquid PS (CHO/prot. equivalent ratio of 0.5:1	Fruits and vegetables with a Phe content of <75 mg/100 g and low protein special foods	3-day food diaries	n/r
Green et al; 2019	*Diet adherent group*: All patients were following a Phe restricted diet and were reported to be generally very adherent in taking their prescribed amount of low-phenylalanine protein substitute.	Yes<br><br>(50 mg Phe)	Self-reported	Phe-free AAM	Fruits and vegetables with a Phe content of <75 mg/100 g	3-day food diaries	n/r
Hoeksma et al., 2005	n/r	n/r	Self-reported[a]	Phe-free AAM	n/r	n/r	n/r
Huemer et al., 2007	Phe-restricted diet supplemented with a Phe-free AAM;<br><br>Natural protein and Phe-free amino acids were used to achieve a total protein intake exceeding 20-40% of the RDA for healthy Dutch children and adolescents:<br><br>AAM was prescribed to be ingested in three portions combined with meals.	n/r	Self-reported	Phe-free AAM	n/r	3-day food diaries	n/r

Supplementary Table 6. Description of dietary treatment.

Reference	Brief description of dietary treatment	Type of:			Free foods allowed	Method of dietary assessment	Source of Phe
		Exchange system	*Protein intake data*	*Protein substitute*			
Kindt et al., 1983	Half of the patients were given a daily protein intake according to the RDA (received about 60% more Albumaid XP than FAO), and half of the patients followed a dietary protein intake based on the recommendations of FAO.	n/r	n/r	Phe-free protein hydrolysate	Foods with negligible amounts of protein content (e.g. protein-free bread, spaghetti, biscuits, margarine, jam, and honey or small amounts of ketchup and mustard)	n/r	Plant foods + milk;<br><br>The protein content of vegetables and fruits were included.
MacDonald et al., 2006	All children followed a strict low Phe diet supplemented with Phe-free protein substitute and low Phe foods;<br>**Protocol A -** Children were given 2 g/kg/day protein equivalent from protein substitute for 14 days;<br><br>**Protocol B -** Children were given 1.2 g/kg/day protein equivalent from protein substitute for 14 days after wash-out (according to UK dietary reference values);<br><br>**Wash-out -** Usual protein substitute intake.	Yes<br><br>(50 mg Phe)	Self-reported	Phe-free AAM	Low Phe foods, e.g. most fruits, many vegetables, and special low protein products (in usual quantities)	7-day food diaries	n/r
MacDonald et al; 2003	All children followed a strict low Phe diet supplemented with a Phe-free protein substitute; and low Phe foods.	Yes<br><br>(50 mg Phe)	Prescribed	Phe-free AAM	Low Phe foods, e.g. most fruits, many vegetables, and special low protein products (in usual quantities)	2-day food diaries	n/r

Supplementary Table 6. Description of dietary treatment.

Reference	Brief description of dietary treatment	Type of: *Exchange system*	*Protein intake data*	*Protein substitute*	Free foods allowed	Method of dietary assessment	Source of Phe
MacDonald et al., 1996	All children followed a strict low Phe diet supplemented with a Phe-free protein substitute; and low Phe foods.	Yes<br><br>(50 mg Phe)	Prescribed & Self-reported (only for Phe)	Phe-free AAM	Free foods that contain 0-3 g of protein/100 g; e.g. most fruits, many vegetables, and special low protein products	2-day food diaries	n/r
Pinto et al., 2019	All patients followed a low-Phe diet supplemented with a low-Phe/Phe-free protein substitute;<br><br>After additional natural protein challenge:<br>77% of patients increased protein intake from animal sources, and 23% from plant sources.	Yes<br><br>(20 mg Phe)	Self-reported	Phe-free AAM in n=36<br><br>GMP + Phe-free AAM in n=4	Special low protein products	24h recall	Animal and plant sources
Ponzone et al; 2008	14 patients were breastfeeding; 2 patients were formula-feeding; 5 patients were on mixed feeding;<br><br>At 10 to 25 days after initial screening, all patients were put on a Phe free diet regardless of their plasma level;<br><br>Dietary Phe tolerance was evaluated at 2 months of age.	n/r	Self-reported	n/r	n/r	n/r	n/r
Rocha et al., 2012 & 2013	All patient followed a traditional treatment based on a low Phe diet, supplemented with a Phe-free protein substitute	Yes<br><br>(20 mg Phe)	Self-reported	Phe-free AAM	Special low protein products	24h recall	n/r

Supplementary Table 6. Description of dietary treatment.

Reference	Brief description of dietary treatment	Type of:			Free foods allowed	Method of dietary assessment	Source of Phe
		Exchange system	*Protein intake data*	*Protein substitute*			
Rohde et al., 2012 & 2014a	**1st phase (2-weeks) –** Restricted fruit-vegetable intake vs. Free consumption of fruits and vegetables with <75mg Phe/100 g;<br><br>**2nd phase (2-weeks) -** Crossover;<br><br>**3rd & 4th phase (total of 12 mo):** Free consumption of fruits and vegetables with <75mg Phe/100 g.	No	Self-reported	n/r	Fruits and vegetables with a Phe content of <75 mg/100 g	5-day food diaries at each phase	n/r
Rohde et al., 2015	**Regime 1 –** All ingested foods and drinks were included in the Phe calculation;<br>**Regime 2 -** Foods with a very low Phe content, e.g. <10 mg/100 g food; were excluded from Phe calculation;<br>**Regime 3 –** A combination of free consumption of low protein foods (<10 mg Phe/100 g), fruit and vegetables (<75 mg Phe/100 g) with calculating the exact Phe content of all other foods;<br>**Regime 4 -** A "simplified diet", consists of estimating the Phe content according to certain principles;<br>**Regime 5 -** Despite other instructions some patients only estimated their protein consumption and even consumed varying amounts of protein rich foods e.g. meat, milk or eggs, this regime was self-selected by the families and was never recommended;<br><br>Dietary regimes 1–4 were chosen by the different metabolic centres.	n/a	Self-reported	n/r	Depending on the regime	3-day food diaries	Both animal and plant sources depending on the regime

Supplementary Table 6. Description of dietary treatment.

Reference	Brief description of dietary treatment	Type of:			Free foods allowed	Method of dietary assessment	Source of Phe
		Exchange system	*Protein intake data*	*Protein substitute*			
Rohde et al., 2014b	Patients followed a lifelong Phe-restricted diet, supplemented with a Phe-free AAM;<br><br>**Group 1 -** patients who did not use an AAM (8 on BH4, 5 with high residual enzyme);<br><br>**Group 2:** patients receiving any AAM (13 on BH4, 41 on diet therapy):<br>*Group 2a* - Total protein intake more than 120% of recommendations;<br>*Group 2b* - Total protein intake less than 120% of recommendations.	No	Self-reported	Phe-free AAM	n/r	3-day food diaries	n/r
Schulpis et al., 2013	All patients followed a Phe-restricted diet supplemented with a Phe-free AAM;<br><br>The quantity of Phe free formula replacement depended on their age, body weight and residual activity of Phe hydroxylase;<br><br>PKU patients were requested to discontinue their vitamin supplements only for the last 60 days.	n/r	Self-reported	Phe-free AAM	n/r	30-day food diaries	n/r
Stockler-Ipsiroglu et al., 2015	Patients were treated according to a standard medical nutrition therapy protocol including a Phe restricted diet, medical Phe-free AAM, and low protein foods.	Yes<br><br>(47 mg)	Prescribed	Phe-free AAM	n/r	n/a	n/r<br><br>(one patient with mHPA was on normal diet until 1y of age)

Supplementary Table 6. Description of dietary treatment.

Reference	Brief description of dietary treatment	Type of:			Free foods allowed	Method of dietary assessment	Source of Phe
		Exchange system	*Protein intake data*	*Protein substitute*			
Sweeney et al., 2012	Traditional Australian dietary management of PKU was followed by the patients, consisting of a medical formula providing Phe-free amino acids, vitamins, minerals, and trace elements.<br><br>Special low protein products and foods that contain minimal amounts of Phe such as fats and sugars were considered as free.	Yes<br><br>(15 mg vs. 50 mg)	Prescribed	Phe-free AAM	**Phase I -** Foods containing <20 mg Phe/serving were considered “free" (e.g. some fruits and vegetables)<br><br>**Phase II -** Foods containing <50 mg Phe/serving were considered “free", but were given a serve size limit (e.g. fruits and vegetables), commercial snack foods with <0.3 g of protein/serve.	3-day food diaries (but not analysed)	Phe is provided in carefully measured quantities of low Phe foods, (some cereals and most fruits and vegetables), using a 15 mg Phe unit exchange system.
Thiele et al., 2017	Patients followed a protein- and/or Phe-restricted diet, supplemented with a Phe-free protein substitute starting from neonatal period;<br><br>Patients with mHPA (dried blood Phe concentrations <600 µmol/L) were not treated by any dietary therapy;<br><br>Note: Diet was interrupted during childhood in 28 patients born before 1980, according to the former guidelines, and was reintroduced in 19 before adulthood upon recommendation of their paediatrician.	n/r	Self-reported	Infants born before 1989: Protein hydrolysate<br><br>Infants born after 1989: Phe-free AAM	n/r	n/r	n/r

Supplementary Table 6. Description of dietary treatment.

Reference	Brief description of dietary treatment	Type of:			Free foods allowed	Method of dietary assessment	Source of Phe
		Exchange system	*Protein intake data*	*Protein substitute*			
Trefz et al., 2009	Subjects were instructed to maintain a stable, Phe restricted diet throughout the study, monitored by diaries kept for 3 days of each week containing a record of weighed or measured amounts of all food and drinks ingested, including medical foods.	n/r	Self-reported	n/r	n/r	3-day food diaries	n/r
van Spronsen et al., 2009	n/r (early and continuously treated Dutch PKU patients up to 10 years of age)	n/r	Prescribed	n/r	None	n/a	Calculations on dietary Phe intake also included intake of natural protein containing very small amounts of Phe.
Wendel et al., 1990	After plasma Phe had decreased to a near normal level and had stabilised for at least 48 h after birth, dietary management was accomplished by prescribing Phe and other foods according to nutritional guidelines;<br><br>The diet was supplemented with calculated amounts of whole milk, various low protein foods such as cereals, fruits and vegetables, low protein bread and pasta to meet individual energy and nutrient needs;<br><br>Nutrient intakes were calculated based on the recommendations of the German Nutrition Society which refer to a safe level of intake according to age.	n/r	Prescribed	Protein hydrolysate and Phe-free AAM	n/r	n/r[b]	Milk, plant based protein and low protein foods.

Abbreviations: PKU, phenylketonuria; Phe, phenylalanine; AAM, amino acid mixture; GMP: Glycomacropeptide; PE, protein equivalent; PS, protein substitute; CHO, carbohydrate; prot., protein; RDA, recommended dietary allowances; FAO, Food and Agriculture Organisation; mHPA, mild hyperphenylalaninemia; BH4, tetrahydrobiopterin; n/r, not reported; n/a,

not applicable; [a] Prescribed intake was reported only for energy intake during the first year of life. [b] The diet plans regarding the intakes of Phe as well as of protein substitutes were recorded at the end of each 6-month period from 6 months to 6 years of age.
