# Supplementary material for: Phenylalanine Tolerance over Time in Phenylketonuria: A Systematic Review and Meta-Analysis
Source: Nutrients. 2023 Aug 8;15(16):3506. doi: 10.3390/nu15163506 (PMC10458574; doi:10.3390/nu15163506)
Supplement: Supplementary file 1 [file nutrients-15-03506-s001.zip › Supplementary Table S7_Metabolic control_06072023.pdf]

Supplementary Table S7. Data on metabolic control of patients with PKU in the included studies.

Reference	Centres' practice/protocol for monitoring and data reported	Target blood Phe ranges for age	Blood Phe levels for age<br>Mean ± SD [Median, range]
Acosta et al., 1998	***Protocol:*** Blood was drawn monthly by venipuncture for analysis of plasma amino acids.<br><br>***Data:*** Blood Phe levels taken after 2 to 4 h of eating.	120 to 360 μmol/L	**1 mo:** 147±20 μmol/L (N=20)<br>**2 mo:** 267±46 μmol/L (N=33)<br>**3 mo:** 349±46 μmol/L (N=31)<br>**4 mo:** 356±44 μmol/L (N=34)<br>**5 mo:** 297±35 μmol/L (N=33)<br>**6 mo:** 308±38 μmol/L (N=32)<br>**During the study:** 297±41 μmol/L (N=35)
Aldámiz-Echevarría et al., 2014	***Protocol:*** Blood Phe concentrations were regularly measured in dried blood spots;<br>**0 to 6 mo:** Weekly, **6 mo – 2 y:** Every other week, **2 y:** ≤ Monthly; More frequent check-ups during intercurrent illnesses, patients failing to adhere to the diet, etc.	**<6 y:** < 360 μmol/L,<br>**6-10 y:** < 480 μmol/L,<br>**>10y:** < 600 μmol/L	Data not shown
Aldámiz-Echevarría et al., 2013	***Protocol:*** Blood Phe concentrations were regularly measured in dried blood spots;<br>**0 to 6 mo:** Weekly, **6 mo – 2 y:** Every other week, **2 y:** ≤ Monthly; More frequent check-ups during intercurrent illnesses, patients failing to adhere to the diet, etc.<br><br>***Data:*** Mean values reported at baseline, and every 6 mo until the end of 2y or 5y follow-up.	**<6 y:** < 360 μmol/L,<br>**6-10 y:** < 480 μmol/L,<br>**>10y:** < 600 μmol/L	**BH4 group (baseline):**<br>**2y FU:** 255.2 ± 146.8 μmol/L (N=36)<br>**5y FU:** 204.0 ± 143.9 μmol/L (N=10)<br>**Diet-only treated group (with 2y FU; N=72):**<br>**Baseline:** 418.4 ± 339.6 μmol/L<br>**6 mo:** 396.9 ± 285.0 μmol/L<br>**1y:** 384.9 ± 258.6 μmol/L<br>**1.5y FU:** 430.5 ± 304.0 μmol/L<br>**2y FU:** 468.6 ± 334.4 μmol/L<br>**Diet-only treated group (with 5y FU; N=20)**<br>**Baseline:** 391.8 ± 233.5 μmol/L<br>**6 mo:** 466.7 ± 295.5 μmol/L<br>**1y:** 352.8 ± 210.6 μmol/L<br>**1.5y:** 533.5 ± 282.2 μmol/L<br>**2y:** 345.2 ± 163.4 μmol/L<br>**2.5y:** 395.3 ± 216.2 μmol/L<br>**3y:** 439.0 ± 313.4 μmol/L<br>**3.5y:** 484.9 ± 272.5 μmol/L<br>**4y:** 287.1 ± 165.0 μmol/L<br>**4.5y:** 401.1 ± 235.2 μmol/L<br>**5y:** 440.6 ± 298.4 μmol/L

Supplementary Table 7. *Cont.*

Reference	Centres' practice/protocol for monitoring and data reported	Target blood Phe ranges for age	Blood Phe levels for age<br>Mean ± SD [Median, range]
Aldámiz-Echevarría et al., 2015	***Protocol:*** Blood Phe concentrations were regularly measured in dried blood spots;<br>**0 to 6 mo:** Weekly, **6 mo – 2 y:** Every other week, **2 y: ≤** Monthly; More frequent check-ups during intercurrent illnesses, patients failing to adhere to the diet, etc.<br><br>***Data:*** Mean values reported at study baseline, at 6 mo, and 1 y follow-up	**<6 y:** < 360 µmol/L,<br>**6-10 y:** < 480 µmol/L,<br>**>10y:** < 600 µmol/L	<u>**BH4 group (baseline)**</u><br>286.9 ± 153.7 µmol/L (N=22)<br><br><u>**Diet-only treated group:**</u><br>**Baseline:** 306.8 ± 167.8 µmol/L (N=44)<br>**6 mo:** 358.2 ± 232.6 µmol/L (N=44)<br>**1y:** 353.7 ± 236.4 µmol/L (N=44)
Alm et al., 1986	***Protocol:*** Blood Phe samples were collected at home by the parents or the patients.<br>**0-3 y:** 2-4 times per month<br>**>3y:** 1-2 times per month<br><br>***Data:*** % Phe levels within target range or a period of 8 to 18y	**0-8 y:** 0.24-0.72 µmol/L<br>**>8y:** 0.24-1.0 µmol/L	**Good compliance in N=11;**<br>>75% of Phe levels were within target range<br>**Fair compliance in N=5;**<br>50-75% of Phe levels were within target<br>**Poor compliance in N=5;**<br><50% of Phe levels were within target<br>**Unknown in N=2 (Normal IQ)**
Daly et al., 2017	***Protocol:*** Weekly; overnight fasting morning blood spots were collected on filter cards by trained caregivers at home.<br><br>***Data:*** Median of blood Phe collected during 6 mo compared with median from previous 12 mo entering the study.	**5-10y:** 120–360 µmol/L<br>**11-16y:** 120–600 µmol/L	**GMP group - 12 mo pre-GMP:**<br>[275 µmol/L; N=12]<br>**GMP group - 6 mo during GMP:**<br>[317 µmol/L; N=12]<br>**AAM group - 12 mo AAM (pre-study):**<br>[325 µmol/L; N=9]<br>**AAM group - 6 mo during study:**<br>[280 µmol/L; N=9]
Daly et al., 2019	***Protocol:*** Weekly overnight fasting morning blood spots were collected by trained caregivers at home. During the last 48h of each study period (days 13 and 14), trained caregivers collected four hourly blood spots on filter cards at home.<br><br>***Data:*** 70% of blood Phe concentrations within the Phe target range for age, for 6 mo before starting the study, and median Phe over the 24h in each study period (on days 13-14) at: 08.00, 12:00, 16:00, 20:00 and 24:00.	**5-10y:** 120–360 µmol/L<br>**11-16y:** 120–600 µmol/L	**R1:** [290 µmol/L; 30–580 µmol/L; N=19]<br>**R2:** [220 µmol/L; 10–670 µmol/L; N=19]<br>**R3:** [165 µmol/L; 10–640 µmol/L; N=19]

Supplementary Table 7. *Cont.*

Reference	Centres' practice/protocol for monitoring and data reported	Target blood Phe ranges for age	Blood Phe levels for age<br>Mean ± SD [Median, range]
Dobbelaere et al., 2003	***Protocol:*** Fasting blood Phe levels by measurement on Guthrie card (dried blood spot) on a regular basis (at least monthly)<br><br>***Data:*** Mean, median and range of serum Phe levels.	<480 µmol/L (8 mg/dl)	457.3±313.8 µmol/L (N=19)<br>[347 µmol/L; 25-1172 µmol/L]
Evans et al., 2017	Not reported	Not reported	Not reported
Evans et al., 2018	***Protocol:*** Weekly fasting levels<br><br>***Data:*** Median and range of serum Phe levels during the first 2 years of life.	120–360 µmol/L	**From weaning to 12 mo of age:**<br>[175 µmol/L; 90–310 µmol/L; N=31];<br>**12-24 mo of age:**<br>[180 µmol/L; 100–400 µmol/L; N=31]
Evans et al., 2019	Not reported	Not reported	Not reported
Evers et al., 2018	***Protocol:*** Blood Phe was measured in plasma or dried bloodspot; at least 5 samples per period<br><br>***Data:*** In BH4 group, median Phe concentration at baseline was calculated from data on a period of 12 mo before the start of BH4 testing. Median, IQR was reported at baseline and 5y follow-up.	≤12 years: 120–360µmol/L<br>>12 years: 120–600µmol/L	**BH4 group (baseline):**<br>[322 µmol/L; IQR: 239-470 µmol/L; range: 178-605 µmol/L; N=18]<br><br>**Diet-only group (baseline):**<br>[395 µmol/L; IQR: 275-652 µmol/L; range: 201-800 µmol/L; N=21]<br>**Diet-only group (5y follow-up):**<br>[354 µmol/L ; IQR: 222-690 µmol/L; range: 137-1084 µmol/L; N=21]
Ferguson et al., 1996	***Protocol:*** During study weekend;<br>**Friday:** 1h after lunch (baseline),<br>**Saturday:** 1 h after each meal (breakfast, lunch, dinner, supper) - representing maximum post-meal blood Phe,<br>**Sunday:** In the morning (after fasting).<br><br>***Data:*** Mean, median, range of serum Phe levels.	120-700 µmol/l	395±71.8 µmol/L (N=12)<br>[384.1 µmol/L; 330-472 µmol/L]<br><br>**Serum Phe over 24h:**<br>**Group A:** [329.6 µmol/L; 187-574; N=4]<br>**Group B:** [384.1 µmol/L; 267-534; N=3]<br>**Group C:** [472 µmol/L; 240-656; n=5]

Supplementary Table 7. *Cont.*

Reference	Centres' practice/protocol for monitoring and data reported	Target blood Phe ranges for age	Blood Phe levels for age<br>Mean ± SD [Median, range]
Giovaninni et al., 2014	***Protocol:*** Fasting plasma Phe levels taken at 9 a.m. ± 1h on the day of recruitment and at the end of trial.<br><br>***Data:*** Mean and median plasma Phe levels.	2–6 mg/dL	**Test PS group: N=27**<br>**Baseline:** 6.22±5.4 mg/dl [5.43 mg/dl]<br>**At the end of trial:** 4.47±4.08 mg/dl [3.7 mg/dl]<br><br>**PKU conventional PS group: N=28**<br>**Baseline:** 5.96±5.02 mg/dl [5.52 mg/dl]<br>**At the end of trial:** 5.56±4.92 mg/dl [4.93 mg/dl]
Gökmen-Özel et al., 2011	***Protocol:*** Once daily finger prick blood samples (fasting pre-breakfast at standardised times) were taken on day 5–7 of the pre-study period and on days 12–14 in part A (subjects took control PS) and B (subjects took trial PS). These daily bloods were collected over 3 days for each trial period to allow for any variability in daily plasma Phe concentrations.<br><br>***Data:*** Median plasma Phe levels;<br>**Pre-study:** On days 5-7 (1st wk)<br>**Part A:** On days 12-14 (2-3rd wk)<br>**Part B:** On days 12-14 (4-5th wk)	120-360 μmol/L	**Pre-study (0-7 d):**<br>[240 μmol/L; 150-420 μmol/L; N=14]<br><br>**Part A (patients took Control PS for 14d):**<br>[275 μmol/L; 150-590 μmol/L; N=14]<br><br>**Part B (patients took Trial PS for 14d):**<br>[230 μmol/L; 41-710 μmol/L; N=14]
Green et al; 2019	***Protocol:*** Dried blood spots following an overnight fast by fingertip puncture was collected.<br><br>***Data:*** Mean blood Phe from past 3 historical blood Phe tests prior to recruitment.	120-700 μmol/L at time of recruitment	**Historical blood Phe (from records):**<br>618±292 μmol/L (N=16)<br><br>**During study:** 464 ± 196 μmol/L (N=16)
Hoeksma et al., 2005	Not reported	Not reported	Not reported
Huemer et al., 2007	***Protocol:*** Blood Phe was measured at baseline and after 6 and 12 mo from dried blood spots using ion-exchange chromatography (for clinical follow-up, not for study purposes).<br><br>***Data:*** Mean, median and range of blood Phe was measured at baseline and after 6 and 12 mo.	**<10y:** 40-240 μmol/L (0.7–4 mg/dl)<br><br>**10-15y:** 40-900 μmol/L (0.7–15 mg/dl)	**Mean Phe level was elevated, median was normal;**<br>456±432 μmol/L (N=34) [204 μmol/L; 24-1386 μmol/L]<br>**<10 years:** 8/17 (47%) had Phe concentrations exceeding therapeutic levels;<br>**10-15y of age:** Within therapeutic levels, 534±324 μmol/L [408 μmol/L; 174-1188 μmol/L]<br>**>15y of age:** Within therapeutic levels, 444±228 μmol/L [483 μmol/L; 150-714 μmol/L]

Supplementary Table 7. *Cont.*

Reference	Centres' practice/protocol for monitoring and data reported	Target blood Phe ranges for age	Blood Phe levels for age<br>Mean ± SD [Median, range]
Kindt et al., 1983	***Protocol:*** Plasma Phe was determined by the fluorimetric method; samples were usually drawn after an overnight fast, in some cases 4 h after the last meal, and sent to the hospital every week for the 1st year of life, every 2 week for the 2nd year and once every 1 or 2 month thereafter. Minor colds and any diseases were noted.<br><br>***Data:*** Range of means.	182-667 µmol/L<br><br>*Phe deficit: <121 µmol/L; Catabolic state/Too high Phe intake: Phe>970 µmol/L*	Metabolic control was equally well in both groups:<br>RDA group: N=8<br>**0-12 mo:** 303-752 µmol/L<br>**12-24 mo:** 273-642 µmol/L<br>**FAO group: N=8**<br>**0-12 mo**: 376-673 µmol/L<br>**12-24 mo:** 436-545 µmol/L
MacDonald et al., 2006	**Protocol:** Twice daily finger prick blood samples (fasting pre-breakfast and pre-evening meal at standard times) were taken on day 8–14 of each trial period by parents/carers. Twice daily blood samples were collected for 3 days during a baseline and washout period. Frequency of monitoring:<br>**0-5y:** Weekly; **>5y:** Fortnightly<br><br>***Data:*** Mean, median, range of Phe concentrations were estimated from morning and evening blood samples for each subject.	**0-5y:** 120- 360 µmol/L;<br><br>**School age:** 120-480 µmol/L	**Low dose PS period:**<br>505±206.8 µmol/L (N=25)<br>[530 mmol/L; 140-910 µmol/L];<br><br>**High dose PS period:**<br>214.8±74.9 µmol/L (N=25)<br>[200 µmol/L; 100-390 µmol/L]
MacDonald et al; 2003	***Protocol:*** Four-hourly skin puncture blood specimens were collected for 48 h in each study protocol. Frequency of monitoring:<br>**0-5y:** Weekly; **>5y:** Fortnightly	**0-5y:** 120- 360 µmol/L;<br><br>**School age:** 120-480 µmol/L	None of the blood Phe concentrations exceeded the MRC (1993) upper recommended limits in any protocol (n=16).
MacDonald et al., 1996	***Protocol:*** Twice daily, 4 days a week (same days as diet diaries) during the 21 days study period.<br><br>***Data:*** Mean, median, range of Phe concentrations taken twice daily.	**0-4y:** 120-360 µmol/L<br>**5-10 y:** 120-480 µmol/L<br>**11y<:** 120-700 µmol/L	**Morning:**<br>282±162 µmol/L (N=19)<br>[230 µmol/L; 127-660 µmol/L]<br><br>**Evening:**<br>243±184 µmol/L (N=19)<br>[176 µmol/L; range= 68-659 µmol/L]<br><br>There was a significant difference between mean early morning and late afternoon plasma Phe and a widespread variation in the change between early morning and late afternoon concentrations: mean plasma Phe change -40±62 umol/l.

Supplementary Table 7. *Cont.*

Reference	Centres' practice/protocol for monitoring and data reported	Target blood Phe ranges for age	Blood Phe levels for age Mean ± SD [Median, range]
Pinto et al., 2019	***Protocol:*** Median blood Phe levels were measured by fasting blood spots performed at home. Phe levels were collected for a median of 6 mo before the first and second dietary assessment, with a median of 6 (range 2–20) (assessment period 1) vs.11 (range 2–23) blood spots (assessment period 2).<br>Frequency of recommended monitoring:<br>**<1y:** Once weekly; **1-12y:** Once every 2 weeks; **≥12 y:** Once monthly<br><br>***Data:*** Median and IQR of Phe levels	**0-12 y:** 120- 360 µmol/L;<br>**School age (12y<):** 120-480 µmol/L<br><br>**Tyr levels:** <480 µmol/L	[279 µmol/L; IQR: 203-360 µmol/L] n=40
Ponzone et al; 2008	***Protocol:*** Monitoring of plasma Phe concentration was performed by amino acid chromatography:<br>**0-1y:** Weekly; >**1y:** Every 2 months.	<600 µmol/L (10 mg/dl)	Not reported
Rocha et al., 2012 & 2013	***Protocol:*** Frequency of recommended monitoring:<br>**<1y:** Once weekly; **1-12y:** Once every 2 weeks; >**12 y:** Once monthly<br><br>***Data:*** Median of Phe concentrations during the year before the evaluation was used to assess the metabolic control.	**Good control:**<br>**<12y:** Median Phe <6 mg/dL (360 µmol/L)<br><br>**>12y:** Median Phe<8 mg/dL (480 µmol/L)	Globally, 68.5% of patients had a good metabolic control n=89;<br><br>**Good metabolic control range:**<br>**<12y:** 93.2% were within this range;<br>**>12y**: 50% were within this range.
Rohde et al., 2012 & 2014a	***Protocol (2014a):*** Phe concentration was determined bi- to four-weekly. Dried blood Phe was recorded over a 3-day period after 6 (study phase 3) and 12 mo (study phase 4)<br><br>***Protocol (2012):***<br>During the study samples of capillary whole blood were taken on filter paper daily between 07:00 and 09:00 hours after an overnight fast. Twice a week, they mailed the filter paper cards to the hospital.	40–240 µmol/L	***2014a:***<br>Throughout the study, mean dried blood Phe concentrations remained within the recommended range<br><br>***2012:*** Average dried-blood Phe concentrations remained stable and were within the recommended range throughout the study;<br><br>**At baseline:** 230 ± 63 µmol/L [N=14; 121-306 µmol/L]<br>**Restricted fruits-vegetables intake:** 246±140 µmol/L<br>**Unrestricted intake:** 243±137 µmol/L

Supplementary Table 7. *Cont.*

Reference	Centres' practice/protocol for monitoring and data reported	Target blood Phe ranges for age	Blood Phe levels for age<br>Mean ± SD [Median, range]
Rohde et al., 2015	**Protocol:** Last 6 Phe-concentrations in dried blood prior to study entry.<br><br>***Data:*** Median, range of last 6 Phe concentrations	Upper therapeutic limit of Phe:<br>**<10y:** 240 µmol/L<br>**>10y:** 900 µmol/L	**1-9y:** Good to acceptable metabolic control;<br>**Diet 1:** [161 µmol/L; 77-829 µmol/L; N=22]<br>**Diet 2:** [229 µmol/L; 92-554 µmol/L; N=25]<br>**Diet 3:** [236 µmol/L; 75-744 µmol/L; N=30]<br>**Diet 4:** [249 µmol/L; 78-531 µmol/L; N=25]<br>**Diet 5:** [288 µmol/L; 156-529 µmol/L; N=6]<br>**Total: [**236 µmol/L; 75-829 µmol/L; N=108]<br>**10-15y:** Good to acceptable metabolic control;<br>**Diet 1:** [361 µmol/L; 119-810 µmol/L; N=5]<br>**Diet 2:** [565 µmol/L 170-1157 µmol/L; N=10]<br>**Diet 3:** [532 µmol/L 119-844 µmol/L; N=13]<br>**Diet 4:** [627 µmol/L 184-812 µmol/L; N=10]<br>**Diet 5:** [629 µmol/L 584-802 µmol/L; N=3]<br>**Total:** [565 µmol/L; 119-1157 µmol/L; N=41]
Rohde et al., 2014b	Not reported	Not reported	Almost all included patients show Phe concentrations within their age-specific target ranges, according to the German guidelines.
Schulpis et al., 2013	***Protocol:*** Blood was taken after 9-10 h fasting for the evaluation of Phe (Phe instant Phe blood level at the moment of sampling). Phe was measured with a standardized enzymatic assay in dried blood samples on filter paper.<br><br>***Data:*** Mean, SD of blood Phe levels.	Not reported	**Blood Phe; Group B (strictly adherent to the diet]**<br>492.0±100.0 µmol/L; N=30
Stockler-Ipsiroglu et al., 2015	***Protocol:*** Blood samples were collected after a 3–6 h fasting for children <2 y and after a 6–10 h overnight fast in older children. Dried blood spots were obtained during regular home-based monitoring, or in plasma from blood samples obtained during a clinic visit.<br>Minimum frequency;<br>**<6 mo:** Twice weekly; **6-18 mo:** Once weekly;<br>**18 mo-12y:** Every 2 weeks; >**12 y:** Monthly<br><br>***Data:*** Mean, median, range of blood Phe at baseline (1 mo pretreatment)	2–6 mg/dl (120–360 µmol/l)<br><br>**In patients >12y:**<br>< 10 mg/dl (<600 µmol/l)	**Baseline**:<br>4.53±1.23 mg/dl<br>[4.61 mg/dl; 3.13-6.59 mg/dl; N=11]

Supplementary Table 7. *Cont.*

Reference	Centres' practice/protocol for monitoring and data reported	Target blood Phe ranges for age	Blood Phe levels for age<br>Mean ± SD [Median, range]
Sweeney et al., 2012	***Protocol:*** Blood Phe levels were measured using a weekly home-collected filter paper blood spot at a regular time that suited the family, continued as usual, and measured by tandem mass spectrometry. Dietary management aimed for blood Phe levels within the range recommended by the Medical Research Council Working Party on Phenylketonuria (1993).<br><br>***Data:*** Mean, median and range of blood Phe levels at baseline and at 6 mo follow-up.	120-360 µmol/L	**Phase I-total; baseline:**<br>407±145 µmol/L [450 µmol/L; 202-723 µmol/L; N=17]<br>**Phase I-total; 6 mo:**<br>431±179 µmol/L [425 µmol/L; 119-737 µmol/L; N=10]<br><br>**Phase I-Control; before new exchange system**:<br>352±175 µmol/L [334 µmol/L; 67-609 µmol/L; N=8]<br>**Phase I-Control; 6 mo after new exchange system:**<br>306±165 µmol/L [256 µmol/L; 81-553 µmol/L; N=8]<br><br>**Phase II-baseline:**<br>442±137 µmol/L [456 µmol/L; 274-657 µmol/L; N=18]<br>**Phase II-6 mo:**<br>399±149 µmol/L [359 µmol/L; 185-663 µmol/L; N=18]
Thiele et al., 2017	***Protocol:*** To evaluate patients' lifetime metabolic control, their individual mean Phe concentration per year from birth onward to the age of 18 years was calculated retrospectively from medical records. Phe concentrations were measured in dried blood by liquid chromatography/tandem mass spectrometry or by fluorometry.<br><br>***Data:*** Individual mean, SD Phe concentration per year from birth to 18 y of age.	**1-10 y:** 42-240 µmol/l (0.7-4 mg/dl)<br><br>**11-16 y:** 42-900 µmol/l (0.7-15 mg/dl)<br><br>**> 16y:** 42-1200 µmol/l (0.7-20 mg/dl)	Mean dried blood Phe concentrations remained in the age-specific recommended range during the evaluation period;<br>**0-1 y:** 301±153 µmol/L; **1-2 y:** 290±151 µmol/L<br>**2-3 y:** 307±170 µmol/L; **3-4 y:** 320±168 µmol/L<br>**4-5 y:** 341±189 µmol/L; **5-6 y:** 332±197 µmol/L<br>**6-7 y:** 335±191 µmol/L; **7-8 y:** 331±170 µmol/L<br>**8-9 y:** 333±165 µmol/L; **9-10 y:** 361±197 µmol/L<br>**10-11 y:** 367±174 µmol/L; **11-12 y:** 423±89 µmol/L<br>**12-13 y:** 456±224 µmol/L; **13-14 y:** 468±226 µmol/L<br>**14-15 y:** 506±256 µmol/L; **15-16 y:** 541±503 µmol/L<br>**16-17 y:** 579±275 µmol/L; **17-18 y:** 637±270 µmol/L
Trefz et al., 2009	***Protocol:*** Different protocols at each 15 centers.<br><br>***Data:*** Mean, SD of blood Phe levels over prior 6 months (baseline)	≤360 µmol/L	**Over prior 6 mo to BH4 therapy:**<br>**BH4 group:** 314±107 µmol/L [112-474 µmol/L; N=33]<br>**Placebo:** 303±74 µmol/L [176-447 µmol/L; N=12]<br>**Total:** 309 µmol/L (N=45)<br><br>**Final Phe level at 10-wk:**<br>**Placebo:** 461±235 µmol/L (N=12)

Supplementary Table 7. *Cont.*

Reference	Centres' practice/protocol for monitoring and data reported	Target blood Phe ranges for age	Blood Phe levels for age<br>Mean ± SD [Median, range]
van Spronsen et al., 2009	***Protocol:*** The means of Phe concentrations of blood samples collected at the 1 mo, 6 mo, 1, 2, 3, 5, 10 y of age were subsequently used to decide on metabolic control and to include or exclude data on tolerance of the individual patient at that point. Data that were not within the target range of the Phe concentrations were excluded when calculating Phe tolerance.<br><br>***Data:*** Mean Phe tolerance at the seven time points (1 mo, 6 mo, 1, 2, 3, 5, 10 y of age) was used.	200–500 μmol/L	Fluctuations in plasma Phe concentrations were observed in almost all patients under the same prescribed Phe intake n=213.<br>The decrease in the number of patients with Phe concentration within target limits was in line with data of other studies showing that from the ages of 5 to 10 years progressively fewer plasma concentrations are within range.
Wendel et al., 1990	***Protocol:*** Blood specimens were drawn and mailed to laboratory for analysis;<br>**0-1y:** At least one estimation every 2 weeks;<br>**>1y:** One estimation every 4 weeks; at least one quantitative estimation (by fluorometry or automated amino acid analysis) every 2 months;<br>***Data:*** Mean, median, range of blood Phe were calculated over each 6-month period.	2-6 mg/dl	**In low Phe plasma level group:**<br>5.9±0.9 mg/dL [6.1 mg/dL; 5.6-9.4 mg/dl, N=112]<br><br>Plotting the data of the high-level subgroup separately, it became apparent that the means of the median plasma Phe levels of the great majority of the PKU patients were maintained quite well at the designated upper limit of 6 mg/dl.

Abbreviations; PKU, phenylketonuria; Phe, phenylalanine; mo, months; y, year; h, hour; SD, standard deviation; BH4, tetrahydrobiopterin; FU: follow-up; N, number of patients; GMP: glycomacropeptide; AAM: amino acid mixture; IQR: interquartile range; PS, protein substitute; RDA, recommended dietary allowance; FAO, Food and Agriculture Organization of the United Nations; MRC, Medical Research Council; Tyr, tyrosine.
