# Supplementary material for: Phenylalanine Tolerance over Time in Phenylketonuria: A Systematic Review and Meta-Analysis
Source: Nutrients. 2023 Aug 8;15(16):3506. doi: 10.3390/nu15163506 (PMC10458574; doi:10.3390/nu15163506)
Supplement: Supplementary file 1 [file nutrients-15-03506-s001.zip › Supplementary Tables S1-S5_Dietary intakes_06072023.pdf]

**Supplementary Table S1.** Actual dietary intakes of PKU patients in early childhood (0-3 years of age).

| Reference | N | Age | Energy | | Phenylalanine | | Natural protein | | Protein equivalent from protein substitute | | Total protein | |
|---|---|---|---|---|---|---|---|---|---|---|---|---|
| | | | *kcal/day* | *kcal/kg/day* | *mg/day* | *mg/kg/day* | *g/day* | *g/kg/day* | *g/day* | *g/kg/day* | *g/day* | *g/kg/day* |
| Acosta et al., 1998 | | | ***Mean (SD)*** | N/A | ***Mean (SD)*** | ***Mean (SD)*** | N/A | | ***Mean (SD)*** | N/A | ***Mean (SD)*** | ***Mean (SD)*** |
| | 20 | 1 mo | 571 (23) | | 221 (14) | 51 (4) | | | 9.2 (0.75) | | 14.0 (0.7) | 3.2 (0.2) |
| | 33 | 2 mo | 584 (20) | | 250 (13) | 47 (2) | | | 9.2(0.75) | | 14.5 (0.6) | 2.8 (0.1) |
| | 31 | 3 mo | 626 (22) | | 270 (11) | 44 (2) | | | 10.4 (0.60) | | 16.1 (0.6) | 2.6 (0.1) |
| | 34 | 4 mo | 681 (24) | | 264 (15) | 38 (2) | | | 12.5 (0.75) | | 18.1 (0.7) | 2.6 (0.1) |
| | 33 | 5 mo | 718 (26) | | 265 (15) | 34 (2) | | | 14.0 (0.75) | | 19.6 (0.7) | 2.6 (0.1) |
| | 32 | 6 mo | 572 (24) | | 254 (13) | 31 (2) | | | 14.6 (0.75) | | 20.0 (0.7) | 2.4 (0.1) |
| | 35 | Total | 660 (18) | | 255 (10) | 40 (1) | | | 11.9 (0.60) | | 17.3 (0.6) | 2.7 (0.1) |
| Aldámiz-Echevarría et al., 2014 | N/A | 0-2y | N/A | | N/A | ***Median (IQR)*** 28.2 (20.8-36.2) | N/A | ***Median (IQR)*** 0.8 (0.5-1.0) | N/A | ***Median (IQR)*** 1.4 (1.0-1.7) | N/A | ***Median (IQR)*** 2.2 (1.8-2.8) |
| Aldámiz-Echevarría et al., 2015[a] | <u>*BH4*</u> | <u>*BH4*</u> | N/A | | N/A | ***Mean (SD)*** | N/A | ***Mean (SD)*** | N/A | ***Mean (SD)*** | N/A | ***Mean (SD)*** |
| | 22 | 1 y | | | | 37.1 (19.1) | | 1.4 (1.1) | | 0.9 (0.6) | | 2.2 (1.7) |
| | <u>*Diet*</u> | <u>*Diet*</u> | | | | ***Mean (SD)*** | | ***Mean (SD)*** | | ***Mean (SD)*** | | ***Mean (SD)*** |
| | 44 | 1 y | | | | 28.2 (14.1) | | 0.6 (0.2) | | 1.5 (0.5) | | 2.2 (0.5) |
| | | 2 y | | | | 25.8 (17.0) | | 0.6 (0.3) | | 1.6 (0.8) | | 2.1 (0.7) |
| Alm et al., 1986 | 16 | 1 y | N/A | | N/A | ***Mean (SD)*** 43.4 (16.1) ***Median(range)*** 38.5 (30-80) | N/A | | N/A | | N/A | |
| | 17 | 2 y | | | | ***Mean (SD)*** >39.7 (24.8) ***Median(range)*** 27 (16 to >80) | | | | | | |
| Hoeksma et al., 2005 | NP=117 PS=135 TP=133 | | N/A | | N/A | | N/A | ***Mean (SD)*** | N/A | ***Mean (SD)*** | N/A | ***Mean (SD)*** |
| | | 6 mo | | | | | | 1.22 (0.53) | | 1.46 (0.58) | | 2.70 (0.50) |
| | | 1 y | | | | | | 1.03 (0.41) | | 1.28 (0.38) | | 2.36 (0.51) |
| | | 2 y | | | | | | 0.97 (0.46) | | 1.26 (0.34) | | 2.25 (0.56) |
| | | 3 y | | | | | | 0.93 (0.51) | | 1.22 (0.42) | | 2.20 (0.65) |
| | | Total | | | | | | 0.99 (0.34) | | 1.29 (0.28) | | 2.33 (0.42) |

**Supplementary Table S1.** Actual dietary intakes of PKU patients in early childhood (0-3 years of age).

| Reference | N | Age | Energy | | Phenylalanine | | Natural protein | | Protein equivalent from protein substitute | | Total protein | |
|---|---|---|---|---|---|---|---|---|---|---|---|---|
| | | | *kcal/day* | *kcal/kg/day* | *mg/day* | *mg/kg/day* | *g/day* | *g/kg/day* | *g/day* | *g/kg/day* | *g/day* | *g/kg/day* |
| | | | ***Mean (SD)*** | ***Mean (SD)*** | | | ***Mean (SD)*** | ***Mean (SD)*** | ***Mean (SD)*** | ***Mean (SD)*** | ***Mean (SD)*** | ***Mean (SD)*** |
| | 7 to 11 | 4 mo | 686 (146.8) | 95.6 (22.9) | | | 5.6 (1.5) | 0.78 (0.16) | 13.9 (5.0) | 2.0 (0.8) | 17.5 (4.2) | 2.5 (0.6) |
| | 7 to 20 | 5 mo | 735 (160.1) | 100.1 (22.0) | | | 6.6 (1.5) | 0.87 (0.16) | 18.7 (4.9) | 2.6 (0.8) | 21.0 (5.1) | 2.9 (0.7) |
| | 19 | 6 mo | 717 (129.0) | 89.4 (16.2) | | | 5.4 (1.8) | 0.69 (0.23) | 18.9 (5.3) | 2.4 (0.6) | 24.3 (5.4) | 3.1 (0.6) |
| | 20 | 7 mo | 805 (176.5) | 95.0 (18.1) | | | 5.6 (1.8) | 0.66 (0.189 | 21.7 (5.3) | 2.6 (0.5) | 27.3 (5.5) | 3.3 (0.5) |
| | 20 | 8 mo | 871 (217.3) | 97.4 (23.3) | | | 6.3 (1.6) | 0.72 (0.17) | 23.4 (4.6) | 2.7 (0.5) | 29.7 (5.2) | 3.4 (0.6) |
| | 19 to 20 | 9 mo | 958 (226.5) | 99.1 (38.7) | | | 6.3 (1.8) | 0.69 (0.19) | 25.7 (3.5) | 2.8 (0.4) | 32.0 (4.1) | 3.5 (0.4) |
| | 18 to 19 | 10 mo | 1018 (315.8) | 99.9 (38.7) | | | 7.1 (2.1) | 0.75 (0.22) | 26.7 (4.9) | 2.8 (0.4) | 33.8 (6.1) | 3.5 (0.6) |
| | 16 to 17 | 11 mo | 985 (243.0) | 97.0 (22.9) | | | 7.6 (2.6) | 0.77 (0.28) | 27.2 (5.3) | 2.7 (0.5) | 34.9 (6.5) | 3.5 (0.6) |
| | 17 to 19 | 12 mo | 1004 (271.5) | 101.9 (23.0) | | | 7.0 (2.8) | 0.69 (0.30) | 27.0 (5.6) | 2.7 (0.4) | 33.9 (6.6) | 3.3 (0.6) |
| | 18 to 20 | 15 mo | 1176 (246.1) | 102.1 (33.8) | | | 7.9 (2.6) | 0.72 (0.26) | 31.3 (6.7) | 2.9 (0.5) | 39.3 (7.4) | 3.6 (0.6) |
| | 20 | 18 mo | 1184 (306.9) | 100.0 (28.0) | | | 8.0 (2.7) | 0.68 (0.23) | 33.5 (7.1) | 2.8 (0.6) | 41.4 (7.9) | 3.5 (0.7) |
| | 19 | 24 mo | 1321 (369.2) | 100.4 (30.5) | | | 9.0 (2.8) | 0.69 (0.21) | 36.0 (5.4) | 2.7 (0.4) | 45.0 (5.3) | 3.4 (0.4) |
| Evans et al., 2019[b] | | | ***Median (range)*** | ***Median (range)*** | N/A | | ***Median (range)*** | ***Median (range)*** | ***Median (range)*** | ***Median (range)*** | ***Median (range)*** | ***Median (range)*** |
| | 7 to 11 | 4 mo | 661 (369-913) | 98.3 (58-130) | | | 5.2 (4.4-8.9) | 0.70 (0.6-1.1) | 14.0 (5.5-23.9) | 2.1 (0.8-3.7) | 16.9 (10.2-23.9) | 2.5 (1.5-3.7) |
| | 7 to 20 | 5 mo | 689 (482-1083) | 94.6 (64-141) | | | 6.1 (5.6-10.0) | 0.83 (0.7-1.2) | 18.0 (6.1-28.4) | 2.5 (0.8-4.2) | 21.9 (12.0-32.5) | 2.9 (1.5-4.2) |
| | 19 | 6 mo | 699 (529-1021) | 88.1 (62-133) | | | 5.2 (3.2-9.8) | 0.65 (0.4-1.3) | 19.6 (10.9-28.1) | 2.4 (1.3-3.5) | 24.1 (16.5-37.1) | 3.0 (1.8-4.1) |
| | 20 | 7 mo | 784 (583-1187) | 93.1 (62-127) | | | 5.1 (3.2-10.3) | 0.62 (0.4-1.1) | 21.0 (11.5-30.3) | 2.7 (1.4-3.4) | 26.1 (20.5-39.9) | 3.3 (2.3-4.3) |
| | 20 | 8 mo | 813 (644-1376) | 90.4 (59-146) | | | 6.1 (4.5-11.7) | 0.66 (0.5-1.2) | 23.0 (15.0-32.2) | 2.7 (1.6-3.3) | 28.8 (21.3-43.9) | 3.4 (2.3-4.5) |
| | 19 to 20 | 9 mo | 928 (593-1502) | 101.5 (11-153) | | | 6.0 (4.2-10.7) | 0.61 (0.5-1.1) | 25.1 (19.8-31.8) | 2.9 (2.2-3.3) | 30.2 (27.1-42.5) | 3.5 (2.7-4.4) |
| | 18 to 19 | 10 mo | 1003 (611-1910) | 98.5 (60-172) | | | 7.2 (4.2-13.7) | 0.75 (0.4-1.4) | 26.6 (17.2-35.1) | 2.9 (2.0-3.3) | 32.7 (22.3-45.5) | 3.6 (2.5-4.5) |
| | 16 to 17 | 11 mo | 934 (703-1613) | 98.3 (61-138) | | | 7.0 (4.3-15.5) | 0.71 (0.4-1.6) | 26.5 (18.5-38.0) | 2.7 (2.0-3.8) | 32.8 (28.4-53.5) | 3.4 (2.7-5.4) |
| | 17 to 19 | 12 mo | 952 (621-1756) | 106.2 (62-150) | | | 6.1 (4.3-15.5) | 0.65 (0.4-1.5) | 27.0 (18.6-37.7) | 2.7 (2.0-3.7) | 33.3 (24.7-53.2) | 3.4 (2.6-5.2) |
| | 18 to 20 | 15 mo | 1132 (768-1644) | 103.6 (72-153) | | | 7.5 (3.0-15.5) | 0.69 (0.3-1.4) | 31.8 (20.2-43.7) | 2.8 (2.1-4.0) | 38.8 (26.8-59.2) | 3.4 (2.7-5.4) |
| | 20 | 18 mo | 1161 (596-2014) | 96.8 (55-167) | | | 7.6 (5.3-17.5) | 0.63 (0.4-1.4) | 32.1 (24.6-51.7) | 2.7 (1.9-4.2) | 40.0 (33.5-61.1) | 3.3 (2.3-5.0) |
| | 19 | 24 mo | 1667 (682-1912) | 90.3 (54-166) | | | 8.4 (6.0-18.0) | 0.66 (0.4-1.3) | 36.4 (25.0-46.2) | 2.7 (1.9-3.5) | 43.4 (34.9-56.8) | 3.4 (2.3-4.0) |
| | | | | | | | | | | | | ***Median (range)*** |
| | 15 | 4 mo | | | | | | | | | | 2.6 (2.1-3.1) |
| | 15 | 6 mo | | | | | | | | | | 2.1 (1.7-2.8) |
| Kindt et al., 1983 | 16 | 9 mo | N/A | | N/A | | N/A | | N/A | | N/A | 2.0 (1.6-2.6) |
| | 16 | 12 mo | | | | | | | | | | 1.8 (1.5-2.7) |
| | 16 | 1.5 y | | | | | | | | | | 1.7 (1.3-2.5) |
| | 16 | 2 y | | | | | | | | | | 1.6 (1.2-2.4) |
| | 11 | 2.5 y | | | | | | | | | | 1.6 (1.2-2.3) |

**Supplementary Table S1.** Actual dietary intakes of PKU patients in early childhood (0-3 years of age).

| Reference | N | Age | Energy | | Phenylalanine | | Natural protein | | Protein equivalent from protein substitute | | Total protein | |
|---|---|---|---|---|---|---|---|---|---|---|---|---|
| | | | *kcal/day* | *kcal/kg/day* | *mg/day* | *mg/kg/day* | *g/day* | *g/kg/day* | *g/day* | *g/kg/day* | *g/day* | *g/kg/day* |
| | 11 | 3 y | | | | | | | | | | 1.6 (1.2-2.1) |
| Ponzone et al., 2008 | 22 | 2 mo | N/A | | ***Mean (SD)***<br>284.1 (53.8)<br>***Median (range)***<br>280 (220-390) | N/A | N/A | | N/A | | N/A | |
| Rohde et al., 2015 | | | N/A | | ***Mean (SD)*** | ***Mean (SD)*** | ***Mean (SD)*** | ***Mean (SD)*** | N/A | | N/A | |
| | 20 | 1 y | | | 267.0 (59.8) | 25.0 (5.1) | 6.7 (1.5) | 0.6 (0.1) | | | | |
| | 13 | 2 y | | | 311.9 (166.9) | 22.6 (10.8) | 7.8 (4.2) | 0.6 (0.3) | | | | |
| | 13 | 3 y | | | 288.8 (96.4) | 19.4 (5.5) | 7.2 (2.4) | 0.5 (0.1) | | | | |
| | 46 | 0-3 y | | | 285.9 (108.5) | 22.7 (7.4) | 7.1 (2.7) | 0.6 (0.2) | | | | |
| | | | | | ***Median (range)*** | ***Median (range)*** | ***Median (range)*** | ***Median (range)*** | | | | |
| | 20 | 1 y | | | 250 (190-450) | 24.6 (16.9-33.7) | 6.3 (4.8-11.3) | 0.6 (0.4-0.8) | | | | |
| | 13 | 2 y | | | 270 (160-800) | 19.2 (12.9-54.4) | 6.8 (4.0-20.0) | 0.5 (0.3-1.4) | | | | |
| | 13 | 3 y | | | 260 (180-550) | 18.2 (13.4-34.4) | 6.5 (4.5-13.8) | 0.5 (0.3-0.9) | | | | |
| | 46 | 0-3 y | | | 255 (160-800) | 20.4 (12.9-54.4) | 6.4 (4.0-20.0) | 0.5 (0.3-1.4) | | | | |
| Thiele et al., 2017 | | | N/A | | N/A | | N/A | ***Mean (SD)*** | N/A | ***Mean (SD)*** | N/A | ***Mean (SD)*** |
| | 82 | 1 y | | | | | | 0.7 (0.2) | | 1.6 (0.4) | | 2.3 (0.4) |
| | 90 | 2 y | | | | | | 0.5 (0.1) | | 1.6 (0.4) | | 2.1 (0.4) |
| | 83 | 3 y | | | | | | 0.5 (0.1) | | 1.6 (0.7) | | 2.0 (0.5) |

Abbreviations: PKU, phenylketonuria; N, number of patients in the age group; mo, months; y, years; SD, standard deviation; N/A, not available; IQR, inter quartile range; BH4, tetrahydrobiopterin; NP, natural protein; TP, total protein; PS, protein equivalent from protein substitute.

[a] Data of BH4 group refers to the baseline intakes of patients who were treated with BH4; diet group includes the baseline and follow-up data of patients who were treated with a phenylalanine restricted diet.

[b] The sample size was shown as a range where the number of included patients was different for energy and protein intakes (e.g. due to missing data, loss to follow-up, etc.)

**Supplementary Table S2.** Actual dietary intakes of PKU patients in late childhood (4-10 years of age).

| Reference | N | Age | Dietary intakes | | | | | | | | | |
|---|---|---|---|---|---|---|---|---|---|---|---|---|
| | | | **Energy** | | **Phenylalanine** | | **Natural protein** | | **Protein equivalent from protein substitute** | | **Total protein** | |
| | | | *kcal/day* | *kcal/kg/day* | *mg/day* | *mg/kg/day* | *g/day* | *g/kg/day* | *g/day* | *g/kg/day* | *g/day* | *g/kg/day* |
| Aldámiz-Echevarría et al., 2013[a] | 36 | *BH4*$_1$<br>5 y | N/A | | N/A | ***Median (IQR)***<br>29.9 (18.3-52.3) | N/A | | N/A | ***Median (IQR)***<br>0.9 (0.7-1.2) | N/A | ***Median (IQR)***<br>1.8 (1.0-3.6) |
| | 10 | *BH4*$_2$<br>5 y | | | | ***Median (IQR)***<br>30.8 (24.6-54.8) | | | | ***Median (IQR)***<br>0.7 (0.5-1.2) | | ***Median (IQR)***<br>1.7 (1.3-2.2) |
| | 72 | *Diet*$_1$<br>5 y<br>7 y | | | | ***Median (IQR)***<br>19.2 (9.3-31.6)<br>12.9 (7.9-20.3) | | | | ***Median (IQR)***<br>1.3 (1.0-1.8)<br>1.2 (0.9-1.8) | | ***Median (IQR)***<br>2.0 (1.3-2.4)<br>1.4 (1.0-2.4) |
| | 20 | *Diet*$_2$<br>5 y<br>10 y | | | | ***Median (IQR)***<br>9.9 (8.2-21.8)<br>10.9 (8.8-13.7) | | | | ***Median (IQR)***<br>1.5 (1.0-1.7)<br>1.1 (0.8-1.3) | | ***Median (IQR)***<br>2.0 (1.6-2.4)<br>1.6 (1.2-1.9) |
| Aldámiz-Echevarría et al., 2014 | N/A | 2-9 y | N/A | | N/A | ***Median (IQR)***<br>15.5 (10.9-20.4) | N/A | | N/A | ***Median (IQR)***<br>1.3 (1.0-1.7) | N/A | ***Median (IQR)***<br>1.8 (1.4-2.3) |
| Daly et al., 2019 | 18 | 10 y | N/A | | N/A | | ***Median (range)***<br>5 (3-30) | N/A | ***Median (range)***<br>60 (40-80) | N/A | N/A | |
| Dobbelaere et al., 2003[b] | 20<br>Phe=15 | 4.5 y | ***Mean***<br>1264 | ***Mean (SD)***<br>86 (20)<br>***Range***<br>61-128 | ***Mean (SD)***<br>391 (182.8)<br>***Median (range)***<br>348 (204-768) | ***Mean (SD)***<br>27 (8.2)<br>***Median (range)***<br>26.6 (18.5-42.4) | N/A | | N/A | | ***Mean***<br>24.5 | ***Mean (SD)***<br>1.67 (0.23)<br>***Range***<br>1.2-2.1 |
| Evans et al., 2017 | 32 | 9 y | N/A | | N/A | | N/A | ***Median (range)***<br>0.5 (0.18-0.8) | N/A | ***Median (range)***<br>1.54 (0.8-2.7) | N/A | ***Median (range)***<br>2.05 (1.0-3.5) |
| Giovannini et al., 2014 | 55 | 9 y | ***Mean (SD)***<br>1762 (399) | ***Mean (SD)***<br>60 (4.3) | ***Mean***<br>398 | N/A | ***Mean***<br>13.2 | ***Mean (SD)***<br>0.5 (0.3) | N/A | ***Mean (SD)***<br>0.15 (0.47) | ***Mean***<br>54.2 | ***Mean***<br>1.95 |

**Supplementary Table S2.** Actual dietary intakes of PKU patients in late childhood (4-10 years of age).

| Reference | N | Age | Dietary intakes | | | | | | | | | |
|---|---|---|---|---|---|---|---|---|---|---|---|---|
| | | | **Energy** | | **Phenylalanine** | | **Natural protein** | | **Protein equivalent from protein substitute** | | **Total protein** | |
| | | | *kcal/day* | *kcal/kg/day* | *mg/day* | *mg/kg/day* | *g/day* | *g/kg/day* | *g/day* | *g/kg/day* | *g/day* | *g/kg/day* |
| Gökmen-Özel et al., 2011 | 14 | 6 y | ***Median (range)***<br>1728 (857-3781) | ***Median (range)***<br>72 (36-160) | ***Median (range)***<br>390 (190-1310) | ***Median (range)***<br>17.1 (8-55.5) | ***Median (range)***<br>7.8 (3.8-26.2) | ***Median (range)***<br>0.32 (0.2-1.1) | ***Median (range)***<br>58.1 (37.8-74.5) | ***Median (range)***<br>2.4 (1.35-3.0) | ***Median (range)***<br>66.2 (41.6-100.7) | ***Median (range)***<br>2.78 (1.55-3.99) |
| Huemer et al., 2007 | 34 | 9-10y | N/A | | N/A | | N/A | ***Mean (SD)***<br>0.3 (0.2)<br>***Median (range)***<br>0.24 (0.1-1.1) | N/A | ***Mean (SD)***<br>0.9 (0.2)<br>***Median (range)***<br>0.84 (0.6-1.4) | ***Mean (SD)***<br>33.7 (10.3)<br>***Median (range)***<br>32.5 (11.3-32.5) | ***Mean (SD)***<br>1.2 (0.3)<br>***Median (range)***<br>1.1 (0.8-2.4) |
| Kindt et al., 1983 | 10 | 3.5 y | N/A | | N/A | | N/A | | N/A | | N/A | ***Median (range)***<br>1.5 (1.23-2.0) |
| | 7 | 4 y | | | | | | | | | | 1.7 (1.5-1.9) |
| | 7 | 5 y | | | | | | | | | | 1.6 (1.36-1.7) |
| | 3 | 6 y | | | | | | | | | | 1.5 (1.5-1.5) |
| MacDonald et al., 2006 | 25 | 6 y | N/A | | ***Mean (SD)***<br>485 (180)<br>***Median (range)***<br>450 (250-1050) | N/A | ***Mean (SD)***<br>9.7 (3.6)<br>***Median (range)***<br>9 (5-21) | N/A | ***Mean (SD)***<br>59.4 (9.3)<br>***Median (range)***<br>60 (40-80) | N/A | N/A | |
| Rocha et al., 2012 & 2013 | | | N/A | | N/A | | ***Mean (SD)*** | ***Mean (SD)*** | ***Mean (SD)*** | ***Mean (SD)*** | ***Mean (SD)*** | ***Mean (SD)*** |
| | 2 | 4 y | | | | | 21.30 (13.15) | 1.13 (0.57) | 24.51 (0.72) | 1.36 (0.22) | 45.81 (12.43) | 2.49 (0.34) |
| | 4 | 5 y | | | | | 13.73 (4.89) | 0.69 (0.17) | 30.18 (5.69) | 1.58 (0.47) | 43.91 (6.18) | 2.27 (0.47) |
| | 2 | 6 y | | | | | 31.20 (8.49) | 1.15 (0.57) | 33.80 (15.83) | 1.13 (0.27) | 65.00 (7.35) | 2.28 (0.30) |
| | 6 | 7 y | | | | | 25.02 (11.22) | 0.94 (0.32) | 25.52 (6.38) | 1.04 (0.41) | 50.53 (7.05) | 1.99 (0.29) |
| | 5 | 8 y | | | | | 27.14 (13.07) | 1.18 (0.71) | 34.50 (9.23) | 1.39 (0.26) | 61.64 (7.71) | 2.57 (0.57) |
| | 4 | 9 y | | | | | 24.60 (4.98) | 0.93 (0.47) | 27.13 (11.45) | 0.95 (0.32) | 51.73 (10.77) | 1.88 (0.58) |
| | 7 | 10 y | | | | | 27.64 (12.08) | 0.88 (0.42) | 42.30 (7.78) | 1.34 (0.27) | 69.94 (8.74) | 2.22 (0.34) |
| | 30 | 4-10 y | | | | | 24.87 (10.55) | 1.00 (0.49) | 31.61 (10.52) | 1.24 (0.37) | 56.49 (12.16) | 2.24 (0.47) |

**Supplementary Table S2.** Actual dietary intakes of PKU patients in late childhood (4-10 years of age).

| Reference | N | Age | Dietary intakes | | | | | | | | | |
|---|---|---|---|---|---|---|---|---|---|---|---|---|
| | | | **Energy** | | **Phenylalanine** | | **Natural protein** | | **Protein equivalent from protein substitute** | | **Total protein** | |
| | | | *kcal/day* | *kcal/kg/day* | *mg/day* | *mg/kg/day* | *g/day* | *g/kg/day* | *g/day* | *g/kg/day* | *g/day* | *g/kg/day* |
| | | | | | | | ***Median (range)*** | ***Median (range)*** | ***Median (range)*** | ***Median (range)*** | ***Median (range)*** | ***Median (range)*** |
| | 2 | 4 y: | | | | | 21.3 (12.0-30.6) | 1.13 (0.73-1.53) | 24.5 (24.0-25.0) | 1.36 (1.44-1.82) | 45.8 (37-54.6) | 2.49 (2.24-2.73) |
| | 4 | 5 y: | | | | | 11.6 (10.7-21.0) | 0.68 (0.49-0.91) | 30.4 (24.0-36.0) | 1.44 (1.40-2.70) | 46.6 (34.7-47.8) | 2.12 (1.88-2.94) |
| | 2 | 6 y: | | | | | 31.2 (25.2-37.2) | 1.15 (0.74-1.55) | 33.8 (22.6-45.0) | 1.13 (1.13-1.59) | 65.0 (59.8-70.2) | 2.28 (2.06-2.49) |
| | 6 | 7 y: | | | | | 21.9 (14.2-38.8) | 0.79 (0.68-1.36) | 25.5 (18.0-36.0) | 1.03 (0.75-1.80) | 51.9 (41.6-56.9) | 2.01 (1.48-2.35) |
| | 5 | 8 y: | | | | | 26.9 (14.8-45.9) | 1.12 (0.51-2.24) | 34.5 (20.0-45.0) | 1.50 (1.17-1.95) | 65.9 (48.8-67.5) | 2.75 (1.91-3.21) |
| | 4 | 9 y: | | | | | 23.0 (20.7-31.8) | 0.71 (0.67-1.63) | 25.5 (17.9-42.1) | 0.97 (0.65-1.58) | 50.5 (39.9-66.0) | 1.86 (1.21-2.58) |
| | 7 | 10 y: | | | | | 20.8 (14.6-44.1) | 0.62 (0.55-1.48) | 41.7 (30.0-55.0) | 1.28 (1.24-2.05) | 72.9 (55.6-82.6) | 2.22 (1.81-2.75) |
| | 30 | 4-10 y: | | | | | 22.0 (10.7-45.9) | 0.74 (0.49-2.24) | 30.0 (12.5-55.0) | 1.32 (0.54-2.25) | 56.5 (34.7-82.6) | 2.1 (1.21-3.21) |
| Rohde et al., 2012 & 2014a | 14 | *Initial* 6 y | ***Mean (SD)*** 1384 (231) | N/A | ***Mean (SD)*** 335 (72) ***Range*** 270-480 | N/A | N/A | | N/A | | ***Mean (SD)*** 41 (15) | N/A |
| | 19 | *FU1* 5.5 y | N/A | | ***Mean (SD)*** 422 (95) ***Median (range)*** 408 (282-713) | | | | | | ***Mean (SD)*** 44 (16) ***Median (range)*** 42 (22-83) | |
| | 16 | *FU2* 6 y | N/A | | ***Mean (SD)*** 413 (101) ***Median (range)*** 410 (259-695) | | | | | | ***Mean (SD)*** 46 (16) ***Median (range)*** 45 (27-78) | |
| Rohde et al., 2015 | | | N/A | | ***Mean (SD)*** | ***Mean (SD)*** | ***Mean (SD)*** | ***Mean (SD)*** | N/A | | N/A | |
| | 14 | 4 y | | | 307.5 (68.8) | 19.0 (4.3) | 7.7 (1.7) | 0.5 (1.1) | | | | |
| | 9 | 5 y | | | 328.3 (87.3) | 17.6 (6.2) | 8.2 (2.2) | 0.4 (0.2) | | | | |
| | 9 | 6 y | | | 368.9 (186.9) | 15.8 (8.3) | 9.2 (4.7) | 0.4 (0.2) | | | | |
| | 5 | 7 y | | | 304.0 (61.9) | 13.2 (3.7) | 7.6 (1.5) | 0.3 (0.1) | | | | |
| | 13 | 8 y | | | 405.0 (150.0) | 14.6 (6.9) | 10.1 (3.7) | 0.4 (0.2) | | | | |
| | 12 | 9 y | | | 360.4 (94.1) | 11.4 (2.8) | 9.0 (2.4) | 0.3 (0.1) | | | | |
| | 11 | 10 y | | | 410.0 (133.5) | 11.8 (3.4) | 10.3 (3.3) | 0.3 (0.1) | | | | |

**Supplementary Table S2.** Actual dietary intakes of PKU patients in late childhood (4-10 years of age).

| Reference | N | Age | Dietary intakes | | | | | | | | | |
|---|---|---|---|---|---|---|---|---|---|---|---|---|
| | | | **Energy** | | **Phenylalanine** | | **Natural protein** | | **Protein equivalent from protein substitute** | | **Total protein** | |
| | | | *kcal/day* | *kcal/kg/day* | *mg/day* | *mg/kg/day* | *g/day* | *g/kg/day* | *g/day* | *g/kg/day* | *g/day* | *g/kg/day* |
| | 73 | 4-10 y | | | 358.9 (122.5) | 14.9 (5.9) | 9.0 (3.1) | 0.4 (0.2) | | | | |
| | | | | | ***Median (range)*** | ***Median (range)*** | ***Median (range)*** | ***Median (range)*** | | | | |
| | 14 | 4 y | | | 300 (220-450) | 18.6 (12.9-27.8) | 7.5 (5.5-11.3) | 0.5 (0.3-0.7) | | | | |
| | 9 | 5 y | | | 300 (240-540) | 15.8 (12.2-32.7) | 7.5 (6.0-13.5) | 0.4 (0.3-0.8) | | | | |
| | 9 | 6 y | | | 310 (180-700) | 12.0 (7.4-33.2) | 7.8 (4.5-17.5) | 0.3 (0.2-0.8) | | | | |
| | 5 | 7 y | | | 320 (200-350) | 15.2 (7.7-16.4) | 8.0 (5.0-8.8) | 0.4 (0.2-0.4) | | | | |
| | 13 | 8 y | | | 350 (240-800) | 11.9 (8.8-34.2) | 8.8 (6.0-20.0) | 0.3 (0.2-0.9) | | | | |
| | 12 | 9 y | | | 305 (290-600) | 10.4 (7.6-15.9) | 7.6 (7.3-15.0) | 0.3 (0.2-0.4) | | | | |
| | 11 | 10 y | | | 380 (250-750) | 11.2 (7.4-18.0) | 9.5 (6.3-18.8) | 0.3 (0.2-0.5) | | | | |
| | 73 | 4-10 y | | | 320 (180-800) | 14.0 (7.4-34.2) | 8.0 (4.5-20.0) | 0.4 (0.2-0.9) | | | | |
| Schulpis et al., 2013 | 30 | 5 y | ***Mean (SD)*** 2020 (400) | N/A | N/A | | ***Mean (SD)*** 9 (1.2) | N/A | N/A | | ***Mean (SD)*** 70 (18) | N/A |
| Thiele et al., 2017 | | | N/A | | N/A | | N/A | ***Mean (SD)*** | N/A | ***Mean (SD)*** | N/A | ***Mean (SD)*** |
| | 86 | 4 y | | | | | | 0.4 (0.1) | | 1.5 (0.4) | | 1.9 (0.4) |
| | 83 | 5 y | | | | | | 0.4 (0.1) | | 1.4 (0.3) | | 1.8 (0.4) |
| | 86 | 6 y | | | | | | 0.3 (0.1) | | 1.4 (0.3) | | 1.7 (0.4) |
| | 82 | 7 y | | | | | | 0.3 (0.1) | | 1.3 (0.3) | | 1.6 (0.3) |
| | 78 | 8 y | | | | | | 0.3 (0.1) | | 1.2 (0.2) | | 1.5 (0.3) |
| | 79 | 9 y | | | | | | 0.3 (0.1) | | 1.1 (0.3) | | 1.4 (0.3) |
| | 75 | 10 y | | | | | | 0.2 (0.1) | | 1.0 (0.3) | | 1.2 (0.3) |
| Trefz et al., 2009 | 39 | 7 y | N/A | | ***Mean (SD)*** 480.5 (43.1) | ***Mean (SD)*** 16.6 (0.4) | N/A | | N/A | | N/A | |

Abbreviations: PKU, phenylketonuria; N, number of patients in the age group; y, years; SD, standard deviation; N/A, not available; IQR, inter quartile range; BH4, tetrahydrobiopterin; Phe, phenylalanine; FU, follow-up.

[a] $BH4_1$ and $BH4_2$ groups refer to baseline data (i.e. pre-BH4 treatment) of patients who were followed for 2 y and 5 y, respectively. $Diet_1$ and $Diet_2$ groups refer to baseline and follow up data (at 2 y and 5y) of patients who were treated with Phe-restricted diet only.

[b] Data on dietary Phe intake was available for 15 patients.

**Supplementary Table S3.** Actual dietary intakes of PKU patients during adolescence (11-18 years of age).

| Reference | N | Age | Dietary intakes | | | | | | | | |
|---|---|---|---|---|---|---|---|---|---|---|---|
| | | | Energy | Phenylalanine | | Natural protein | | Protein equivalent from protein substitute | | Total protein | |
| | | | *kcal/day* | *mg/day* | *mg/kg/day* | *g/day* | *g/kg/day* | *g/day* | *g/kg/day* | *g/day* | *g/kg/day* |
| Aldámiz-Echevarría et al., 2014 | N/A | 9-12 y<br>12-18 y | N/A | N/A | ***Median (IQR)***<br>8.6 (7.0-11.3)<br>7.6 (5.4-10.3) | N/A | ***Median (IQR)***<br>0.3 (0.2-0.5)<br>0.3 (0.2-0.5) | N/A | ***Median (IQR)***<br>1.2 (0.9-1.4)<br>1.0 (0.7-1.3) | N/A | ***Median (IQR)***<br>1.2 (1.0-1.7)<br>1.1 (1.0-1.4) |
| Alm et al., 1986 | 18 | 12y | N/A | N/A | ***Mean (SD)***<br>>47.3 (30.1)<br>***Median (range)***<br>25 (20 to >80) | N/A | | N/A | | N/A | |
| Daly et al., 2017[a] | 21<br>21<br>21 | 11 y<br>11.5 y<br>Total | ***Range***<br>1624-1689<br>1630-1690<br>N/A | N/A | | ***Range***<br>9.0-10.8<br>9.8-10<br>***Median (range)***<br>5 (3-30) | N/A | N/A<br>N/A<br>***Median (range)***<br>60 (60-80) | N/A | N/A | |
| Pinto et al., 2019 | 40 | 17 y | ***Median (IQR)***<br>2365<br>(2202-2575)<br>43.5<br>kcal/kg/day | ***Median***<br>1900 | ***Median (IQR)***<br>35 (20.9-53.1) | ***Median (IQR)***<br>35 (24-70.3) | ***Median (IQR)***<br>0.7 (0.42-1.06) | ***Median (IQR)***<br>44.8 (24-55) | ***Median (IQR)***<br>0.82 (0.48-1.04) | N/A | |
| Rocha et al., 2012 & 2013 | | | N/A | N/A | | ***Mean (SD)*** | ***Mean (SD)*** | ***Mean (SD)*** | ***Mean (SD)*** | ***Mean (SD)*** | ***Mean (SD)*** |
| | 8 | 11 y | | | | 31.41 (13.29) | 0.75 (0.33) | 52.45 (16.04) | 1.27 (0.46) | 83.87 (18.0) | 2.03 (0.60) |
| | 2 | 12 y | | | | 19.35 (3.89) | 0.34 (0.01) | 66.61 (11.61) | 1.17 (0.07) | 85.96 (15.5) | 1.50 (0.08) |
| | 3 | 13 y | | | | 30.47 (15.10) | 0.72 (0.46) | 55.94 (7.70) | 1.22 (0.17) | 86.40 (8.37) | 1.94 (0.57) |
| | 1 | 14 y | | | | 37.80 (0.00) | 0.65 (0.00) | 38.51 (0.00) | 0.66 (0.00) | 76.31 (0.00) | 1.30 (0.00) |
| | 6 | 15 y | | | | 22.32 (6.16) | 0.41 (0.10) | 53.31 (19.49) | 0.99 (0.37) | 75.63 (16.9) | 1.39 (0.30) |
| | 7 | 16 y | | | | 29.15 (14.30) | 0.46 (0.23) | 54.44 (19.45) | 0.84 (0.27) | 83.59 (20.9) | 1.30 (0.31) |
| | 2 | 17 y | | | | 22.60 (3.82) | 0.41 (0.05) | 71.10 (12.40) | 1.30 (0.16) | 93.70 (16.2) | 1.71 (0.22) |
| | 3 | 18 y | | | | 18.77 (2.87) | 0.29 (0.03) | 65.62 (3.44) | 1.03 (0.08) | 84.38 (3.31) | 1.32 (0.05) |
| | 32 | 11-18 y | | | | 26.83 (11.34) | 0.53 (0.28) | 56.23 (15.89) | 1.07 (0.35) | 83.06 (15.7) | 1.60 (0.49) |
| | | | | | | ***Median (range)*** | ***Median(range)*** | ***Median (range)*** | ***Median(range)*** | ***Median (range)*** | ***Median (range)*** |
| | 8 | 11 y | | | | 28.0 (16.5-57.4) | 0.69 (0.40-1.30) | 53.81 (19.6-74.3) | 1.24 (0.49-2.17) | 80.6 (54.7-113.4) | 1.91 (1.14-3.00) |
| | 2 | 12 y | | | | 19.4 (16.6-22.1) | 0.34 (0.33-0.35) | 66.61 (58.4-74.8) | 1.17 (1.34-1.46) | 86.0 (75.0-96.9) | 1.50 (1.45-1.56) |
| | 3 | 13 y | | | | 26.0 (18.1-47.3) | 0.65 (0.29-1.21) | 55.00 (48.7-64.1) | 1.25 (1.25-1.65) | 82.2 (81.0-96.1) | 2.03 (1.34-2.46) |

**Supplementary Table S3.** Actual dietary intakes of PKU patients during adolescence (11-18 years of age).

| Reference | N | Age | Dietary intakes | | | | | | | | |
|---|---|---|---|---|---|---|---|---|---|---|---|
| | | | **Energy** | **Phenylalanine** | | **Natural protein** | | **Protein equivalent from protein substitute** | | **Total protein** | |
| | | | *kcal/day* | *mg/day* | *mg/kg/day* | *g/day* | *g/kg/day* | *g/day* | *g/kg/day* | *g/day* | *g/kg/day* |
| | 1 | 14 y | | | | 37.8 (-) | 0.65 (-) | 38.51 (-) | 0.66 (-) | 76.3 (-) | 1.30 (-) |
| | 6 | 15 y | | | | 23.5 (14.3-30.3) | 0.39 (0.29-0.55) | 59.03 (14.8-68.7) | 1.08 (0.32-1.58) | 78.6 (45.1-92.6) | 1.45 (0.81-1.64) |
| | 7 | 16 y | | | | 26.7 (15.8-56.2) | 0.45 (0.21-0.86) | 62.79 (17.7-69.7) | 0.92 (0.35-1.25) | 85.7 (55.1-122.3) | 1.22 (0.91-1.88) |
| | 2 | 17 y | | | | 22.6 (19.9-25.3) | 0.41 (0.37-0.45) | 71.10 (62.3-79.8) | 1.30 (1.42-1.70) | 93.7 (82.2-105.2) | 1.71 (1.56-1.87) |
| | 3 | 18 y | | | | 18.4 (16.1-21.8) | 0.28 (0.27-0.33) | 65.00 (62.5-69.3) | 1.06 (1.12-1.30) | 84.3 (81.1-87.7) | 1.34 (1.26-1.35) |
| | 32 | 11-18 y | | | | 23.45 (14.3-57.4) | 0.45 (0.21-1.30) | 61.67 (14.8-79.9) | 1.07 (0.27-1.81) | 82.2 (45.1-122.3) | 1.47 (0.81-3.00) |
| | | | | ***Mean (SD)*** | ***Mean (SD)*** | ***Mean (SD)*** | ***Mean (SD)*** | | | | |
| | 4 | 11 y | | 350.0 (147.2) | 9.07 (2.20) | 8.75 (3.68) | 0.23 (0.05) | | | | |
| | 7 | 12 y | | 453.6 (148.7) | 8.82 (2.86) | 11.34 (3.72) | 0.22 80.07) | | | | |
| | 11 | 13 y | | 387.3 (164.4) | 10.8 (5.36) | 9.68 (4.11) | 0.27 (0.13) | | | | |
| | 2 | 14 y | | 595.0 (219.2) | 11.13 (1.10) | 14.88 (5.48) | 0.28 (0.03) | | | | |
| | 6 | 15 y | | 501.7 (165.6) | 7.92 (2.13) | 12.54 (4.14) | 0.20 (0.05) | | | | |
| | 30 | 11-15 y | | 434.5 (164.8) | 9.56 (3.80) | 10.86 (4.12) | 0.24 (0.09) | | | | |
| Rohde et al., 2015 | | | N/A | | | | | N/A | | N/A | |
| | | | | ***Median (range)*** | ***Median (range)*** | ***Median (range)*** | ***Median(range)*** | | | | |
| | 4 | 11 y | | 325 (200-550) | 9.87 (5.83-10.71) | 8.13 (5.00-13.75) | 0.25 (0.15-0.27) | | | | |
| | 7 | 12 y | | 450 (300-750) | 8.57 (5.18-12.72) | 11.25 (7.5-18.75) | 0.21 (0.13-0.32) | | | | |
| | 11 | 13 y | | 320 (190-650) | 9.47 (4.09-20.97) | 8.00 (4.75-16.25) | 0.24 (0.10-0.52) | | | | |
| | 2 | 14 y | | 595 (440-750) | 11.1 (10.35-11.9) | 14.9 (11.0-18.75) | 0.28 (0.26-0.30) | | | | |
| | 6 | 15 y | | 450 (350-750) | 7.22 (6.25-11.95) | 11.3 (8.75-18.75) | 0.18 (0.16-0.30) | | | | |
| | 30 | 11-15 y | | 420 (190-750) | 9.03 (4.09-20.97) | 10.5 (4.75-18.75) | 0.23 (0.10-0.52) | | | | |
| | | | | | | | ***Mean (SD)*** | | ***Mean (SD)*** | | ***Mean (SD)*** |
| | 70 | 11 y | | | | | 0.2 (0.1) | | 1.0 (0.3) | | 1.2 (0.3) |
| | 70 | 12 y | | | | | 0.2 (0.1) | | 1.0 (0.3) | | 1.1 (0.3) |
| | 67 | 13 y | | | | | 0.2 (0.1) | | 0.9 (0.2) | | 1.1 (0.3) |
| Thiele et al., 2017 | 65 | 14 y | N/A | N/A | | N/A | 0.2 (0.1) | N/A | 0.9 (0.2) | N/A | 1.1 (0.2) |
| | 58 | 15 y | | | | | 0.2 (0.1) | | 0.9 (0.2) | | 1.0 (0.2) |
| | 53 | 16 y | | | | | 0.2 (0.1) | | 0.9 (0.2) | | 1.0 (0.2) |
| | 53 | 17 y | | | | | 0.2 (0.1) | | 0.8 (0.1) | | 1.0 (0.2) |
| | 47 | 18 y | | | | | 0.2 (0.1) | | 0.8 (0.1) | | 1.0 (0.1) |

Abbreviations: PKU, phenylketonuria; N, number of patients in the age group; y, years; SD, standard deviation; N/A, not available; IQR, inter quartile range.

[a] In the original article, there were two study groups based on the type of protein substitute used; 1) GMP group prescribed with glycomacropeptides ; 2) AAM group using amino acid mixtures as the protein substitute. Range on energy (kcal/day) and natural protein intakes refer to the mean range of intakes of both groups.

**Supplementary Table S4.** Actual dietary intakes of adult PKU patients (>18 years of age).

| Reference | N | Age | Dietary Intakes | | | | | | | | | |
|---|---|---|---|---|---|---|---|---|---|---|---|---|
| | | | Energy | | Phenylalanine | | Natural protein | | Protein equivalent from protein substitute | | Total protein | |
| | | | *kcal/day* | *kcal/kg/day* | mg/day | mg/kg/day | *g/day* | *g/kg/day* | *g/day* | *g/kg/day* | *g/day* | *g/kg/day* |
| Green et al., 2019 | 16 | 29.5 y | ***Mean (SD)*** 1813 (445) | ***Mean*** 25.5 | N/A | | ***Mean (SD)*** 18.3 (7.7) | ***Mean*** 0.3 | ***Mean (SD)*** 58.6 (10.2) | ***Mean*** 0.8 | ***Mean (SD)*** 76.9 (13.4) | ***Mean*** 1.1 |
| Rohde et al., 2014b | 41 | 24 y | ***Mean (SD)*** 1967 (593.6) ***Median (range)*** 1916 (857-3579) | ***Mean (SD)*** 31 (12.3) ***Median (range)*** 30 (14-67) | ***Mean (SD)*** 1424 (711.3) ***Median (range)*** 1160 (618-3055) | ***Mean (SD)*** 23 (13.1) ***Median (range)*** 19 (8-74) | ***Mean (SD)*** 36 (18.1) ***Median (range)*** 28 (15-80) | ***Mean (SD)*** 0.56 (0.31) ***Median (range)*** 0.46 (0.23-1.74) | ***Mean (SD)*** 41 (16.2) ***Median (range)*** 43 (5-70) | ***Mean (SD)*** 0.64 (0.26) ***Median (range)*** 0.62 (0.07-1.24) | ***Mean (SD)*** 77 (21.6) ***Median (range)*** 79 (30-133) | ***Mean (SD)*** 1.2 (0.36) ***Median (range)*** 1.15 (0.42-2.29) |
| Rocha et al., 2012 & 2013 | 25 | 22 y | N/A | | N/A | | ***Mean (SD)*** 33.62 (24.86) ***Median (range)*** 23.7 (12.6-104.2) | ***Mean (SD)*** 0.54 (0.36) ***Median (range)*** 0.42 (0.24-1.77) | ***Mean (SD)*** 53.6 (19.11) ***Median (range)*** 57.6 (9.33-89.4) | ***Mean (SD)*** 0.89 (0.34) ***Median (range)*** 0.95 (0.17-1.46) | ***Mean (SD)*** 86.7 (1.44) ***Median (range)*** 80.4 (38.1-141.4) | ***Mean (SD)*** 1.44 (0.35) ***Median (range)*** 1.37 (0.68-2.27) |

Abbreviations: PKU, phenylketonuria; N, number of patients in the age group; mo, months; y, years; SD, standard deviation; N/A, not available.

**Supplementary Table S5.** Dietary prescriptions of the patients with PKU in the included studies.

| Reference | N | Age | Dietary prescriptions | | | | | | | |
|---|---|---|---|---|---|---|---|---|---|---|
| | | | Phenylalanine | | Natural protein | | Protein equivalent from protein substitute | | Total protein | |
| | | | *mg/day* | *mg/kg/day* | *g/day* | *g/kg/day* | *g/day* | *g/kg/day* | *g/day* | *g/kg/day* |
| Evans et al., 2018 | | | N/A | | ***Median (range)*** | ***Median (range)*** | ***Median*** | ***Median*** | ***Median (range)*** | ***Median (range)*** |
| | 7 | 3 mo | | | 4 (3-5) | 0.6 (0.4-0.8) | 12 | 1.9 | 16 (15-18) | 2.5 (1.8-2.8) |
| | 19 | 4 mo | | | 4 (3-8) | 0.6 (0.4-1.0) | 14 | 2.2 | 19 (11-25) | 2.7 (1.3-3.7) |
| | 24 | 5 mo | | | 4 (3-8) | 0.6 (0.4-0.9) | 17 | 2.4 | 21 (17-27) | 2.9 (2.2-4.1) |
| | 30 | 6 mo | | | 4 (3-8) | 0.5 (0.3-1.0) | 20 | 2.5 | 23 (13-29) | 3.0 (2.1-4.2) |
| | 31 | 7 mo | | | 4 (3-8) | 0.5 (0.3-1.0) | 22 | 2.8 | 26 (17-33) | 3.2 (2.3-4.2) |
| | 31 | 8 mo | | | 4 (3-8) | 0.5 (0.3-1.0) | 24 | 2.9 | 27 (18-34) | 3.3 (2.6-4.2) |
| | 31 | 9 mo | | | 4 (3-8) | 0.5 (0.3-1.0) | 25 | 3.0 | 29 (21-34) | 3.3 (2.7-3.9) |
| | 31 | 10 mo | | | 4 (3-8) | 0.4 (0.3-1.0) | 27 | 3.0 | 30 (23-35) | 3.3 (2.6-4.0) |
| | 31 | 11 mo | | | 4 (3-8) | 0.4 (0.3-1.0) | 28 | 3.0 | 30 (23-40) | 3.3 (2.4-4.0) |
| | 31 | 12 mo | | | 4 (3-9) | 0.4 (0.3-1.0) | 30 | 3.1 | 31 (23-50) | 3.3 (2.4-4.3) |
| | 31 | 13 mo | | | 4 (3-8) | 0.4 (0.3-1.0) | 32 | 3.2 | 32 (23-45) | 3.3 (2.7-4.2) |
| | 31 | 14 mo | | | 4 (3-11) | 0.4 (0.3-1.0) | 32 | 3.2 | 33 (26-47) | 3.3 (2.6-4.2) |
| | 31 | 15 mo | | | 4 (3-11) | 0.4 (0.3-1.0) | 34 | 3.1 | 34 (26-45) | 3.3 (2.6-4.2) |
| | 31 | 16 mo | | | 4 (3-11) | 0.4 (0.2-1.0) | 33 | 3.2 | 34 (26-45) | 3.2 (2.4-3.7) |
| | 31 | 17 mo | | | 4 (3-11) | 0.4 (0.2-1.0) | 34 | 3.2 | 34 (27-45) | 3.2 (2.4-3.7) |
| | 31 | 18 mo | | | 4 (3-11) | 0.4 (0.2-1.0) | 35 | 3.2 | 35 (29-45) | 3.2 (2.4-3.7) |
| | 31 | 19 mo | | | 4 (3-11) | 0.4 (0.2-1.0) | 37 | 3.3 | 35 (29-45) | 3.2 (2.4-3.7) |
| | 30 | 20 mo | | | 4 (3-11) | 0.4 (0.2-1.0) | 38 | 3.3 | 36 (29-46) | 3.2 (2.4-3.8) |
| | 30 | 21 mo | | | 4 (3-11) | 0.4 (0.2-1.0) | 37 | 3.4 | 37 (29-46) | 3.3 (2.5-3.7) |
| | 29 | 22 mo | | | 4 (3-11) | 0.4 (0.2-1.0) | 39 | 3.4 | 37 (29-46) | 3.2 (2.5-3.7) |
| | 29 | 23 mo | | | 4 (3-11) | 0.4 (0.2-1.0) | 37 | 3.2 | 37 (29-46) | 3.2 (2.5-3.7) |
| | 29 | 24 mo | | | 4 (3-11) | 0.4 (0.2-0.9) | 37 | 3.3 | 38 (29-46) | 3.2 (2.5-3.7) |
| Evers et al., 2018 | | | | | ***Mean (SD)*** | ***Mean (SD)*** | ***Mean (SD)*** | ***Mean (SD)*** | ***Mean (SD)*** | ***Mean (SD)*** |
| | *BH4*: 18[b] | 11.2 y | | | | 0.41 (0.21) | | 1.05 (0.47) | | 1.46 (0.68) |
| | *Diet*: 19 | 13.0 y | | | 9.50 (9.96) | 0.35 (0.26) | 40.80 (20.91) | 0.98 (0.31) | 50.30 (30.87) | 1.33 (0.57) |
| | *Diet*: 17 | 18.0 y | | | 14.98 (10.00) | 0.25 (1.17) | 54.30 (16.96) | 0.91 (0.29) | 69.28 (26.96) | 1.16 (1.46) |
| | | | | | ***Median (range)*** | ***Median (range)*** | ***Median (range)*** | ***Median (range)*** | ***Median (range)*** | ***Median (range)*** |
| | *BH4*: 18 | 11.2 y | | | | 0.38 (0.15-0.94) | | 0.96 (0.18-1.94) | | 1.34 |
| | *Diet*: 19 | 12 .0 y | | | | 0.28 (0.08-0.98) | 40.80 (14.20-85.40) | 1.00 (0.43-1.52) | 50.30 | 1.28 |
| | *Diet*: 17 | 13.0 y | | | 9.50 (5.20-35.0) | 0.19 (0.07-0.63) | 60.00 (18.0-80.0) | 0.96 (0.31-1.45) | 70.50 | 1.15 |

**Supplementary Table S5.** Dietary prescriptions of the patients with PKU in the included studies.

| Reference | N | Age | Dietary prescriptions | | | | | | | |
|---|---|---|---|---|---|---|---|---|---|---|
| | | | Phenylalanine | | Natural protein | | Protein equivalent from protein substitute | | Total protein | |
| | | | *mg/day* | *mg/kg/day* | *g/day* | *g/kg/day* | *g/day* | *g/kg/day* | *g/day* | *g/kg/day* |
| | | | | | 10.50 (6.0-35.0) | | | | | |
| Ferguson et al., 1996 | 12 | 9-15 y | ***Mean (SD)***<br>373.3 (40.4)<br>***Median (range)***<br>350 (350-420) | N/A | N/A | | N/A | | N/A | |
| Gökmen-Özel et al., 2011 | 14 | 6 y | ***Mean (range)***<br>250 (150-1500) | ***Mean (range)***<br>10.4 (6.3-62.5) | N/A | | N/A | | N/A | |
| Green et al., 2019 | 16 | 29.5 y | N/A | | N/A | | ***Mean (SD)***<br>59 (9)<br>**Median (range)**<br>60 (40-80) | ***Mean***<br>0.84<br>**Median**<br>0.84 | N/A | |
| MacDonald et al., 1996 | 2<br>8<br>5<br>4<br>19 | 0-1 y<br>2-5 y<br>6-10 y<br>11 y <<br>Total median age: 4y | N/A | | ***Mean (SD)***<br>5.0 (2.83)<br>3.8 (0.85)<br>6.8 (3.91)<br>12.8 (3.77)<br>6.6 (4.30)<br>***Median (range)***<br>5.0 (3-17) | N/A | N/A | | N/A | ***Mean (SD)***<br>3.0 (N/A)<br>2.5 (N/A)<br>2.0 (N/A)<br>1.5 (N/A)<br>2.2 (0.5)<br>***Median (range)***<br>2.5 (1.5-3.0) |
| MacDonald et al., 2003 | 9<br>7<br>16 | 2-5 y<br>6-11 y<br>Total median age: 4.5y | N/A | | ***Mean (SD)***<br>5.9 (3.3)<br>6.3 (1.4)<br>6.1 (2.6)<br>***Median (range)***<br>6.0 (3-14) | N/A | N/A | | N/A | ***Mean (SD)***<br>2.5 (N/A)<br>2.0 (N/A)<br>2.3 (0.3)<br>***Median (range)***<br>2.5 (2.0-2.5) |

**Supplementary Table S5.** Dietary prescriptions of the patients with PKU in the included studies.

| Reference | N | Age | Dietary prescriptions | | | | | | | |
|---|---|---|---|---|---|---|---|---|---|---|
| | | | Phenylalanine | | Natural protein | | Protein equivalent from protein substitute | | Total protein | |
| | | | *mg/day* | *mg/kg/day* | *g/day* | *g/kg/day* | *g/day* | *g/kg/day* | *g/day* | *g/kg/day* |
| Wendel et al., 1990 | | | ***Mean (SD)*** | ***Mean (SD)*** | | | | | | |
| | 137[a] | 6 mo | 251 (46) | 34 (7) | | | | | | |
| | 138 | 1 y | 262 (52) | 28 (7) | | | | | | |
| | 135 | 1.5 y | 275 (56) | 26 (6) | | | | | | ***Mean (SD)*** |
| | 137 | 2 y | 276 (56) | 23 (5) | | | | | | 2.26 (0.47) |
| | 105 | 2.5 y | N/A | 22 (5) | | | | | | 2.17 (0.42) |
| | 98 | 3 y | N/A | 21 (5) | N/A | | N/A | | N/A | 2.20 (0.40) |
| | 96 | 3.5 y | N/A | 19 (5) | | | | | | 2.20 (0.38) |
| | 89 | 4 y | N/A | 18 (5) | | | | | | N/A |
| | 78 | 4.5 y | N/A | 18 (5) | | | | | | |
| | 65 | 5 y | N/A | 17 (5) | | | | | | |
| | 55 | 5.5 y | N/A | 16 (4) | | | | | | |
| | 52 | 6 y | N/A | 15 (5) | | | | | | |
| van Spronsen et al., 2009 | | | ***Mean (SD)*** | ***Mean (SD)*** | | | | | | |
| | 58 | 6 mo | 253 (85) | 35 (13) | | | | | | |
| | 59 | 1 y | 280 (66) | 29 (7) | | | | | | |
| | 57 | 2 y | 316 (106) | 26 (9) | N/A | | N/A | | N/A | |
| | 56 | 3 y | 359 (171) | 25 (11) | | | | | | |
| | 59 | 5 y | 386 (170) | 21 (9) | | | | | | |
| | N/A | 10 y | 431 (178) | 14 (6) | | | | | | |

Abbreviations: PKU, phenylketonuria; N, number of patients in the age group; mo, months; y, years; SD, standard deviation; N/A, not available. [a] The number of patients for total protein intake at 6 months was 136. [b]There were two cohorts in this study: 1) sapropterin -treated group (N=21), and 2) diet-only treated group (N=19). Only data at baseline (pre- sapropterin treatment) were included in the analysis in the sapropterin -treated group.
